# Honeycomb‐Layered Oxides With Silver Atom Bilayers and Emergence of Non‐Abelian SU(2) Interactions

**DOI:** 10.1002/advs.202204672

**Published:** 2022-12-27

**Authors:** Titus Masese, Godwill Mbiti Kanyolo, Yoshinobu Miyazaki, Miyu Ito, Noboru Taguchi, Josef Rizell, Shintaro Tachibana, Kohei Tada, Zhen‐Dong Huang, Abbas Alshehabi, Hiroki Ubukata, Keigo Kubota, Kazuki Yoshii, Hiroshi Senoh, Cédric Tassel, Yuki Orikasa, Hiroshi Kageyama, Tomohiro Saito

**Affiliations:** ^1^ Research Institute of Electrochemical Energy National Institute of Advanced Industrial Science and Technology (AIST) 1‐8‐31 Midorigaoka Ikeda Osaka 563‐8577 Japan; ^2^ AIST‐Kyoto University Chemical Energy Materials Open Innovation Laboratory (ChEM‐OIL) Sakyo‐ku Kyoto 606‐8501 Japan; ^3^ Department of Engineering Science The University of Electro‐Communications 1‐5‐1 Chofugaoka Chofu Tokyo 182‐8585 Japan; ^4^ Tsukuba Laboratory Sumika Chemical Analysis Service (SCAS), Ltd. Tsukuba Ibaraki 300‐3266 Japan; ^5^ Department of Physics Chalmers University of Technology SE‐412 Göteborg Sweden; ^6^ Graduate School of Life Sciences Ritsumeikan University 1‐1‐1 Noji‐higashi Kusatsu Shiga 525‐8577 Japan; ^7^ Key Laboratory for Organic Electronics and Information Displays and Institute of Advanced Materials (IAM) Nanjing University of Posts and Telecommunications (NUPT) Nanjing 210023 China; ^8^ Department of Industrial Engineering National Institute of Technology (KOSEN) Ibaraki College, 866 Nakane Hitachinaka Ibaraki 312‐8508 Japan; ^9^ Department of Energy and Hydrocarbon Chemistry Graduate School of Engineering Kyoto University Nishikyo‐ku Kyoto 615‐8510 Japan

**Keywords:** aberration‐corrected transmission electron microscopy, argentophilic interactions, honeycomb‐layered oxides, silver bilayers, sub‐valent degenerate states

## Abstract

Honeycomb‐layered oxides with monovalent or divalent, monolayered cationic lattices generally exhibit myriad crystalline features encompassing rich electrochemistry, geometries, and disorders, which particularly places them as attractive material candidates for next‐generation energy storage applications. Herein, global honeycomb‐layered oxide compositions, Ag_2_ *M*_2_TeO_6_ (M=Ni,Mg,etc.) exhibiting Ag atom bilayers with sub‐valent states within Ag‐rich crystalline domains of Ag_6_ *M*_2_TeO_6_ and Ag‐deficient domains of ${\mathrm{Ag}}_{2-x}{\mathrm{Ni}}_{2}{\mathrm{TeO}}_{6}$ (0<x<2). The Ag‐rich material characterized by aberration‐corrected transmission electron microscopy reveals local atomic structural disorders characterized by aperiodic stacking and incoherency in the bilayer arrangement of Ag atoms. Meanwhile, the global material not only displays high ionic conductivity but also manifests oxygen‐hole electrochemistry during silver‐ion extraction. Within the Ag‐rich domains, the bilayered structure, argentophilic interactions therein and the expected Ag sub‐valent states (1/2+,2/3+, *etc*.) are theoretically understood via spontaneous symmetry breaking of SU(2)× U(1) gauge symmetry interactions amongst 3 degenerate mass‐less chiral fermion states, justified by electron occupancy of silver $4d_{z^{2}}$ and 5*s* orbitals on a bifurcated honeycomb lattice. This implies that bilayered frameworks have research applications that go beyond the confines of energy storage.

## Graphical abstract

This work reports a new class of honeycomb‐layered oxides (Ag_2_ *M*_2_TeO_6_ (*M* is a transition or alkaline‐earth metal)) comprising unconventional sub‐valent bilayered silver‐rich domains (Ag_6_ *M*_2_TeO_6_), as elucidated via aberration‐corrected transmission electron microscopy. Stability of the bilayers is rationalized by a new model— SU(2)× U(1) interactions between three degenerate states of silver metal—responsible for all the observed unconventional features.

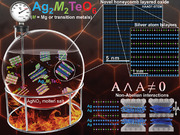

## 1 Introduction

Advancements in nanotechnology have unearthed a trove of multifunctional materials that promise to redefine the frontiers of research and applications with quixotic‐like physical, electrochemical, and structural functionalities.^[^ ^1^, ^2^ ^]^ Recent exemplars of these capabilities are embodied by honeycomb‐layered oxides, which exhibit unique electronic and magnetic behavior, fast ion kinetics, exotic geometries, and phase transitions,^[^ ^1^, ^2^ ^]^ alongside desirable electrochemical properties for energy storage applications.^[^ ^3^, ^4^, ^5^, ^6^, ^7^, ^8^ ^]^ In particular, these materials mainly feature a monolayer of monovalent or divalent atoms (such as Li, Na, K, Mg, Ba, Ag, and Cu), typically in a hexagonal (triangular) or honeycomb lattice, sandwiched between hexagonal or honeycomb transition metal—or heavy metal oxides, rendering them ideal for the design of next‐generation multifunctional materials. The honeycomb and/or hexagonal lattice is visible in the various two‐dimensional (2D) slices of the material since these materials, despite having different types of atoms, are constituted solely by the face‐centered cubic (FCC) packing and/or hexagonal close packing (HCP), which have been mathematically proven (assuming congruent spheres) to be the only optimized lattices in the three‐dimensional (3D) sphere packing problem.^[^ ^9^ ^]^ As a consequence, every 2D slice is either a hexagonal or honeycomb lattice, regardless of the type of atom constituting the slice one selects, unless there are deviations from the optimal condition, characterized by disclinations, distortions, dislocations, vacancies, and/or other topological features.^[^ ^10^ ^]^ Moreover, their unique crystalline structure and inherent structural symmetries facilitate 2D atomistic interactions to dominate the honeycomb‐layered heterostructures, which fosters the exploration not only of unconventional magnetic phenomena such as Heisenberg–Kitaev interactions^[^ ^1^, ^2^, ^11^ ^]^ but also new‐fangled emergent properties such as quantum geometries and topologies.^[^ ^12^, ^13^, ^14^, ^15^, ^16^ ^]^ Indeed, honeycomb‐layered tellurates (particularly, $A_{2}M_{2}{\mathrm{TeO}}_{6}$ (where A=Li,Na,K, etc. and M=Ni,Co,Mg,etc.) compositions) inferred experimentally^[^ ^17^, ^18^, ^19^, ^20^, ^21^, ^22^, ^23^, ^24^ ^]^ and from computations^[^ ^25^ ^]^ proffer a promising odyssey of probing into the functionalities of unchartered compositions that accommodate not only the aforementioned monolayer arrangement of monovalent or divalent atoms but also the possibility of multilayered structures of sub‐valent coinage metal atoms.^[^ ^30^, ^31^, ^32^, ^33^, ^34^, ^35^, ^36^ ^]^

Thus, the prospect of expounding the compositional diversity for honeycomb‐layered tellurates hosting coinage metal atoms (such as Ag,Cu, and Au) is poised to unlock new applications for this class of materials. Most notably, honeycomb‐layered tellurates that can accommodate a monolayer arrangement of Ag atoms have been envisioned to form structural coordinations that are very distinct from the typical prismatic and octahedral coordinations observed in alkali atoms (A compendium of the various slab arrangements (stackings) observed in honeycomb‐layered oxides is provided in the Supporting Information (Figure S1, Supporting Information)). In this vein, Ag atoms in Ag‐based honeycomb‐layered oxides such as ${\mathrm{Ag}}_{3}{\mathrm{Ni}}_{2}{\mathrm{SbO}}_{6}$, ${\mathrm{Ag}}_{3}{\mathrm{Ni}}_{2}{\mathrm{BiO}}_{6}$, amongst others have been reported to form dumbbell/linear coordinations with two oxygen atoms in varied stacking arrangements.^[^ ^1^, ^2^, ^37^, ^38^, ^39^, ^40^ ^]^ However, our interest in Ag‐based honeycomb‐layered tellurates was piqued by their propensity to adopt other variegated coordinations such as prismatic coordination, as predicted by theoretical studies.^[^ ^25^ ^]^ In particular, the possible formation of an assortment of Ag‐atom structures can be traced to their anomalous valency states (*i.e*., valency states of between 0 and 1 + (technically referred to as sub‐valent)), which have been posited to precipitate idiosyncratic structural and bonding properties when sandwiched between transition metal layers, such as the bilayer Ag atom arrangement observed in layered oxides such as in ${\mathrm{Ag}}_{2}MO_{2}$ (M=Cr,Co,Ni,etc.), amongst others.^[^ ^30^, ^31^, ^32^, ^33^, ^34^, ^35^, ^36^, ^41^, ^42^, ^43^, ^44^ ^]^

In a bid to gain insights into the peculiar structural dispositions of Ag‐atoms, we report for the first time the synthesis and structural characterisation of honeycomb‐layered tellurates with global compositions, Ag_2_ *M*_2_TeO_6_ (M=Ni,Mg, and other transition metal atoms) exhibiting Ag atom bilayers within Ag‐rich crystalline domains of Ag_6_ *M*_2_TeO_6_. Through aberration‐corrected scanning transmission electron microscopy, we report and elucidate the intricate atomic disordered structure of Ag_6_ *M*_2_TeO_6_, which is noted to predominantly comprise triangular Ag‐atom bilayer lattices sandwiched between transition metal slabs with an aperiodic stacking sequence. Electrochemical measurements reveal both ${\mathrm{Ag}}_{2}{\mathrm{Ni}}_{2}{\mathrm{TeO}}_{6}$ and ${\mathrm{Ag}}_{2}{\mathrm{Mg}}_{2}{\mathrm{TeO}}_{6}$ to display Ag‐ion extraction electrochemistry marked by a predominant formation of oxygen holes that debilitates reversible Ag‐ion electrochemistry. Nonetheless, these global compositions exhibit relatively high ionic conductivities of $2.39\times 10^{-2}$ and $3.84\times 10^{-4}\;\;S\;{\mathrm{cm}}^{-1}$, respectively at 100 °C—comparable to those of canonical Ag superionic conductors reported to date.^[^ ^45^, ^46^, ^47^, ^49^, ^50^, ^51^, ^52^, ^53^, ^54^, ^55^, ^56^, ^57^, ^58^, ^59^, ^60^, ^61^ ^]^

Finally, the bilayered structure observed in the Ag‐rich crystalline domains is theoretically understood by considering three degenerate mass‐less chiral fermion states of silver given by left‐handed states labeled by ${\mathrm{Ag}}_{+1/2}$ ($4d^{10}s^{1}$) and ${\mathrm{Ag}}_{-1/2}$ ($4d^{9}5s^{2}$) treated as emergent iso‐spin up (+1/2) and down (−1/2) degrees of freedom characteristic of special unitary group of degree 2 (SU(2)) gauge symmetry (responsible for Ag oxidation states ${\mathrm{Ag}}^{1+}$ ($4d^{10}5s^{0}$) and ${\mathrm{Ag}}^{1-}$ ($4d^{10}5s^{2}$), respectively) and a right‐handed oxidation state, ${\mathrm{Ag}}_{0}\to {\mathrm{Ag}}^{2+}$ ($4d^{9}5s^{2}\to 4d^{9}5s^{0}$) on the honeycomb lattice, based on the occupancy of their $4d_{z^{2}}$ and 5*s* orbitals. Note that ${\mathrm{Ag}}_{-1/2}$ and ${\mathrm{Ag}}_{0}$ are degenerate with essentially the same electronic state, $4d^{9}5s^{2}$, albeit form different oxidation states. Moreover, the oxidation states in the superscript also correspond to their respective valency states, achieved by a broken local SU(2)× U(1) gauge symmetry.^[^ ^62^ ^]^ Here, unitary group of degree 1 (U(1)) signifies the electromagnetic/Maxwell theory responsible for the electric charges of all the degenerate Ag states, whereas SU(2) is the emergent gauge group of the additional interaction between the left‐handed degenerate states, characteristic of sd‐hybridization, and analogous to lepton interactions in electroweak theory.^[^ ^62^, ^63^ ^]^ Breaking this symmetry introduces effective (sub‐valent) states such as 1/2+ and 2/3+ and a mass term between ${\mathrm{Ag}}^{2+}/{\mathrm{Ag}}_{0}$ and ${\mathrm{Ag}}^{1-}/{\mathrm{Ag}}_{-1/2}$, computed as the $\mathrm{Ag}-{\mathrm{Ag}}^{\prime }$ argentophilic interaction responsible for stabilizing the observed bilayered structure, leaving ${\mathrm{Ag}}^{1+}/{\mathrm{Ag}}_{+1/2}$ mass‐less. Other considerations such as modular and conformal symmetry^[^ ^10^, ^13^ ^]^ shed light on the nature of the bilayer, also observed in the other frameworks such as ${\mathrm{Ag}}_{2}^{1/2+}F^{1-}$, ${\mathrm{Ag}}_{2}^{1/2+}{\mathrm{Ni}}^{3+}O_{2}^{2-}$, and the hybrid, ${\mathrm{Ag}}_{3}^{2/3+}{\mathrm{Ni}}_{2}^{3+}O_{4}^{2-}$.^[^ ^30^, ^36^, ^64^, ^65^ ^]^ Ultimately, we regard the silver‐based honeycomb‐layered tellurate as a pedagogical platform for further inquiry into the role of geometric features and non‐commutative electromagnetic interactions, which go beyond energy storage applications.^[^ ^12^, ^13^ ^]^

Throughout the paper and the Supporting Information, we have adopted the notation: Ag_*x*_Ni_2_TeO_6_ where the global material composition (as ascertained by inductively‐coupled plasma atomic emission spectroscopy (ICP‐AES)) is given by x=2, Ag‐rich material is given by x=6, and whenever the material in the experiment is the hybrid of the two or indistinguishable, we have used the generic chemical formula, ${\mathrm{Ag}}_{x}{\mathrm{Ni}}_{2}{\mathrm{TeO}}_{6}$ with defined ranges of *x* given where possible/relevant. Moreover, whenever there are Ag vacancies present in the average material, we have referred to the material as Ag‐deficient, and used the chemical formula ${\mathrm{Ag}}_{2-x}{\mathrm{Ni}}_{2}{\mathrm{TeO}}_{6}$. With this notation, ${\mathrm{Ag}}_{2}{\mathrm{Ni}}_{2}{\mathrm{TeO}}_{6}$ is monolayered, Ag_*x*_Ni_2_TeO_6_ with 2≤x≤7, as ascertained by scanning transmission electron microscopy energy‐dispersive X‐ray spectroscopy (STEM‐EDX), is a mixture of monolayered and bilayered domains and ${\mathrm{Ag}}_{6}{\mathrm{Ni}}_{2}{\mathrm{TeO}}_{6}$ is bilayered.

## 2 Results

Given the tendency for silver‐containing materials to completely decompose at high temperatures under ambient pressures, conventional solid‐state synthetic routes could not be used in this study.^[^ ^66^ ^]^ Therefore, silver‐based honeycomb–layered tellurates encompassing the global compositions Ag_2_ *M*_2_TeO_6_ (M=Ni,Mg,Co,Cu,Zn, and ${\mathrm{Ni}}_{0.5}{\mathrm{Co}}_{0.5}$) were synthesized via a low‐temperature topochemical ion‐exchange reaction, as explicated in the Experimental Section. The elemental concentrations of the Ag_2_ *M*_2_TeO_6_ compositions were confirmed to be in line with the proprietary compositions of Ag_2_ *M*_2_TeO_6_ using inductively coupled plasma atomic emission spectroscopy (ICP‐AES), as provided in Supporting Information (Table S1, Supporting Information). The stoichiometry and homogeneous elemental distribution of the Ag_2_ *M*_2_TeO_6_ materials were further verified using energy‐dispersive X‐ray spectroscopy (EDX), as shown in the Supporting Information (Figures S2–S4, Supporting Information).

To ascertain the grain size and morphology of the crystal structures, the as‐prepared samples were subjected to scanning electron microscopy (SEM), which revealed a uniform distribution of micrometric‐sized particles (Figures S2–S4, Supporting Information). The grains were also observed to assume flake‐like (lamellar‐like) shapes—in character with other layered oxides.^[^ ^22^, ^24^ ^]^ The crystallinity and purity of the samples were ascertained through conventional X‐ray diffraction (XRD) analyses as shown in Figure S5 (Supporting Information). From the XRD patterns, no peaks attributed to the initial precursors or impurities were detected, indicating the high purity content of the samples prepared. Even so, the Bragg peaks in the patterns were broad and asymmetric (Figures S5 and S6a, Supporting Information), making it difficult to precisely validate the crystal structures. Furthermore, some Bragg reflections appeared to merge with the background, ruling out the possibility of accurately modeling the peak shapes. In an attempt to obtain a detailed structural characterization, synchrotron XRD (SXRD) data were obtained from one of the samples, ${\mathrm{Ag}}_{2}{\mathrm{Ni}}_{2}{\mathrm{TeO}}_{6}$ (Figure S6b, Supporting Information). The material was, however, found to have undergone SXRD‐induced damage, rendering this analytical route inapplicable for the present study.

**Figure 1:**
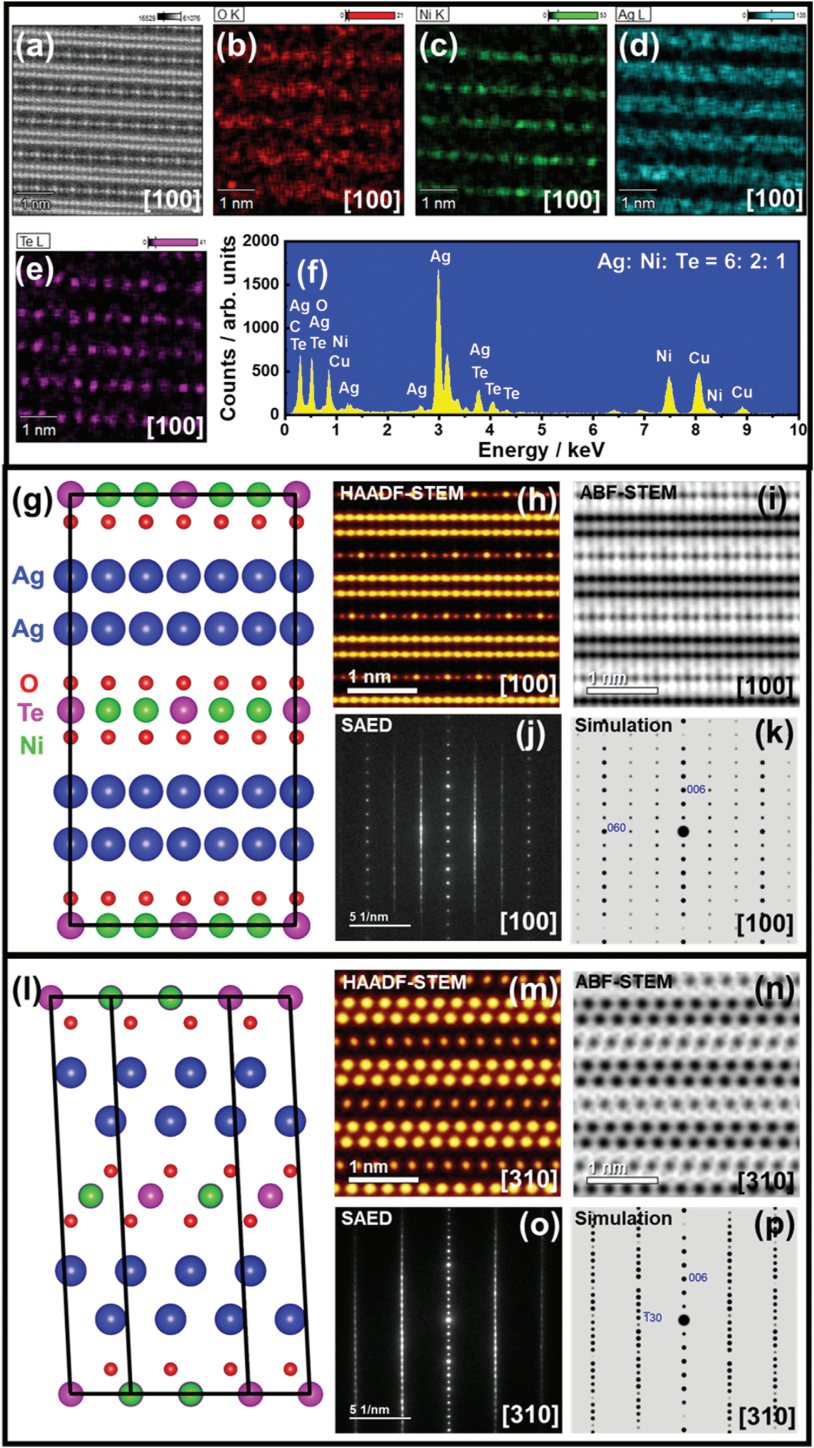
Visualization of bilayered domains of the Ag‐rich ${\mathrm{Ag}}_{6}{\mathrm{Ni}}_{2}{\mathrm{TeO}}_{6}$ within the global composition, ${\mathrm{Ag}}_{2}{\mathrm{Ni}}_{2}{\mathrm{TeO}}_{6}$. a) High‐angle annular dark‐field transmission electron microscopy (HAADF‐STEM) images of ${\mathrm{Ag}}_{6}{\mathrm{Ni}}_{2}{\mathrm{TeO}}_{6}$ nanocrystal taken along the [100] zone axis. Ag atom layers (large, dark spots) observed to be sandwiched between slabs of Te and Ni (small, light spots) coordinated with O atoms. b—e) Corresponding STEM‐energy dispersive spectroscopy (EDS) mapping showing the arrangement of constituent elements (O,Ni,Ag, and Te). f) STEM‐EDS spectra for a selected section (shown in panel(a)) confirming the elemental composition of the Ag‐rich domain, ${\mathrm{Ag}}_{6}{\mathrm{Ni}}_{2}{\mathrm{TeO}}_{6}$. The Cu and C spectra detected emanated from the TEM holder. g) Atomistic model of the average structure of ${\mathrm{Ag}}_{6}{\mathrm{Ni}}_{2}{\mathrm{TeO}}_{6}$ derived using STEM analyses along the [100] zone axis. Black lines are used to depict the partial unit cell. h) HAADF‐STEM image, showing Ag atom bilayers sandwiched between slabs comprising Ni and Te atoms. i) Annular bright‐field (ABF) image, affirming the atomic positions of oxygen atoms. j) Selected area electron diffraction (SAED) patterns taken along the [100] zone axis revealing spot shifts and streaks that suggest the existence of aperiodicity in line with the shifts in the transition metals slabs observed in panels (h) and (i). k) Corresponding kinematic simulations based on the structural model shown in panel (a). l) Atomistic model of ${\mathrm{Ag}}_{6}{\mathrm{Ni}}_{2}{\mathrm{TeO}}_{6}$ derived using STEM analyses along the [310] zone axis. The black lines are used to depict the partial unit cell. An alternating arrangement in the orientation of oxygen atoms can be seen in the subsequent transition metal atom slabs. m) HAADF‐STEM image, showing Ag atom bilayers sandwiched between slabs containing Te atoms. Ni atoms are superimposed on the position of the Te atoms. n) Annular bright‐field (ABF) image, affirming the alternating orientation of oxygen atoms in successive slabs. o) Selected area electron diffraction (SAED) patterns taken along the [310] zone axis revealing spot shifts and streaks that suggest the existence of aperiodicity. p) Corresponding kinematic simulations based on the structural model shown in panel (l).

It is worth noting that the appearance of significantly broadened peaks in the present Ag_2_ *M*_2_TeO_6_ compositions suggests the existence of defects or disorders in the slab stackings of the layered materials, as has been noted in related honeycomb‐layered oxides such as ${\mathrm{Ag}}_{3}{\mathrm{Ni}}_{2}{\mathrm{BiO}}_{6}$, ${\mathrm{Ag}}_{3}{\mathrm{Co}}_{2}{\mathrm{SbO}}_{6}$ and ${\mathrm{NaKNi}}_{2}{\mathrm{TeO}}_{6}$.^[^ ^14^, ^38^, ^67^ ^]^ Therefore, to explicitly visualize the emergent stacking sequences and honeycomb ordering of the Ag_2_ *M*_2_TeO_6_ samples without compromising their structural integrity, aberration‐corrected scanning transmission electron microscopy (STEM) was employed, as illustrated in **Figure** **1**. A high‐angle annular dark‐field (HAADF) STEM image obtained along the [100] zone axis (Figure 1a) shows an array of darker spots (Ag atoms) sandwiched between thinner planes of Te‐ and Ni‐ atom planes (light spots). This atomic arrangement is validated through elemental mapping by STEM‐EDX, as shown in Figure 1b–e. The elemental composition of constituent elements in the observed crystallite domain was further ascertained by STEM‐EDX spectra (Figure 1f) to be in the ratio of 6:2:1 for Ag, Ni, and Te, respectively, crucially revealing a Ag‐rich ${\mathrm{Ag}}_{6}{\mathrm{Ni}}_{2}{\mathrm{TeO}}_{6}$ crystalline domain. For ease of reference, the contrast (*I*) of the HAADF‐STEM image in Figure 1a is proportional to the atomic number (*Z*) of elements and their atomic arrangement (where $I\propto Z^{1.7}\approx Z^{2}$)^[^ ^68^, ^69^, ^70^ ^]^ as shown in Figure 1h. The image clearly displays a bilayer plane of Ag atoms (Z=47), marked by the larger and brighter golden spots, positioned between the layers of Te atoms (Z=52) denoted by the smaller golden spots, and Ni atoms (Z=28) represented by the darker amber spots. The corresponding annular bright‐field (ABF) STEM images (Figure 1i) is obtained to highlight the position of oxygen atoms in the crystal structure. As for ABF‐STEM images, $I\propto Z^{1/3}$,^[^ ^68^, ^69^, ^70^ ^]^ which means that elements with lighter atomic mass such as O (Z=8) can be visualized. For a clear visualization of the structural configuration of the Ag‐rich crysalline domain of ${\mathrm{Ag}}_{6}{\mathrm{Ni}}_{2}{\mathrm{TeO}}_{6}$, Figure 1g illustrates a crystal structure model rendered from the STEM images along the [100] zone axis. Here, the Te−Ni−Ni−Te sequential arrangement of the Te atoms and Ni atoms, typical amongst honeycomb structures, is clearly visualized. The atomic arrangements discerned by the STEM analyses were additionally corroborated by selected area electron diffraction (SAED) measurements taken along the [100] zone axis. As shown in Figure 1j, the atoms appear to align in a ‘streak‐like’ array of spots *in lieu* of distinctly separated spots, indicating the existence of a stacking disorder(s) (fault) across the slab (along the *c*‐axis). These results are further validated using kinematically simulated electron diffraction patterns (Figure 1k), which show consistency with the experimentally obtained SAED patterns. The atomic arrangement of atoms when viewed along the [100] zone axis is shown in Figure 1l.

To shed light on the oxygen‐atom positions in the crystallite, HAADF‐ and ABF‐STEM images of the ${\mathrm{Ag}}_{6}{\mathrm{Ni}}_{2}{\mathrm{TeO}}_{6}$ nanocrystal were obtained along the [310] zone axis as shown in Figure 1m,n, respectively. The oxygen atoms appear to be arranged diagonally in a zig‐zag orientation along the *c*‐axis—an orientation similar to those registered by the precursor materials prior to the topotactic ion exchange. The atomic arrangement along the [310] zone axis is confirmed using experimentally obtained SAED patterns (Figure 1o) and their corresponding kinematically simulated electron diffraction patterns (Figure 1p). The patterns obtained along the [310] zone axis manifests atomic streaks resembling those derived along the [100] zone axis, further substantiating the existence of stacking variants (faults or disorders) across the slabs. As such, an extensive examination of the stacking sequences is still necessary to garner deeper insight into their crystallographic information.

**Figure 2:**
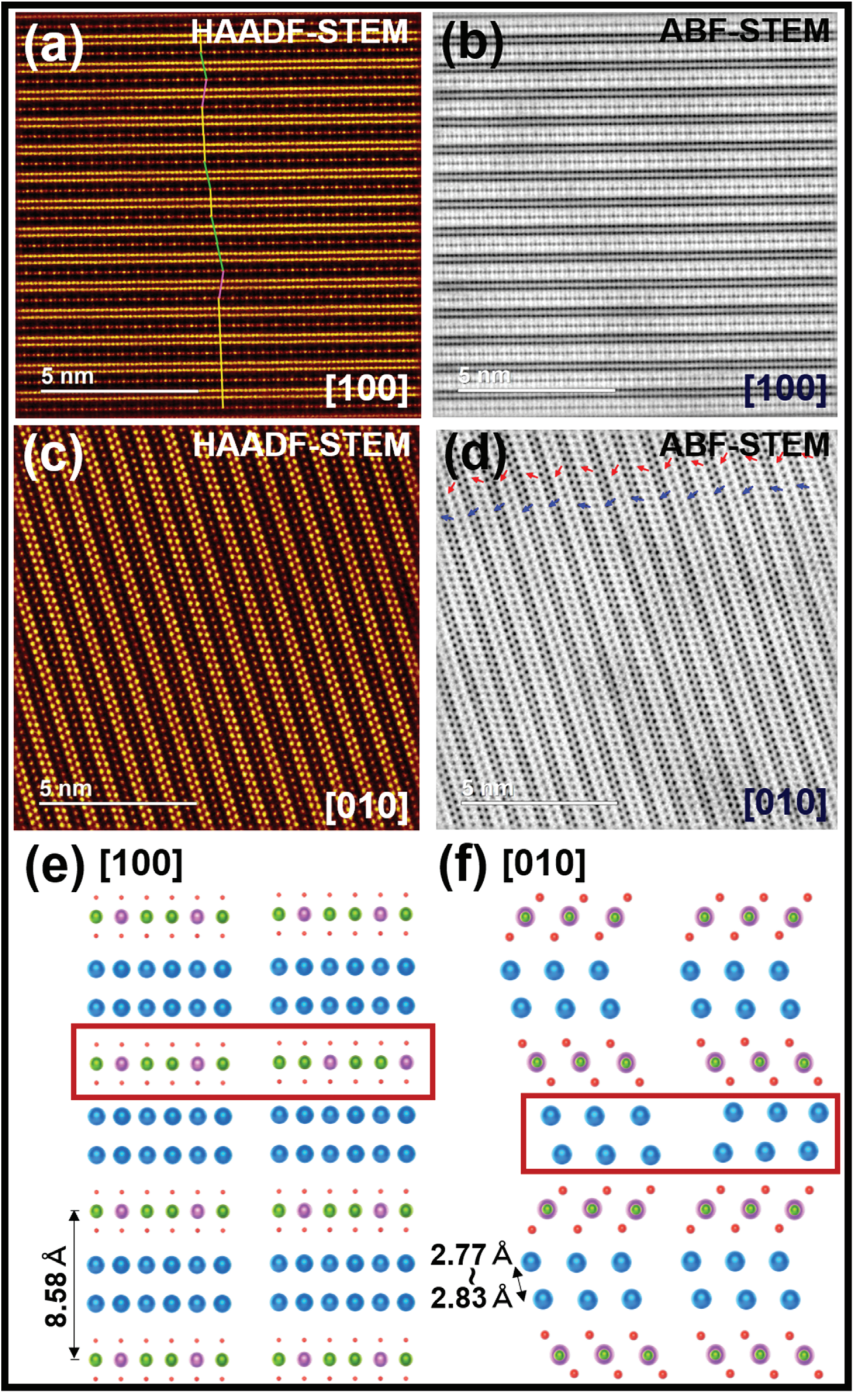
High‐resolution STEM imaging of Ag atom bilayers along multiple zone axes. a) HAADF‐STEM image of ${\mathrm{Ag}}_{6}{\mathrm{Ni}}_{2}{\mathrm{TeO}}_{6}$ crystallite taken along [100] zone axis showing the aperiodic ordering sequence of Ni and Te atoms in successive slabs and b) Corresponding ABF‐STEM image. c) Visualisation (along the [010] zone axis) using HAADF‐STEM, and d) Corresponding ABF‐STEM image, showing aperiodicity also in the arrangement of Ag in their respective sites across the slabs (or along the *c*‐axis). The red arrows highlight a periodic sequence of orientation of oxygen atoms across the slab and the blue arrows show shifts in the alignment of the Ag atom bilayer along the ab plane. e) Various arrangements of Ag atoms in ${\mathrm{Ag}}_{6}{\mathrm{Ni}}_{2}{\mathrm{TeO}}_{6}$ observed along the *b*‐axis. f) Shifts in the arrangement of Ni and Te atom slabs along the *c*‐axis. Silver atoms are shown in blue. Oxygen atoms are shown in red, whereas Ni and Te atoms are shown in green and pink, respectively.

Accordingly, to ascertain honeycomb ordering and the nature of stacking variations in the crystallite, the samples were subjected to high‐magnification STEM analyses, as illustrated in **Figure** **2**. In ordered honeycomb layered tellurate structures, the Te atoms (smaller golden spots) are typically positioned directly below or above the adjacent slabs in idyllic vertical arrays. However, the HAADF‐STEM images taken along the [100] zone axis (Figure 2a) reveal that in certain domains, the slabs deviate laterally from the ‘optimal’ arrays (as highlighted by the green and pink lines), indicating the occurrence of stacking faults across the slab stacking direction (*c*‐axis). For clarity, the right and left shifts of the Ni/Te atom slabs are denoted by green and pink lines, respectively. The corresponding ABF‐STEM image (Figure 2b) further underpin the shifts of Te/Ni atom slabs, albeit not as discernible as that of the HAADF‐STEM image. Similar aperiodic shifts in the Ni/Te slabs are also observed in the STEM images taken along the [110] zone axes, as shown in Figure S7 (Supporting Information). The occurrence of multiple disorders involving shifts in the metal slab layers along the *c*‐axis not only reflects the diversity of the disorders intrinsic in ${\mathrm{Ag}}_{6}{\mathrm{Ni}}_{2}{\mathrm{TeO}}_{6}$ but may also be envisioned to induce other disorders in the arrangement of Ag atom bilayers.

To investigate the occurrence of disorders in the Ag atom bilayers, HAADF‐ and ABF‐STEM images were taken along the [010] zone axis (Figure 2c,d). Although the orientation of the oxygen atoms across the Te/Ni slabs appears to follow a periodic sequence across the slab (as highlighted by red arrows in Figure 2d), the alignment of the Ag atom bilayer is seen to shift along the ab plane (perpendicular to the *c*‐axis) as indicated by the blue arrows. Here, the orientation of adjacent Ag bilayer planes is observed to frequently invert with no periodicity across the slabs, indicating a lack of coherency in their orientation along the *c*‐axis. Crystal schematic illustrations are provided in Figure 2e,f for an extensive review of the disorders in the arrangement of the transition metal slabs and the orientation of the Ag atom bilayers along the slab. The ${\mathrm{Ag}}_{6}{\mathrm{Ni}}_{2}{\mathrm{TeO}}_{6}$ crystallite appears vastly disordered with no coherence between the stacked transition metal layers and the silver atoms. Similar, observations were made across the global honeycomb layered oxide compositions, Ag_2_ *M*_2_TeO_6_ presented in this study (see Figures S8–S14, Supporting Information). Indeed, the variegation of structural defects/disorders visualized in the atomic resolution images are far beyond the reach of diffraction measurements, ratifying the need for high‐resolution STEM in the exploration of similar layered materials.

## 3 Discussion

### 3.1 Experimental Considerations

Herein, we report for the first time, silver‐based honeycomb‐layered tellurates embodying global compositions, Ag_2_ *M*_2_TeO_6_ (where M=3d transition metals or *s*‐block elements such as Mg) synthesized via topochemical ion‐exchange. Atomic‐resolution STEM analyses conducted along multiple zone axes reveal these tellurates, i.e., ${\mathrm{Ag}}_{2}{\mathrm{Ni}}_{2}{\mathrm{TeO}}_{6}$, ${\mathrm{Ag}}_{2}{\mathrm{Mg}}_{2}{\mathrm{TeO}}_{6}$, ${\mathrm{Ag}}_{2}{\mathrm{Co}}_{2}{\mathrm{TeO}}_{6}$, ${\mathrm{Ag}}_{2}{\mathrm{Cu}}_{2}{\mathrm{TeO}}_{6}$, and ${\mathrm{Ag}}_{2}{\mathrm{NiCoTeO}}_{6}$ (Figure 1; Figures S8–S14, Supporting Information), to predominantly encompass, within their Ag‐rich domains (i.e., ${\mathrm{Ag}}_{6}{\mathrm{Ni}}_{2}{\mathrm{TeO}}_{6}$, ${\mathrm{Ag}}_{6}{\mathrm{Mg}}_{2}{\mathrm{TeO}}_{6}$, ${\mathrm{Ag}}_{6}{\mathrm{Co}}_{2}{\mathrm{TeO}}_{6}$, ${\mathrm{Ag}}_{6}{\mathrm{Cu}}_{2}{\mathrm{TeO}}_{6}$, and ${\mathrm{Ag}}_{6}{\mathrm{NiCoTeO}}_{6}$), silver atom bilayers interspersed between honeycomb slabs. These Ag‐atom bilayered tellurates were observed to engender crystallites with significantly larger interlayer distances and variegated structural disorders–attributes poised to propagate fascinating 2D interactions, phase transitions, and rapid cation diffusion within the materials. Amongst the numerous crystallites investigated, the global composition ${\mathrm{Ag}}_{2}{\mathrm{Ni}}_{2}{\mathrm{TeO}}_{6}$ exhibits Ag‐rich crystalline domains with interslab distances of ≈9 Å  with a manifold of structural disorders (Figure 2), making it the exemplar material of focus for this study. It is worth mentioning that to date, bilayered structures entailing Ag‐atoms have not been reported amongst honeycomb‐layered oxides, despite the rich structural diversity manifested by these materials. In fact, STEM analyses conducted on a bismuthate analogue, ${\mathrm{Ag}}_{3}{\mathrm{Ni}}_{2}{\mathrm{BiO}}_{6}$, prepared using the present synthesis protocols, demonstrate the crystallites to have a monolayered arrangement of monovalent Ag atoms with significantly smaller interlayer distances (see Figures S15–S17, Supporting Information). Moreover, a previous simulation result of Ag‐based honeycomb‐layered oxides^[^ ^25^ ^]^ using the Kohn–Sham formalism^[^ ^26^ ^]^ found only monolayered frameworks, indicative of the challenges and controversy faced by conventional and non‐conventional bonding schemes to effectively reproduce Ag‐based structures.^[^ ^27^, ^28^, ^29^ ^]^ As such, this study not only represents a major milestone in the exploration of honeycomb‐layered oxides functionalities but also expounds on the structural expedience of honeycomb layered tellurates (Figure S18, Supporting Information).

The material knowledgebase for compounds manifesting Ag‐atom bilayered structures remains limited.^[^ ^30^, ^31^, ^32^, ^33^, ^34^, ^35^, ^36^ ^]^ Thus, their occurrence in the present honeycomb‐layered tellurates betoken significant progress in the advancement of the crystal structural versatility of honeycomb‐layered oxides. Until now, the advancement of compounds manifesting Ag‐atom bilayers has been heavily curtailed by their stringent synthetic conditions, which typically involve giga‐Pascal scale pressures and synthesizing precursors under elevated oxygen pressures and temperatures.^[^ ^31^, ^32^, ^64^ ^]^ As an alternative route, this study employs a high molar silver salt‐to‐precursor ratio to develop these bilayered structures via a low‐temperature metathetic (topochemical ion‐exchange) synthetic route. Equivalent molar ratios of initial precursors and ${\mathrm{AgNO}}_{3}$ molten salt in the case of ${\mathrm{Ag}}_{2}{\mathrm{Ni}}_{2}{\mathrm{TeO}}_{6}$ were found to be insufficient in facilitating a complete ${\mathrm{Ag}}^{+}$ ion exchange (see Figure S19, Supporting Information). Although the resulting crystallites formed are predominantly Ag‐atom bilayered structures, defects in the arrangement of silver atoms were exhibited in some crystallites, characterized by the presence of Ag‐deficient domains with atoms in their amorphous state (single Ag atom layers) alongside Ag‐rich domains with Ag‐atom bilayers (Figures S20–S23, Supporting Information). This postulation was verified by TEM‐EDX measurements, which demonstrate Ag_2_ *M*_2_TeO_6_ compositions to have a rich global composition comprising Ag‐rich and Ag‐deficient regimes in close proximity.

**Figure 3:**
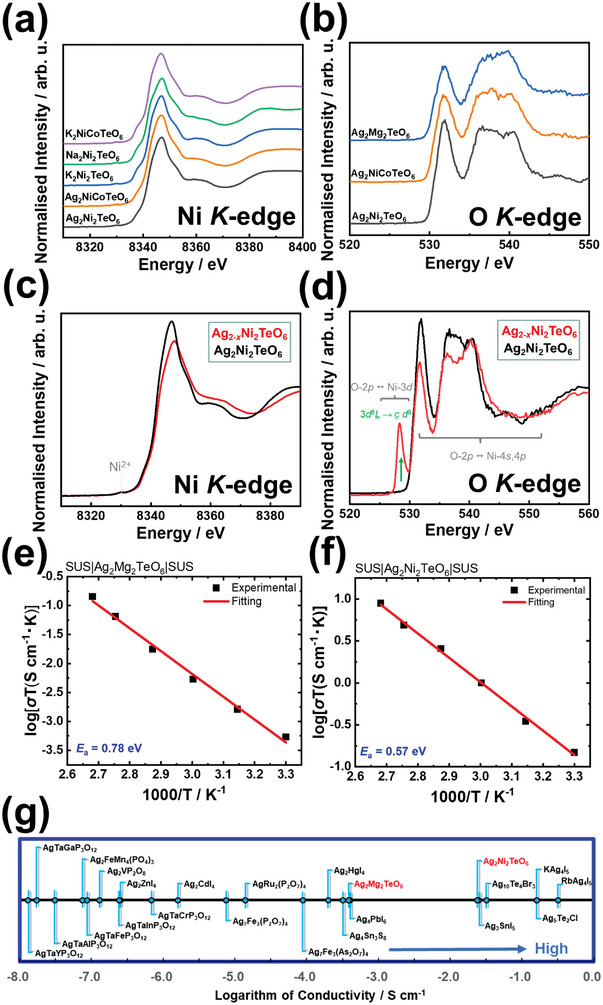
Spectroscopic measurements of the global compositions, Ag_2_ *M*_2_TeO_6_ (M=Mg and Ni). a) Normalized Ni *K*‐edge *ex situ* X‐ray absorption spectra (XAS) of ${\mathrm{Ag}}_{2}{\mathrm{Ni}}_{2}{\mathrm{TeO}}_{6}$ and related derivatives (${\mathrm{Ag}}_{2}{\mathrm{NiCoTeO}}_{6}$ and ${\mathrm{Ag}}_{2}{\mathrm{Mg}}_{2}{\mathrm{TeO}}_{6}$) collected along with reference ${\mathrm{Ni}}^{2+}$compounds (${\mathrm{Na}}_{2}{\mathrm{Ni}}_{2}{\mathrm{TeO}}_{6}$, $K_{2}{\mathrm{Ni}}_{2}{\mathrm{TeO}}_{6}$and $K_{2}{\mathrm{NiCoTeO}}_{6}$) and b) Normalized O *K*‐edge ex situ XAS spectra of Ag_2_ *M*_2_TeO_6_ (M=Mg,Ni and ${\mathrm{Ni}}_{0.5}{\mathrm{Co}}_{0.5}$) taken in fluorescence yield (FY) mode–highly sensitive to the innate bulk properties. c) Normalized Ni *K*‐edge ex situ XAS spectra of ${\mathrm{Ag}}_{2}{\mathrm{Ni}}_{2}{\mathrm{TeO}}_{6}$ pristine electrode and charged electrode (${\mathrm{Ag}}_{2-x}{\mathrm{Ni}}_{2}{\mathrm{TeO}}_{6}$). d) Normalized O *K*‐edge ex situ XAS spectra of ${\mathrm{Ag}}_{2}{\mathrm{Ni}}_{2}{\mathrm{TeO}}_{6}$ pristine electrode and charged electrode (${\mathrm{Ag}}_{2-x}{\mathrm{Ni}}_{2}{\mathrm{TeO}}_{6}$). Ligand holes are created in the O2p bands during Ag‐ion extraction. e) Arrhenius plots derived from electrochemical impedance spectroscopy (EIS) measurements of ${\mathrm{Ag}}_{2}{\mathrm{Mg}}_{2}{\mathrm{TeO}}_{6}$ and f) ${\mathrm{Ag}}_{2}{\mathrm{Ni}}_{2}{\mathrm{TeO}}_{6}$. g) Comparative plots of the ionic conductivity values attained in representative Ag‐based ionic conductors reported along with the honeycomb‐layered tellurates.^[^ ^45^, ^46^, ^47^, ^49^, ^50^, ^51^, ^52^, ^53^, ^54^, ^55^, ^56^, ^57^, ^58^, ^59^, ^60^, ^61^ ^]^

From a chemical perspective, the formation of Ag‐atom bilayered structures (as shown in Figures 2 and **3**) can be attributed to the tendency of Ag to assume anomalous valency states (also referred to as sub‐valent states) such as oxidation states between 0 and 1 + . As a result, the Ag ions aggregate to form atomic coordinations (i.e., $\mathrm{Ag}-{\mathrm{Ag}}^{\prime }$ bonds) resemblant of silver metal (metallic silver) topologies,^[^ ^30^, ^41^, ^42^, ^43^, ^44^ ^]^ which in principle violate conventional bonding mechanisms and electronic structures. In general, the constituent elements of the present global compositions can be assigned to the valency states of ${\mathrm{Ni}}^{2+}$, ${\mathrm{Ag}}^{1+}$, and ${\mathrm{Te}}^{6+}$ to yield a valency description of ${\mathrm{Ag}}_{2}^{1+}{\mathrm{Ni}}_{2}^{2+}{\mathrm{Te}}^{6+}O_{6}^{2-}$. To ascertain these valency states, X‐ray photoelectron spectra (XPS) of the ${\mathrm{Ag}}_{2}{\mathrm{Ni}}_{2}{\mathrm{TeO}}_{6}$ crystallite and its related derivatives (${\mathrm{Ag}}_{2}{\mathrm{NiCoTeO}}_{6}$ and ${\mathrm{Ag}}_{2}{\mathrm{Mg}}_{2}{\mathrm{TeO}}_{6}$) were obtained at the binding energies of Ag3d, Ni2p, and Te3d, as provided in Figures S24 and S25 (Supporting Information). Since the measurements were performed on the global compositions, the existence of Ag sub‐valent states within the Ag‐rich domains could not be unequivocally established using spectroscopic techniques such as XPS and XAS. In particular, since the as‐prepared material with a global composition of ${\mathrm{Ag}}_{2}{\mathrm{Ni}}_{2}{\mathrm{TeO}}_{6}$ (as ascertained by ICP‐AES) has an inseparable mixture of Ag‐deficient phases (such as ${\mathrm{Ag}}_{2-x}{\mathrm{Ni}}_{2}{\mathrm{TeO}}_{6}$, 0<x<2) and Ag‐rich (expected) sub‐valent phases such as ${\mathrm{Ag}}_{6}{\mathrm{Ni}}_{2}{\mathrm{TeO}}_{6}$, this necessitates one to perform STEM electron energy‐loss spectroscopy (EELS) on Ag‐rich nanocrystallite domains at the Ag *M*‐, Te *M*‐, Ni *L*‐ and O *K*‐edges in order to distinguish these phases, which proved challenging to conclusively perform in the present work. Meanwhile, the divalent nature of Ni (${\mathrm{Ni}}^{2+}$) is further corroborated through X‐ray absorption spectroscopy (XAS) performed on the global composition, ${\mathrm{Ag}}_{2}{\mathrm{Ni}}_{2}{\mathrm{TeO}}_{6}$ and its related derivatives (${\mathrm{Ag}}_{2}{\mathrm{NiCoTeO}}_{6}$ and ${\mathrm{Ag}}_{2}{\mathrm{Mg}}_{2}{\mathrm{TeO}}_{6}$) at the Ni *K*‐edge, as shown in Figure **3**a. Further, O *K*‐edge XAS spectra of ${\mathrm{Ag}}_{2}{\mathrm{Ni}}_{2}{\mathrm{TeO}}_{6}$ along with related tellurate compositions are taken in the bulk‐sensitive fluorescence yield mode to establish the valency of O atoms (Figure 3b). Here, no spectral features ascribed to oxygen hole formation were identified, indicating that the valency of oxygen does not contribute to the formation of the structures observed. Thus, since the valency states of the other metal elements in the Ag‐rich domains were ascertained to be ${\mathrm{Ni}}^{2+}$ (divalent) for Ni atoms, and mixed valency states of ${\mathrm{Te}}^{4+}$ and ${\mathrm{Te}}^{6+}$ for Te atoms, and the general chemical formula can be confirmed by the XPS and XAS results alongside the STEM‐EDX spectra to be given by ${\mathrm{Ag}}_{6}{\mathrm{Ni}}_{2}{\mathrm{TeO}}_{6}={\mathrm{Ag}}_{6}^{(1-\delta )+}{{\mathrm{Ni}}_{2}^{2+}\mathrm{Te}}_{x}^{4+}{\mathrm{Te}}_{y}^{6+}O_{6}^{2-}$ (x≥0, y≥0, x+y=1), Ag must be sub‐valent by the charge neutrality requirement (i.e., 6×(1−δ)+2×2+4x+6y+6×(2−)=0→2x+3y=1+3δ, together with x+y=1, are simultaneous equations that can be solved to yield, δ=(y+1)/3 thus giving the Ag sub‐valency range, 1/3+≤(1−δ)+≤2/3+ for 0≤y≤1).

Whilst we have successfully shown, by the charge neutrality argument constrained by the experimental data, that the Ag‐rich domain of the present material must comprise sub‐valent Ag cations, we cannot further determine the exact value of this sub‐valency that ought to lie within the bound, 1/3+≤(1−δ)+≤2/3+. Meanwhile, first‐principles computations (Figure S26 and Note S1, Supporting Information) suggest a complex valency composition encompassing an admixture of multiple valency states such as ${\mathrm{Ag}}_{6}^{1/3+}{\mathrm{Ni}}_{2}^{2+}{\mathrm{Te}}^{6+}O_{6}^{2-}$, ${\mathrm{Ag}}_{6}^{2/3+}{\mathrm{Ni}}_{2}^{2+}{\mathrm{Te}}^{4+}O_{6}^{2-}$, ${\mathrm{Ag}}_{6}^{1/2+}{\mathrm{Ni}}_{2}^{2+}{\mathrm{Te}}_{1/2}^{4+}{\mathrm{Te}}_{1/2}^{6+}O_{6}^{2-}$, and ${\mathrm{Ag}}_{4}^{1/2+}{\mathrm{Ni}}_{2}^{2+}{\mathrm{Te}}^{6+}O_{6}^{2-}$, which evince to the contribution of sub‐valent states of Ag atoms in the formation of silver atom bilayers.^[^ ^30^, ^31^, ^32^, ^33^, ^34^, ^35^, ^36^ ^]^ The snippets of structural information gathered in this study altogether allude to the rich global composition of the present tellurates, which entail Ag‐deficient domains with a valency description of ${{\mathrm{Ag}}^{1+}}_{2-x}{\mathrm{Ni}}_{2}^{(2+z/2)+}{\mathrm{Te}}^{(6-y)+}O_{6}^{2-}$ (z=x+y, 0≤x<2, 0≤y≤2) alongside Ag‐rich domains comprising an admixture of ${\mathrm{Ag}}_{4}^{1/2+}{\mathrm{Ni}}_{2}^{2+}{\mathrm{Te}}^{6+}O_{6}^{2-}$, ${\mathrm{Ag}}_{6}^{1/2+}{\mathrm{Ni}}_{2}^{2+}{\mathrm{Te}}_{1/2}^{4+}{\mathrm{Te}}_{1/2}^{6+}O_{6}^{2-}$, ${\mathrm{Ag}}_{6}^{2/3+}{\mathrm{Ni}}_{2}^{2+}{\mathrm{Te}}^{4+}O_{6}^{2-}$, etc. with varied atomic occupancies. In fact, such sub‐valent Ag bilayered frameworks have hitherto been reported in materials such as ${\mathrm{Ag}}_{2}^{1/2+}F^{1-}$, ${\mathrm{Ag}}_{3}^{2/3+}O^{2-}$, ${\mathrm{Ag}}_{2}^{1/2+}{\mathrm{Ni}}^{3+}O_{2}^{2-}$ and hybrids (alternating monolayers and bilayers) such as ${\mathrm{Ag}}_{3}^{2/3+}{\mathrm{Ni}}_{2}^{3+}O_{4}^{2-}$.^[^ ^30^, ^36^, ^41^, ^64^, ^65^ ^]^ A crucial observation is that these frameworks not only involve sub‐valent states (1/2+ or 2/3+, etc.) but also their Ag bilayers are formed by two triangular lattices of an apparent bifurcated bipartite honeycomb lattice. Meanwhile, in the case where there is a monolayered counterpart, e.g., ${\mathrm{Ag}}^{1+}{\mathrm{Ni}}^{3+}O_{2}^{2-}$, the Ag is not only monovalent but also the lattice is triangular and monolayered.^[^ ^30^, ^36^, ^64^, ^65^ ^]^ Indeed, this is the case for ${\mathrm{Ag}}_{3}^{1+}{\mathrm{Ni}}_{2}^{2+}{\mathrm{Bi}}^{5+}O_{6}^{2-}$, whereby the Ag atom is monovalent, and the lattice is not only monolayered but triangular,^[^ ^38^ ^]^ in contrast to the present material reported herein. Meanwhile, the Ag sub‐valent state 1/3+ remains conspicuously absent in reported layered materials^[^ ^30^, ^36^, ^64^ ^]^ and other silver‐rich oxides,^[^ ^41^, ^42^, ^43^, ^71^, ^72^, ^73^, ^74^, ^75^, ^76^, ^77^ ^]^ despite its presence reported, e.g., in ${\mathrm{Ag}}_{3}^{1/3+}O^{2-}H^{1+}$.^[^ ^78^ ^]^ Finally, although the sub‐valency of silver remains unascertained in the present material via direct XPS measurements of Ag binding energies, the fact that the Ag‐rich domains are bilayered with an apparent bifurcated bipartite honeycomb lattice demonstrates that such domains fit well within the aforementioned class of bilayered sub‐valent Ag‐based frameworks, thus corroborating the charge neutrality argument provided earlier for the sub‐valency of Ag. Further information on the sub‐valent nature of Ag in the bilayered domains could be garnered from a direct visualization of the local coordination of Ag atoms using high‐resolution STEM (Figure 1). The shortest $\mathrm{Ag}-{\mathrm{Ag}}^{\prime }$ distances in the bilayered ${\mathrm{Ag}}_{6}{\mathrm{Ni}}_{2}{\mathrm{TeO}}_{6}$ domains were found to be those between adjacent Ag atoms of subsequent layers (in the ranges of 2.77−2.83 Å   (*viz*., 2.80 ± 0.03 Å)), as highlighted in Figure 3f and Figure S27 (Supporting Information). These $\mathrm{Ag}-{\mathrm{Ag}}^{\prime }$ distances of ${\mathrm{Ag}}_{6}{\mathrm{Ni}}_{2}{\mathrm{TeO}}_{6}$ are akin to those of sub‐valent ${\mathrm{Ag}}_{2}^{1/2+}{\mathrm{NiO}}_{2}$ (2.836 Å) and ${\mathrm{Ag}}_{2}^{1/2+}F$ (2.814 Å),^[^ ^64^, ^76^ ^]^ suggesting a universal $\mathrm{Ag}-{\mathrm{Ag}}^{\prime }$ bonding mechanism for such bilayered and other related materials. For instance, this universality beyond layered materials is also exhibited by the bifurcated Ag honeycomb structure in ${\mathrm{Ag}}_{16}^{1/2+}B_{4}O_{10}$ (constituting a tetrahedral shape conjectured by the authors of Ref. [43] to habour excess localized electrons responsible for the reported Ag sub‐valency of 1/2+), with a bond length of order ≈2.8 Å.^[^ ^43^ ^]^

Considering the presence of mobile Ag cations sandwiched between transition metal slabs comprising highly electronegative ${\mathrm{Ni}}^{2+}$, empirical insight into the possibility of electrochemical extraction of Ag‐ions from the ${\mathrm{Ag}}_{2}{\mathrm{Ni}}_{2}{\mathrm{TeO}}_{6}$ structures would be invaluable in their future utility. Thus, the electrochemical performance of ${\mathrm{Ag}}_{2}{\mathrm{Ni}}_{2}{\mathrm{TeO}}_{6}$ electrode was investigated through cyclic voltammetry conducted on Ag half‐cells, as detailed in the **Methods** **Section**. The voltammograms obtained illustrate the occurrence of an oxidative peak at ≈1.4 V versus ${\mathrm{Ag}}^{+}$/Ag, pointing to the prospects of silver‐ion extraction at high voltages (see Figure S28, Supporting Information). However, no reduction peaks were observed, suggesting the occurrence of an irreversible phase transformation or structural deterioration (amorphization), which was further affirmed by the corresponding galvanostatic cycling tests. The amorphization/phase transformation of ${\mathrm{Ag}}_{2}{\mathrm{Ni}}_{2}{\mathrm{TeO}}_{6}$ is further evident in *ex situ* XRD measurements as shown in Figure S29 (Supporting Information). To investigate the atomistic mechanisms governing the silver‐ion extraction process in ${\mathrm{Ag}}_{2}{\mathrm{Ni}}_{2}{\mathrm{TeO}}_{6}$, *ex situ* XAS spectra were obtained from pristine and charged ${\mathrm{Ag}}_{2}{\mathrm{Ni}}_{2}{\mathrm{TeO}}_{6}$ electrodes at the Ni *K*‐ and O *K*‐edges. As shown in Figure 3c, no significant changes in the spectral features of the electrode are observed during silver‐ion extraction, an indication that Ni predominantly remains in the divalent state throughout the process. On the other hand, the O *K*‐edge XAS spectra (Figure 3d) displays a sharp increase in the intensity of the pre‐edge peak centered ≈528 eV during the charging process. This observation evinces that the extraction of silver ions from ${\mathrm{Ag}}_{2}{\mathrm{Ni}}_{2}{\mathrm{TeO}}_{6}$ is accompanied by a rapid formation of oxygen ligand holes.

In principle, Ag ion extraction can be rationalized to increase the valency state of nickel from ${\mathrm{Ni}}^{2+}$ to ${\mathrm{Ni}}^{3+}$, where ${\mathrm{Ni}}^{3+}$ has a predominant electronic ground state of 3*d* ^7^. However, the agitations in the Ni‐atom electronic configurations triggered by their hybridization with O‐2*p* orbitals engender a ground state characterized by the $3d^{8}\underline{L}$ orbital character (for clarity, $\underline{L}$ denotes the ligand hole)–akin to those observed in the charged states of compounds such as ${\mathrm{LiNiO}}_{2}$.^[^ ^79^ ^]^ Accordingly, the increased intensity observed in the pre‐edge peak at 528 eV during charging (Ag ion extraction) can be attributed to the transition into the $3d^{8}\underline{L}$ ground state. Thus, the core transitions during this process can be assigned as $3d^{8}\underline{L}\to \underline{c}d^{8}$. It is worth pointing out that although honeycomb‐layered oxides such as ${\mathrm{Li}}_{4}{\mathrm{FeSbO}}_{6}$ have been shown to exhibit reversible oxygen‐redox capabilities,^[^ ^80^ ^]^ the present spectroscopic and diffraction measurements indicate the rapid formation of oxygen holes that debilitate the structural integrity of ${\mathrm{Ag}}_{2}{\mathrm{Ni}}_{2}{\mathrm{TeO}}_{6}$ during silver‐ion extraction at high voltages.

*Nota bene*, the prominence gained by honeycomb‐layered oxides has to some extent been banked on the high voltage capabilities and fast ionic conductivities seen in materials such as ${\mathrm{Na}}_{2}{\mathrm{Ni}}_{2}{\mathrm{TeO}}_{6}$ and ${\mathrm{Na}}_{2}{\mathrm{Mg}}_{2}{\mathrm{TeO}}_{6}$.^[^ ^1^, ^2^, ^19^, ^81^, ^82^ ^]^ Therefore, investigating the ionic conductivities of their silver analogues (*i.e*., ${\mathrm{Ag}}_{2}{\mathrm{Ni}}_{2}{\mathrm{TeO}}_{6}$ and ${\mathrm{Ag}}_{2}{\mathrm{Mg}}_{2}{\mathrm{TeO}}_{6}$) under various temperature conditions would be an integral step in determining their innate capabilities. The compounds were subjected to thermal gravimetric analyses to ascertain their thermal stability (Figures S30 and S31, Supporting Information). Subsequently, their ionic conductivities at different temperatures were assessed, as shown by the Arrhenius plots in Figures 3e and 3f. Detailed experimental protocols are provided in the Experimental Section. In the temperature range of 30–100 °C, ${\mathrm{Ag}}_{2}{\mathrm{Ni}}_{2}{\mathrm{TeO}}_{6}$ (with a pellet compactness of ≈84 %) was determined to have an activation energy of ≈0.57 eV, which was calculated by fitting the alternating current data with the Arrhenius equation. The conductivity of the material, which predominantly emanates from ionic diffusion, was found to be $4.88\times 10^{-4}\;\;S\;{\mathrm{cm}}^{-1}$ at 30 °C and $2.39\times 10^{-2}\;\;S\;{\mathrm{cm}}^{-1}$ at 100 °C. On the other hand, ${\mathrm{Ag}}_{2}{\mathrm{Mg}}_{2}{\mathrm{TeO}}_{6}$ (with a pellet compactness of ≈74 %) displays a predominant ionic conductivity of $1.77\times 10^{-6}\;\;S\;{\mathrm{cm}}^{-1}$ at 30 °C and $3.84\times 10^{-4}\;\;S\;{\mathrm{cm}}^{-1}$ at 100 °C. For comparison, the bulk ionic conductivities of the silver‐based tellurates are presented alongside other reported silver‐ion superionic conductors in Figure 3g.^[^ ^45^, ^46^, ^47^, ^49^, ^50^, ^51^, ^52^, ^53^, ^54^, ^55^, ^56^, ^57^, ^58^, ^59^, ^60^, ^61^ ^]^ Until now, binary and ternary silver chalcogenides, silver chalcogenidehalides and silver polychalcogenides have dominated the list of materials with fast Ag‐ion conduction.^[^ ^45^, ^46^, ^47^, ^49^, ^50^, ^51^, ^52^, ^53^, ^54^, ^55^ ^]^ However, from these ion conductivity plots, it is apparent that the present class of honeycomb‐layered tellurates (with a global composition of Ag_2_ *M*_2_TeO_6_) confers relatively higher Ag‐ion conductivity in comparison. It is essential to highlight that Ag ion‐based layered oxide materials with high ionic conductivities have yet to be reported. Therefore, these results unveil new prospects of utilising Ag_2_ *M*_2_TeO_6_ honeycomb layered oxides compositions as feasible solid electrolytes for electrochemical devices such as all‐solid‐state Ag‐ion batteries.^[^ ^83^, ^84^, ^85^, ^86^, ^87^, ^88^ ^]^

### 3.2 Theoretical Considerations

**Figure 4:**
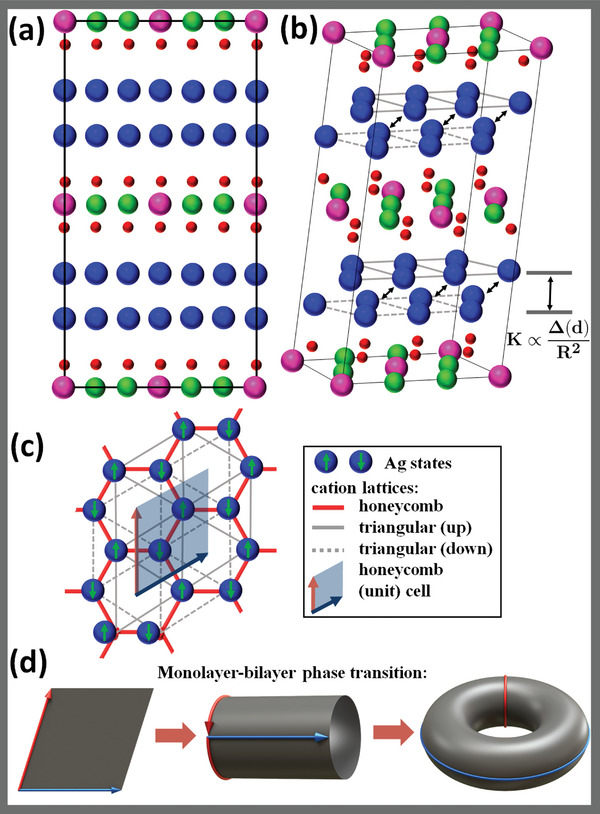
Structure, geometry and topological features in the silver‐rich honeycomb layered tellurate: Ag_6_ *M*_2_TeO_6_ (Ag atoms are drawn in blue, Te atoms in pink, Ni atoms in green and O atoms in red). a) A unit cell of Ag_6_ *M*_2_TeO_6_ showing the alignment of the atoms as viewed in the [100] direction. b) A perspective view of the unit cell of Ag_6_ *M*_2_TeO_6_ shows the alignment of the atoms. The Ag layers form two triangular lattices (drawn as continuous grey lines (up) or dashed grey lines (down)) comprising a single bilayer. The bilayers are separated a distance, *R* by a stabilizing energy gap, $K\propto \Delta \left(d\right)/R^{2}$ arising from SU(2)× U(1) spontaneous symmetry breaking (finite $\mathrm{Ag}-{\mathrm{Ag}}^{\prime }$ argentophilic interactions, Note S2, Supporting Information). c) The honeycomb bilayer lattice of Ag atoms in Ag_6_ *M*_2_TeO_6_ drawn as red lines as viewed from the [001] direction, showing the honeycomb unit cell (the transparent gray rhombus with unit vectors drawn as black and red arrows) and the triangular lattices described in (b). Each Ag atom in the honeycomb unit cell is assigned a pseudo‐spin up or down (drawn as green arrows) with opposite orientation to reflect the *S* transformation on the honeycomb lattice, hence introducing two chiral states of Ag in the honeycomb lattice, Λ. d) The topology of the honeycomb unit cell (flat torus, $\Lambda \in R^{2}$) depicted in (c) as a torus. The opposite ends of the unit cell are identified with each other, reflecting the translation symmetries (*T* transformations) of the honeycomb lattice, hence relating the honeycomb lattice to the two‐torus (genus one 2D surface, $T^{2}$). Our model requires the finite curvature introduced in the direction of the red arrows to be equivalent to a phase transition from 2D to 3D (Δ(d)=(d−2)/2, d=2,3) due to $\mathrm{Ag}-{\mathrm{Ag}}^{\prime }$ argentophilic interactions, which is responsible for the energy gap (and hence the bilayers) encountered in (a) and (b).

From a pedagogical perspective, the honeycomb‐layered Ag_2_ *M*_2_TeO_6_ with Ag‐rich bilayered domains (**Figure** **4**a) present a prolific playground to investigate the physical origins of argentophilic interactions in such frameworks. Although the Ag sub‐valent state is considered integral in the formation of stable bilayers, no apparent mechanism consociating sub‐valency to the presence of bilayers has been availed in literature hitherto. Thus, it is prudent to investigate the selection mechanism for the bilayer arrangement supplanting the single layers in other conventional layered materials. Notably, the triangular lattice observed in the Ag bilayers (such as in Figure 4b,c) can theoretically be understood as the general manifestation of the underlying emergent geometric field theories associated with the crystalline parameters favored by the Ag atoms as shown in Figure 4d.

In particular, we consider the crucial features in such bilayered frameworks to be: i) the unconventional $\mathrm{Ag}-{\mathrm{Ag}}^{\prime }$ bonding between like charges, whose nature can be interpreted as the already reported argentophilic interaction,^[^ ^89^, ^90^ ^]^ ii) the existence of Ag sub‐valent states in almost all reported bilayered frameworks,^[^ ^31^, ^33^, ^34^, ^35^, ^36^, ^41^, ^64^, ^76^, ^91^ ^]^ and iii) the apparent bifurcated bipartite honeycomb lattice. Fairly recently, an idealized model of cations describing the diffusion of cations in monolayered frameworks was formulated, whereby the number of vacancies created by diffusing cations can be related to the Gaussian curvature by the Poincaré–Hopf theorem for an emergent geometric theory consistent with Liouville conformal field theory (2D quantum gravity).^[^ ^1^, ^10^, ^12^, ^13^, ^16^ ^]^ It is thus imperative to also reproduce the conclusions of the idealized model along the way, which not only classifies the symmetries of the hexagonal and honeycomb cationic lattices in applicable honeycomb layered oxides but also the topological diffusion aspects in 2D. Indeed, this has been achieved by the theoretical model below.

### 3.3 Theoretical Model for Bilayered Honeycomb Frameworks

We shall set Planck's constant, the speed of electromagnetic waves in the material and Boltzmann's constant to unity (respectively, $\hbar =\bar{c}=k_{B}=1$) and employ Einstein summation convention for all raised and lowered indices unless explicitly stated otherwise.

Due to the electrostatic shielding of electric charge of the nucleus and other factors, electron occupation of orbital energy levels for transition metals can disobey Aufbau principle typically employed in standard chemistry to determine electronic configurations of atoms and their valencies.^[^ ^92^ ^]^ For group 11 elements, the $nd^{10}$ and (n+1)s orbitals are at close proximity (<3.5 eV) to each other^[^ ^93^ ^]^, sd hybridization is plausible, and can be responsible for degenerate states. In the case of the neutral Ag atom, the electronic configuration can either be $4d^{10}5s^{1}$ (labeled as ${\mathrm{Ag}}_{+1/2}$) which yields oxidation state, $4d^{10}5s^{0}$ given by ${\mathrm{Ag}}^{1+}$, or $4d^{9}5s^{2}$ (labeled as ${\mathrm{Ag}}_{-1/2}$) that yields oxidation state $4d^{10}5s^{2}$ given by ${\mathrm{Ag}}^{1-}$, whereby the superscript on Ag denotes the oxidation/valency state (i.e., number of electrons that can readily be lost to achieve stability in a chemical reaction) and the subscript ±1/2 is a spin degree of freedom transforming under SU(2) gauge group known as isospin.^[^ ^94^ ^]^ It is clear that, due to sd hybridization, $4d^{9}5s^{2}$ and $4d^{10}5s^{1}$ will be degenerate. Moreover, due to the doubly occupied *s* orbital of $4d^{9}5s^{2}$, another oxidation state, $4d^{9}5s^{0}$ exits, given by ${\mathrm{Ag}}_{0}\to {\mathrm{Ag}}^{2+}$, implying that the neutral Ag atom is three‐fold degenerate. Note that, ${\mathrm{Ag}}_{-1/2}$ and ${\mathrm{Ag}}_{0}$ are degenerate with essentially the same electronic state, $4d^{9}5s^{2}$, *albeit* different predisposition to form oxidation states ${\mathrm{Ag}}^{1-}$ and ${\mathrm{Ag}}^{2+}$ respectively. Evidently, such degeneracies must be lifted by introducing symmetry breaking in order to create the appropriate oxidation states stabilizing argentophilic bonds in layered materials. In particular, honeycomb layered materials with a monolayered structure tend to either have prismatic or linear coordinations of Ag to oxygen atoms,^[^ ^25^ ^]^ which should result in crystal field splitting of the 4*d* orbitals, whereby $4d_{z^{2}}$ is the lowest energy level in prismatic coordinations (or the highest energy level in linear coordinations).^[^ ^95^, ^96^ ^]^

**Figure 5:**
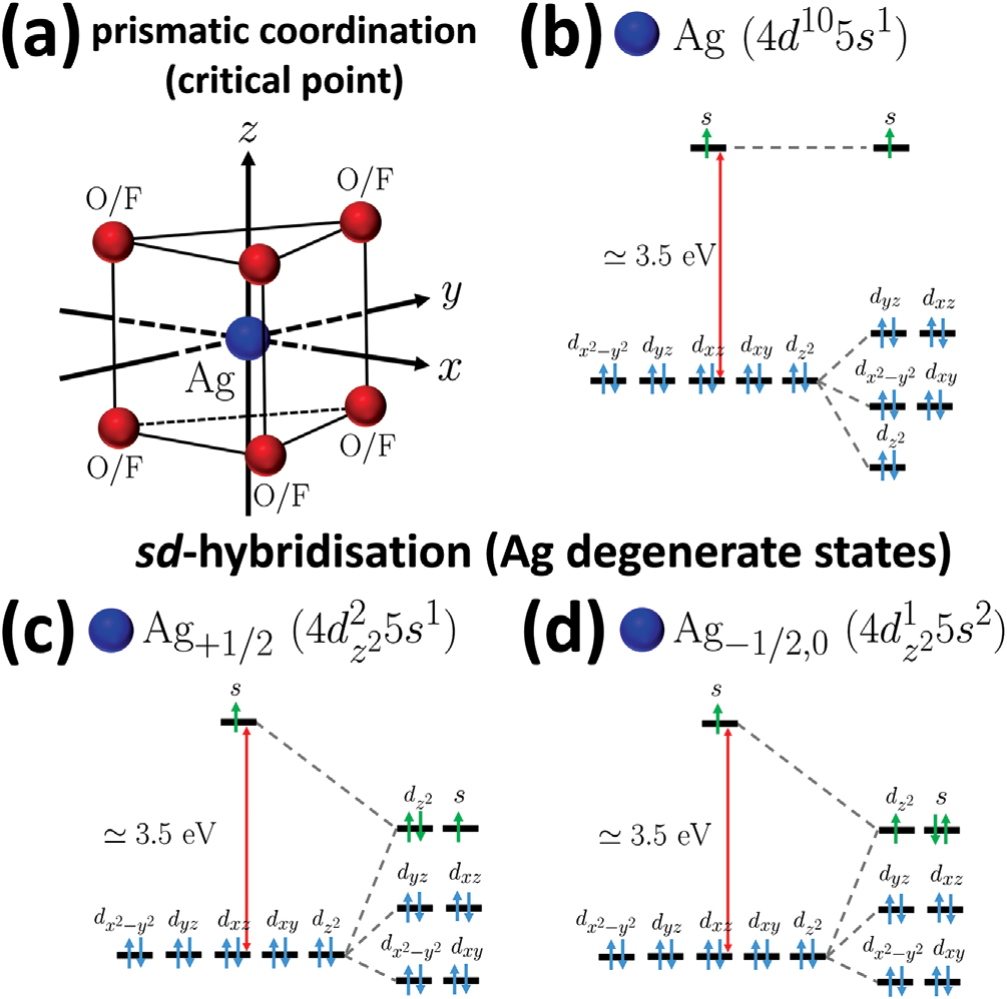
Example of crystal field splitting of Ag atom in a prismatic environment and the ensuing sd‐hybridization. a) An example of expected sd‐hybridization in silver, Ag atoms (blue) prismatically coordinated with F or O atoms (red) *e.g*., in the pre‐bifurcated silver lattice (critical point of the conformal field theory (CFT)) of ${\mathrm{Ag}}_{2}F$ or ${\mathrm{Ag}}_{2}{\mathrm{NiO}}_{2}$, respectively.^[^ ^64^, ^76^ ^]^ b–d) A schematic of the electron (spins), indicated by up or down arrows, occupying each 4*d* ^10^ and 5*s* orbital in the 3 degenerate states of Ag atoms labeled as ${\mathrm{Ag}}_{+1/2}$, ${\mathrm{Ag}}_{-1/2}$ and ${\mathrm{Ag}}_{0}$, whereby the core and valence electrons are shown as blue and green arrows, respectively. Neglecting crystal field splitting, all 4*d* ^10^ orbitals would have an equal probability to sd‐hybridize with the 5*s* orbital, which serves as an arena for other interesting mechanisms. The energy gap between the $4d_{z}$ and 5*s* orbitals has been taken to be ≈3.5 eV.^[^ ^93^ ^]^

A typical crystal field splitting of *d* orbitals in the prismatic environment^[^ ^97^ ^]^ is shown in **Figure** **5**a, whereby in our case assuming completely filled 4*d* ^10^ orbitals, $4d_{z^{2}}^{2}$ has the lowest energy, followed by degenerate $4d_{x^{2}-y^{2}}^{2}$ and $4d_{xy}^{2}$ orbitals and finally degenerate $4d_{yz}^{2}$ and $4d_{xz}^{2}$ orbitals with the highest energy, as shown in Figure 5b. After hybridization, the rest of the 4*d* orbitals are fully occupied with their energy levels unaltered, except for the newly formed Ag degenerate states, $4d_{z^{2}}^{2}5s^{1}$ (${\mathrm{Ag}}_{+1/2}$) and $4d_{z^{2}}^{1}5s^{2}$ (${\mathrm{Ag}}_{0},{\mathrm{Ag}}_{-1/2}$) given in Figure 5c,d, which are only distinguishable via their valency states. Similar considerations apply in the case of linear/dumbbell coordination, etc. Thus, neglecting crystal field splitting, all 4*d* ^10^ orbitals must have near‐equal probability to sd‐hybridize with the 5*s* orbital, which serves as an arena for other interesting mechanisms. Nonetheless, whilst this crystal field splitting is not a requisite for sd‐hybridization, such additional mechanisms on the honeycomb lattice require the isolated $4d_{z^{2}}$ orbital to play the role of $2p_{z}$ orbital in graphene, whereby the crystal field splitting of the 4*d* orbitals together with sd hybridization play a role analogous to $sp^{2}$ hybridization.^[^ ^98^ ^]^ Consequently, most properties such as pseudo‐spin, pseudo‐magnetic field, etc. exhibited by the itinerant $p_{z}^{1}$ electrons on graphene^[^ ^98^, ^99^, ^100^, ^101^ ^]^ can be mapped to the electron properties on the $4d_{z^{2}}^{1}$ electrons in Ag.^[^ ^10^ ^]^

Meanwhile, sd‐hybridization guarantees that the electron properties of all the $4d_{z^{2}}5s$ (valence) electrons can be associated with the Ag atoms themselves—a feature that is not expected in graphene‐based systems. In other words, the theory of such electrons in graphene is 1 + 2 dimensional quantum electrodynamics in a static space‐time background whereas the dynamical nature of the silver atoms (which can diffuse during intercalation/de‐intercalation processes) in contrast to carbon atoms in graphene lead instead to a dynamical space‐time (quantum gravity), whereby a bifurcated honeycomb lattice introduces 1 + 3 dimensions (same as Einstein gravity).^[^ ^10^ ^]^ Due to the complexity involved in the description of dynamical space‐times, we shall consider a static and flat space‐time background. Proceeding, we can summarize the oxidation states to be searched for experimentally by ${\mathrm{Ag}}^{1+}/{\mathrm{Ag}}_{+1/2}$, ${\mathrm{Ag}}^{1-}/{\mathrm{Ag}}_{-1/2}$, and ${\mathrm{Ag}}^{2+}/{\mathrm{Ag}}_{0}$, where ${\mathrm{Ag}}_{0}$ is also given by $4d^{9}5s^{2}$, reflecting its predisposition to form ${\mathrm{Ag}}^{2+}$ oxidation state instead of ${\mathrm{Ag}}^{1-}$. Indeed, ${\mathrm{Ag}}^{2+}$ has been experimentally observed in ${\mathrm{Ag}}^{2+}F_{2}^{1-}$ and its $4d_{z^{2}}^{1}5s^{0}$ character verified by its anti‐ferromagnetism arising from ${\mathrm{Ag}}^{2+}-F_{\left(2\right)}-{\mathrm{Ag}}^{2+}$ super‐exchange interactions,^[^ ^102^, ^103^ ^]^ whilst ${\mathrm{Ag}}^{1-}$ has been reported in coinage metal cluster ions such as ${\mathrm{Ag}}_{N}^{1-}$, where N=1,2,3, etc.^[^ ^104^, ^105^, ^106^, ^107^ ^]^

Nonetheless, we are interested in lifting these degeneracies by symmetry breaking. In nuclear physics, despite their different masses and charges, the proton and the neutron are essentially degenerate particles related by a degree of freedom that transforms appropriately under the SU(2) gauge group known as iso‐spin.^[^ ^94^ ^]^ This is also the case for leptons in the standard model of particle physics such as the electron and the neutrino, well described by SU(2)× U(1) symmetry breaking, where U(1) is the symmetry group also exhibited by Maxwell's equations.^[^ ^62^, ^63^ ^]^ In our present case, in order to ensure the degenerate states of Ag are treated as fermions, we are interested in ${\mathrm{Ag}}_{+1/2},{\mathrm{Ag}}_{-1/2}$, and ${\mathrm{Ag}}^{2+}$ since they have an odd number of electrons in their orbitals. Note that, the actual fermionic or bosonic character of these states will differ by including the spin of the protons and neutrons in Ag, reflecting the fact that we are only interested in the bonding properties of the degenerate states with dynamics inherited solely from the valence electrons. In our formalism, the degeneracy between ${\mathrm{Ag}}_{+1/2}$ and ${\mathrm{Ag}}_{-1/2}$ corresponds to isospin, which introduces the gauge group, SU(2). Moreover, the degeneracy between ${\mathrm{Ag}}^{2+}$ and ${\mathrm{Ag}}_{-1/2}$ can be treated on the honeycomb lattice as the spin degree of freedom known in graphene physics as pseudo‐spin.^[^ ^99^, ^100^ ^]^ To yield results consistent with experimental observations in bilayered frameworks, ${\mathrm{Ag}}_{+1/2}$ and ${\mathrm{Ag}}_{-1/2}$ have left‐handed chirality whereas ${\mathrm{Ag}}^{2+}$ is right‐handed.

By promoting/demoting an electron using a neutral gauge field, ${\mathrm{Ag}}_{+1/2}$ can transform into ${\mathrm{Ag}}_{-1/2}$ ($4d^{10}5s^{1}\to 4d^{9}5s^{2}$) and vice versa but never into ${\mathrm{Ag}}^{2+}$, since this is an oxidation state requiring the loss of electrons. We shall treat the entangled state of the electron pair and the neutral gauge field as a charged $W_{\pm }^{\mu }=\frac{1}{\sqrt{2}}\left(W_{1}^{\mu }\pm iW_{2}^{\mu }\right)$ gauge boson, responsible for this transition, where $W^{\mu }=W_{a}^{\mu }\tau_{a}$ is a gauge field transforming under SU(2) ($\tau_{a}=\sigma_{a}/2$, $\sigma_{a}=\left(\sigma_{1},\sigma_{2},\sigma_{3}\right)$ are the Pauli matrices). This transition has to be of the order ≈3.5 eV, corresponding to the mass of $W_{\pm }^{\mu }$. We shall also consider a screened and an un‐screened Coulomb potential, $Z_{\mu }$ and $p_{\mu }$, respectively, obtained by the mixing.

(1) $$\begin{array}{c} \left(\begin{array}{c} A^{\mu }\\ W_{3}^{\mu } \end{array}\right)=\frac{1}{\sqrt{q_{e}^{2}+q_{w}^{2}}}\left(\begin{array}{cc} q_{w} & -q_{e}\\ q_{e} & q_{w} \end{array}\right)\left(\begin{array}{c} p^{\mu }\\ Z^{\mu } \end{array}\right) \end{array}$$

where $q_{e}≅\;1.602\times 10^{-19}$ C is the elementary (U(1)) charge, $A_{\mu }$ is the electromagnetic field, *q*_w_ is the SU(2) charge, and

(2) $$\begin{array}{c} q_{\mathrm{eff}}=\frac{q_{w}q_{e}}{\sqrt{q_{w}^{2}+q_{e}^{2}}} \end{array}$$

is the effective coupling/charge to $p_{\mu }$. Now, introducing the charge operator *Y* for the U(1) electric charge the generator of the effective charge, *Q* for the field $p_{\mu }$ is given by (a variant of) the Gell–Mann–Nishijima relation,^[^ ^62^ ^]^

(3) $$\begin{array}{c} Q=2I+Y \end{array}$$

with $I=\tau_{3}=\sigma_{3}/2=\pm 1/2,0$ the emergent iso‐spins of left‐handed (${\mathrm{Ag}}_{+1/2}\left(I=+1/2\right)$, ${\mathrm{Ag}}_{-1/2}\left(I=-1/2\right)$) and right‐handed (${\mathrm{Ag}}^{2+}\left(I=0\right)$) fermions, respectively, corresponding to half the valency/oxidation state of the left‐handed Ag cations, ${\mathrm{Ag}}_{+1/2}\to {\mathrm{Ag}}^{1+},{\mathrm{Ag}}_{-1/2}\to {\mathrm{Ag}}^{1-}$ but vanishes for the right‐handed ${\mathrm{Ag}}^{2+}$ oxidation state. Meanwhile, since $W_{\pm }^{\mu }$ carries the iso‐spin by transforming ${\mathrm{Ag}}_{+1/2}$ into ${\mathrm{Ag}}_{-1/2}$ and vice versa, it has iso‐spin, I=±1, with an effective charge of Q=±1 and electric charge, Y=0. Thus, the effective charge of $W_{\pm }^{\mu }$ is a result of the entangled state with the valence electron, as earlier remarked. Moreover, charge and iso‐spin conservation for the interaction between $W_{\pm }^{\mu }$ and the left‐handed Ag states yields for ${\mathrm{Ag}}_{+1/2}$, Y=0, Q=+1 and for ${\mathrm{Ag}}_{-1/2}$, Y=0, Q=−1.

A summary of these charges and spins for the cations and the gauge bosons has been availed in **Table** **1**. The appropriate Lagrangian is given by,

(4) $$\begin{array}{c} L=\int d^{\;3}x\;\frac{1}{4}\left({\left|\partial_{\mu }{\vec{W}}_{\nu }+q_{w}\left({\vec{W}}_{\mu }\times {\vec{W}}_{\nu }\right)\right|}^{2}+{\left(\partial_{\mu }A_{\mu }-\partial_{\nu }A_{\mu }\right)}^{2}\right)\\ +\int d^{\;d}x\;\left({\bar{\psi }}_{L}\gamma^{\mu }\left(i\partial_{\mu }+q_{w}W_{\mu }+\frac{Y}{2}q_{e}A_{\mu }\right)\psi_{L}\right.\\ \left.+\;{\bar{\psi }}_{R}\gamma^{\mu }\left(i\partial_{\mu }+\frac{Y}{2}q_{e}A_{\mu }\right)\psi_{R}\right)\\ +\int d^{\;d}x\;\left(\frac{1}{2}{\left|\left(\partial_{\mu }+iq_{w}W_{\mu }+\frac{Y}{2}iq_{e}A_{\mu }\right)\phi \right|}^{2}\right.\\ \left.-\alpha {\left(v^{2}-\frac{1}{4}\left(\phi^{†}\phi \right)\right)}^{2}+\gamma {\bar{\psi }}_{R}\phi^{†}\psi_{L}+h.c.\right) \end{array}$$

where $\psi_{L}^{T}=\left({\mathrm{Ag}}_{+1/2},{\mathrm{Ag}}_{-1/2}\right)$, $\psi_{R}^{T}={\mathrm{Ag}}^{2+}$, ${\bar{\psi }}_{L/R}=\psi_{L/R}^{*T}\gamma^{0}$, and $\phi^{†}=(0,\Psi^{*}|T_{c}{|}^{-1/2})$; the superscript T is the transpose, h.c. stands for Hermitian conjugate (of $\gamma {\bar{\psi }}_{R}\phi^{†}\psi_{L}$), $\gamma^{\mu }$ are the gamma matrices, α, *v*, γ are constants, and ${\left|\Psi \right|}^{2}$ is a *d*D condensate satisfying^[^ ^10^ ^]^

(5) $$\begin{array}{c} \int d^{\;d}x\;{\left|\Psi \right|}^{2}=2k,\;\;\left\langle \left\langle k\right\rangle \right\rangle +\Delta \left(d\right)=0 \end{array}$$

where k∈N is the number of cationic vacancies^[^ ^1^, ^12^, ^13^ ^]^ behaving like a condensate, Δ(d)=(d−2)/2 is the scaling dimension of a mass‐less scalar conformal field theory^[^ ^10^, ^108^ ^]^ and d=2,3 with a suitable average ⟨⟨⋯⟩⟩ corresponding to a normalized Mellin transform^[^ ^10^ ^]^ of the thermal average $\left\langle k\right\rangle =\left\langle a^{†}a\right\rangle$ (Here, $a^{†},a$ are the quantum harmonic oscillator raising, lowering operators).

**Table 1:** Comparison between the various charges and spins of the relevant cations and fields in the theory of SU(2)× U(1) spontaneous symmetry breaking in Ag bilayered materials

cation, field ∖ spin, charge	spin	iso‐spin	U(1) charge	effective charge	pseudo‐spin
	[*s*]	[*I*]	[*Y*]	[Q=2I+Y]	[$s^{\prime }$]
${\mathrm{Ag}}^{1+}/{\mathrm{Ag}}_{+1/2}$	±1/2	+1/2	0	+1	0
${\mathrm{Ag}}^{1-}/{\mathrm{Ag}}_{-1/2}$	±1/2	−1/2	0	−1	∓1/2
${\mathrm{Ag}}^{2+}/{\mathrm{Ag}}_{0}$	±1/2	0	+2	+2	±1/2
$p^{\mu }$	±1	0	0	0	0
$W_{\pm }^{\mu }$	±1	±1	0	±1	0
$Z^{\mu }$	±1	0	0	0	0
$\Psi ,\Psi^{*}$	0	−1/2,+1/2	−2,+2	−3,+3	∓1,±1

The desired field theory results can be computed in parallel to electroweak theory (specifically, the lepton interactions in the standard model,^[^ ^62^, ^63^ ^]^ with the exception of Y=0 for the left‐handed cations, as in Table 1, instead of Y=−1 for the leptons in standard model, which leaves ${\mathrm{Ag}}^{1+}$ charged (Q≠0) unlike the neutrino (Q=0)) *albeit* in 1+d dimensions (d=2,3) with the electromagnetic field, $A_{\mu }$ playing the role of the hyper‐charge.^[^ ^62^ ^]^ Focusing only on the important features, the Lagrangian introduces a mass for $W_{\pm }^{\mu }$ and $Z^{\mu }$ ($m_{W}$ and $m_{Z}$, respectively) related by

(6) $$\begin{array}{c} 3.5\;\mathrm{eV}\approx m_{W}=\frac{q_{w}}{\sqrt{q_{e}^{2}+q_{w}^{2}}}m_{Z} \end{array}$$

for |Ψ|∝Δ(d)≠0. Recall that $1/m_{Z}$ is the screening length of electromagnetic interactions within Ag bilayers. Thus, to determine *q*_w_, one can use eq. (2), where *q*_eff_ is the effective charge of the cations as measured in experiments with bilayers (which is expected to differ from $q_{e}=1.602\times 10^{-19}$ C), then proceed to solve for *q*_w_, which yields the mass $m_{Z}$ using Equation (6). Moreover, only $\psi^{T}=\left({\mathrm{Ag}}^{2+},{\mathrm{Ag}}_{-1/2}\right)$ acquires a mass term (potential/bonding energy).

(7) $$\begin{array}{ccc} U(\Delta ,T_{c}) & = & L_{\mathrm{mass}}\left(\bar{\psi },\psi \right)=\frac{\gamma }{|T_{c}{|}^{1/2}}\int d^{\;d}x\;\left|\Psi \right|\bar{\psi }\psi \\ & & ∼\frac{2\Delta (d)}{|T_{c}{|}^{\Delta^{*}\left(d\right)}}\int d^{\;d}x\;\left(T-T_{c}\right)\bar{\psi }\psi \leq 0 \end{array}$$

that we shall interpret as the origin of the argentophilic interaction between ${\mathrm{Ag}}^{2+}$ and ${\mathrm{Ag}}^{1-}$, where $\Delta^{*}\left(d\right)=\left(1-2\Delta \left(d\right)\right)/2$, we have made the replacement, $\Psi^{*}\to \left|\Psi \right|\exp\left(-iS\right)$ with S=0 in $\phi^{†}$ for simplicity, and we have introduced the critical exponent

(8) $$\begin{array}{c} \left|\Psi \left(T,d\right)|=2v\left(T,d\right)\right|T_{c}{|}^{1/2}∼2\Delta \left(d\right){\left|T_{c}\right|}^{\Delta (d)}\left(T-T_{c}\right)/\gamma \geq 0 \end{array}$$

with v(T,d)=v≥0 the constant appearing in the Lagrangian (related to |Ψ| by minimizing the Mexican hat potential with respect to $\phi ,\phi^{†}$), *T* the temperature and *T*_c_ the critical temperature (acquired mass of ψ in d=3).^[^ ^109^ ^]^ Note that, the exponent Δ(d) appearing in $|T_{c}|$ is justified by dimensional analysis. Moreover, the mass of the fields $\phi ,\phi^{†}$ corresponds to $m_{\phi }=\left|\alpha \right|v^{2}$. Consequently, all mass terms vanish in d=2 dimensions due to the scaling dimension, Δ(d=2)=0, but are finite for d=3 provided $T>T_{c}$, since Δ(d=3)=1/2. This represents a monolayer‐bilayer phase transition for $T>T_{c}$, where $T(\vec{x})$ is a temperature field that behaves like the Higgs field.^[^ ^62^ ^]^ Moreover, its bosons can be interpreted as phonons within the material arising from high temperature dynamics.

The triumph herein is that the formalism satisfies (i), (ii), and (iii) above. Specifically, (ii) is satisfied for instance by writing,^[^ ^2^ ^]^ ${\mathrm{Ag}}_{2}^{1/2+}{\mathrm{Ni}}^{3+}O_{2}^{2-}={\mathrm{Ag}}^{2+}{\mathrm{Ag}}^{1-}{\mathrm{Ni}}^{3+}O_{2}^{2-}$. Other sub‐valent states such as ${\mathrm{Ag}}_{3}^{2/3+}{\mathrm{Ni}}_{2}^{3+}O_{4}^{2-}$ represent a saturation or hybrid effect by the mass‐less ${\mathrm{Ag}}^{1+}$ fermion,^[^ ^2^ ^]^

(9a) $$\begin{array}{ccc} & & {\mathrm{Ag}}^{2+}{\mathrm{Ag}}^{1-}{\mathrm{Ni}}^{3+}O_{2}^{2-}+{\mathrm{Ag}}^{1+}{\mathrm{Ni}}^{3+}O_{2}^{2-}\\ & & \;\to Ag^{2+}Ag^{1-}Ag^{1+}Ni_{2}^{3+}O_{4}^{2-}=Ag_{3}^{2/3+}Ni_{2}^{3+}O_{4}^{2-} \end{array}$$

Lastly, ${\mathrm{Ag}}^{1+}{\mathrm{Ni}}^{3+}O_{2}^{2-}$ cannot be bilayered since ${\mathrm{Ag}}^{1+}$ is mass‐less in the theory. In the case of the present material in this study, assuming ${\mathrm{Te}}^{6+}$, the under‐saturated bilayered material is expected to be given by ${{\mathrm{Ag}}_{2}^{2+}{\mathrm{Ag}}_{2}^{1-}\mathrm{Ni}}_{2}^{2+}{\mathrm{Te}}^{6+}O_{6}^{2-}={\mathrm{Ag}}_{4}^{1/2+}{\mathrm{Ni}}_{2}^{2+}{\mathrm{Te}}^{6+}O_{6}^{2-}$ or ${{\mathrm{Ag}}_{4}^{2+}{\mathrm{Ag}}_{4}^{1-}\mathrm{Ni}}_{2}^{2+}{\mathrm{Te}}^{4+}O_{6}^{2-}={\mathrm{Ag}}_{8}^{1/2+}{\mathrm{Ni}}_{2}^{2+}{\mathrm{Te}}^{4+}O_{6}^{2-}$ (Ag coordination to O is assumed prismatic) with Ag sub‐valency +1/2, consistent with our XPS and XAS experimental observations (${\mathrm{Ni}}^{2+},{\mathrm{Te}}^{4+},{\mathrm{Te}}^{6+}$). However, the STEM‐EDX spectra results require the chemical formula, ${\mathrm{Ag}}_{6}{\mathrm{Ni}}_{2}{\mathrm{TeO}}_{6}$ for the Ag‐rich domain bilayered domains of the present material. Moreover, we can have, ${{\mathrm{Ag}}_{4}^{1+}{\mathrm{Ag}}_{2}^{1-}\mathrm{Ni}}_{2}^{2+}{\mathrm{Te}}^{6+}O_{6}^{2-}={\mathrm{Ag}}_{6}^{1/3+}{\mathrm{Ni}}_{2}^{2+}{\mathrm{Te}}^{6+}O_{6}^{2-}$ or ${{\mathrm{Ag}}_{2}^{2+}{\mathrm{Ag}}_{2}^{1-}{\mathrm{Ag}}_{2}^{1+}\mathrm{Ni}}_{2}^{2+}{\mathrm{Te}}^{4+}O_{6}^{2-}={\mathrm{Ag}}_{6}^{2/3+}{\mathrm{Ni}}_{2}^{2+}{\mathrm{Te}}^{4+}O_{6}^{2-}$, where the former can be excluded theoretically from the bilayered frameworks since it lacks the right‐handed, ${\mathrm{Ag}}^{2+}$ valency state and hence lacks the mass term responsible for the bilayered structure. Whilst not a requisite in the theory, we note that none of these materials contain both ${\mathrm{Te}}^{4+}$ and ${\mathrm{Te}}^{6+}$ as observed experimentally. To remedy this, we shall consider the hybrid,

(9b) $$\begin{array}{c} {{\mathrm{Ag}}_{2}^{2+}{\mathrm{Ag}}_{2}^{1-}\mathrm{Ni}}_{2}^{2+}{\mathrm{Te}}^{6+}{O_{6}^{2-}+{\mathrm{Ag}}_{4}^{2+}{\mathrm{Ag}}_{4}^{1-}\mathrm{Ni}}_{2}^{2+}{\mathrm{Te}}^{4+}O_{6}^{2-}\to \\ {\mathrm{Ag}}_{6}^{2+}{\mathrm{Ag}}_{6}^{1-}{\mathrm{Ni}}_{4}^{2+}{\mathrm{Te}}^{4+}{\mathrm{Te}}^{6+}O_{12}^{2-}=2{\mathrm{Ag}}_{6}^{1/2+}{\mathrm{Ni}}_{2}^{2+}{\mathrm{Te}}^{5+}O_{6}^{2-}, \end{array}$$

which is also bilayered, consistent with the theory. Ideally, the oxidation states ${\mathrm{Ag}}^{2+}$, ${\mathrm{Ag}}^{1-}$, and ${\mathrm{Ag}}^{1+}$ alongside the sub‐valency states of ${\mathrm{Ag}}^{1/2+}$ or ${\mathrm{Ag}}^{2/3+}$, when present, should be observable in XPS or XAS data, provided distinguishability issues encountered such as the crystalline homogeneity (phase purity) of the as‐prepared material encountered in the present work can be adequately addressed. Moreover, the energy gap of ≈ 3.5 eV should be existent in measurement data (XPS or XAS) whenever ${\mathrm{Ag}}^{1+}/{\mathrm{Ag}}_{+1/2}$ is available and transmutes into ${\mathrm{Ag}}^{1-}/{\mathrm{Ag}}_{-1/2}$ or vice versa. This should occur, for instance, in processes where a bilayered structure disintegrates into a monolayered structure (or vice versa) by the emission or absorption of photo‐electrons of order energy gap between the 5*s* and $4d_{z}$ orbitals, taken to be ≈3.5 eV. Presently, we have neither tested nor observed such a monolayer‐bilayer phase transition, which falls beyond the technical abilities brought to bear in the present work. For brevity, other considerations for additional sub‐valent states not fully consistent with our observations in this paper will be considered elsewhere.^[^ ^2^, ^10^ ^]^

Finally, topological aspects consistent with idealized model^[^ ^1^, ^12^, ^13^ ^]^ can be calculated in d=2 dimensions by ^[^ ^62^ ^]^

(10a) $$\begin{array}{c} \int D\left[\bar{\psi },\psi \right]\exp\left(i\int dt\;L\right)=\exp\left(iS^{\prime }\right)\exp\left(iS_{\mathrm{CS}}\right) \end{array}$$

(10b) $$\begin{array}{c} S_{\mathrm{CS}}=\frac{q_{\mathrm{eff}}^{2}}{2}\int dt\int d^{\;2}x\;\left(\frac{1}{4\pi }\frac{T_{c}}{|T_{c}|}\varepsilon^{\mu \nu \sigma }p_{\mu }\partial_{\nu }p_{\sigma }+\phi^{†}\phi \;p_{\mu }p^{\mu }\right) \end{array}$$

where $\varepsilon^{\mu \nu \sigma }$ is the Levi–Civita symbol normalized as $\varepsilon^{012}=1$, and we have kept leading terms with $p_{\mu }$ in *S*_CS_ (Chern–Simons action^[^ ^62^, ^110^ ^]^) and the rest in $S^{\prime }$. The equations of motion yield

(11) $$\begin{array}{c} \frac{1}{4\pi }\varepsilon^{\mu \nu \sigma }\partial_{\nu }p_{\sigma }=T_{c}^{-1}{\left|\Psi \right|}^{2}p^{\mu } \end{array}$$

where we have used $\phi^{†}\phi ={\left|\Psi \right|}^{2}/\left|T_{c}\right|$. Moreover, we can rescale $p_{\mu }\to p_{\mu }\sqrt{2}/q_{\mathrm{eff}}$ and set $\vec{p}=\vec{n}\times \vec{\nabla }\Phi$ where $p^{\mu }=\left(T_{c},\vec{p}\right)$, $\vec{n}=\left(0,0,1\right)$ is the unit normal vector to the cationic honeycomb lattice, and Φ is the Liouville field satisfying, $\nabla^{2}\Phi =-K\exp\left(2\Phi \right)$ with *K* the Gaussian curvature.^[^ ^12^, ^13^ ^]^ Thus, the time component of Equation (11) corresponds to, $\vec{\nabla }\times \vec{p}=\nabla^{2}\Phi =-K\exp\left(2\Phi \right)={\left|\Psi \right|}^{2}$, which satisfies Poincaré–Hopf theorem.^[^ ^13^ ^]^ There are two geometries of the Ag lattice representing the honeycomb lattice that satisfy the no vacancy condition, ∫Kexp(2Φ)=0: The flat torus (K=0) and the two‐torus $\int_{T^{2}}K\exp\left(2\Phi \right)=2\pi \left(2-2g\right)=-4\pi k=0$ (*g* corresponds to the genus of an emergent 2D surface without boundary) with K≠0, related to each other by the transformation given in Figure 4d. This means that the critical point is two‐fold degenerate. Coordinate transformations in this state correspond to conformal invariance (modular invariance^[^ ^13^ ^]^), which is broken for g≠1. This can be achieved either by the creation of vacancies g>1 or by the system lowering its ground state energy by exploiting the additional g=0 state. This additional state is the energetically more stable configuration away from the critical point ($T>T_{c}$), *albeit* inaccessible in d=2 dimensions. In other words, the bipartite honeycomb lattice lifts this degeneracy by bifurcation—interpreted as a variant of the theorem of Peierls (1D) or Jahn–Teller (3D).^[^ ^10^, ^111^, ^112^ ^]^

Moreover, the opposite convention for the temperature in the phase transition (transition happens for $T>T_{c}$ instead of the conventional $T<T_{c}$) is indicative of the emergence of a 3D gravitational description of the potential in Equation (7)

(12) $$\begin{array}{c} U\left(\Delta ,T_{c}\right)≃-A\left(\Delta ,T_{c}\right)/R^{2}+B\left(\Delta ,T_{c}\right)/R^{3} \end{array}$$

where $A(\Delta ,T_{c})$, $B(\Delta ,T_{c})$ are constants independent of *R* and $R_{\mathrm{disp}.}=3B/2A≅2.8$ Å   is the observed displacement due to the bifurcation satisfying the condition, ${\partial U/\partial R|}_{R=R_{\mathrm{disp}.}}=0$, which scales as Newtonian acceleration/gravity.^[^ ^10^ ^]^ This can be interpreted as a dual description of the Liouville conformal field theory (CFT) vacancy states (k>1) in the spirit of gravity‐CFT duality.^[^ ^10^ ^]^ Finally, this potential corresponds to a Hamiltonian of the honeycomb lattice pseudo‐spin degrees of freedom (a 1D Ising model of pseudo‐spins interacting with a pseudo‐magnetic field corresponding to the Gaussian curvature (g=0) and the Heisenberg coupling taken to be the Ruderman–Kittel–Kasuya–Yosida (RKKY) interaction).^[^ ^13^ ^]^ Further discussion on the conformal field theoretic nature of the monolayer‐bilayer phase transition has been availed in Note S2 (Supporting Information).

## 4 Conclusion

We report the topochemical synthesis at 250 °C (and under ambient pressure) of a new honeycomb layered oxide with a global average composition of Ag_2_ *M*_2_TeO_6_ (M=Ni,Mg,etc.), manifesting Ag‐rich and Ag‐deficient domains. Aberration‐corrected transmission electron microscopy reveals the Ag‐rich crystalline domains with a composition of Ag_6_ *M*_2_TeO_6_ to exhibit Ag atom bilayers with aperiodic stacking disorders. X‐ray absorption spectroscopy and X‐ray photoelectron spectroscopy ascertain sub‐valent Ag states innate in the bilayer Ag_6_ *M*_2_TeO_6_ domains with the origin rationalized to arise from spontaneous symmetry breaking of degenerate mass‐less chiral fermion states of silver. Such a phase transition yields three oxidation states of silver (${\mathrm{Ag}}^{1+}$, ${\mathrm{Ag}}^{1-}$, and ${\mathrm{Ag}}^{2+}$) on the honeycomb lattice, based on the occupancy of their $4d_{z^{2}}$ and 5*s* orbitals, and a mass term between ${\mathrm{Ag}}^{2+}$ and ${\mathrm{Ag}}^{1-}$ responsible for the bilayered structure. From this theoretical point of view, we acquire an intuitive picture for the origin of the argentophilic bond between Ag pairs responsible for stabilizing the bilayers in Ag‐based materials with matching characteristics.^[^ ^30^, ^31^, ^32^, ^33^, ^34^, ^35^, ^36^, ^41^ ^]^ Moreover, since the 3.5 eV is the energy cost of sd‐hybridization that leads to degeneracy of the valence band (5*s*‐orbitals) and conduction bands ($4d_{z^{2}}$‐orbitals) on the honeycomb lattice, lifting this degeneracy corresponds to a metal–semiconductor/metal–insulator phase transition, in the spirit of Peierls instability^[^ ^111^ ^]^ (analogous to Cooper‐pair instability, which is related to paired electrons), resulting in an energy gap.^[^ ^113^ ^]^ Whilst this energy gap will differ from the 3.5 eV depending on the nature of the instability, in the case of Ag‐bilayered materials, the bilayered structure ought to be able to disintegrate into a monolayered structure and vice versa by the emission or absorption of photoelectrons of order energy gap between the $4d_{z^{2}}$ and 5*s* orbitals, taken to be ≈3.5 eV.^[^ ^93^ ^]^ Presently, we have neither tested nor observed such a monolayer‐bilayer (conductor–semiconductor/insulator) phase transition, or related effects which falls beyond the technical abilities brought to bear in the present work.

Nonetheless, the achieved experimental and theoretical insights not only promise to augment the literature space of Ag‐based honeycomb‐layered oxides structures, mechanisms, and functionalities, but also are poised to inspire innovative applications for these next‐generation functional materials. Ultimately, we regard the silver‐based honeycomb‐layered tellurate as a pedagogical platform for further inquiry into the role of geometric features and non‐commutative electromagnetic interactions, which go beyond energy storage applications.

## 5 Experimental Section

### Synthesis of Materials

Honeycomb‐layered oxides embodying the global composition of Ag_2_ *M*_2_TeO_6_ (where M=Ni,Co,Cu,Zn,Mg, and ${\mathrm{Ni}}_{0.5}{\mathrm{Co}}_{0.5}$) were synthesized via the topochemical ion‐exchange of Na_2_ *M*_2_TeO_6_ precursors alongside a molten flux of ${\mathrm{AgNO}}_{3}$ at 250 °C for 99 h in air, based on the following reaction:

(13) $$\begin{array}{ccc} & & \;{\mathrm{Na}}_{2}M_{2}{\mathrm{TeO}}_{6}\left(M=\mathrm{Ni},\mathrm{Mg},\mathrm{Co},\mathrm{Cu},\mathrm{Zn}\right)+2\;{\mathrm{AgNO}}_{3}\\ & & \;\to {\mathrm{Ag}}_{2}M_{2}{\mathrm{TeO}}_{6}+2\;{\mathrm{NaNO}}_{3} \end{array}$$

A fourfold molar excess amount of ${\mathrm{AgNO}}_{3}$ was used to facilitate a complete ion‐exchange reaction. To dissolve the residual nitrates (i.e., ${\mathrm{NaNO}}_{3}$ byproduct and remaining ${\mathrm{AgNO}}_{3}$), the resulting product was thoroughly washed with distilled water, vigorously stirred with a magnetic mixer and thereafter filtered and dried. The resulting Ag_2_ *M*_2_TeO_6_ displayed varied colours distinct from the precursor materials confirming the completion of the ion‐exchange reaction (see Figure S32, Supporting Information). Note that, $K_{2}M_{2}{\mathrm{TeO}}_{6}$ or $\mathrm{NaK}M_{2}{\mathrm{TeO}}_{6}$ can also be used as precursor materials. However, due to the inherent hygroscopicity of potassium‐based honeycomb‐layered compounds, Na_2_ *M*_2_TeO_6_ was selected as the precursors for a facile and scalable ${\mathrm{Na}}^{+}/{\mathrm{Ag}}^{+}$ ion‐exchange reaction. Na_2_ *M*_2_TeO_6_ precursor materials were prepared using the high‐temperature solid‐state reaction route detailed in literature.^[^ ^19^, ^20^, ^114^, ^115^, ^116^ ^]^

### X‐Ray Diffraction (XRD) Analyses

Conventional XRD (CXRD) measurements were conducted using a Bruker D8 ADVANCE diffractometer to ascertain the crystallinity of the as‐prepared Ag_2_ *M*_2_TeO_6_ powder samples. Measurements were performed in Bragg–Brentano geometry mode with Cu‐*K*α radiation. Synchrotron XRD (SXRD) measurements were performed to acquire high‐resolution data of as‐prepared Ag_2_ *M*_2_TeO_6_. SXRD experiments were performed at BL8S2 of Aichi SR Center. XRD ex situ measurements of pristine and charged ${\mathrm{Ag}}_{2-x}{\mathrm{Ni}}_{2}{\mathrm{TeO}}_{6}$ and ${\mathrm{Ag}}_{2-x}{\mathrm{NiCoTeO}}_{6}$ electrodes were collected in Bragg–Brentano geometry using a Cu‐*K*α monochromator. Prior to performing XRD measurements, the electrodes were thoroughly washed using super‐dehydrated acetonitrile and subsequently dried in an argon‐purged glove box.

### Morphological and Physicochemical Characterisation

Field emission scanning electron microscope (JSM‐7900F) was used to analyze the morphologies of the obtained powder samples. Energy dispersive X‐ray (EDX) imaging technique was used to assess the constituent elements of the obtained powders. Quantitative assessment of the chemical compositions was performed using inductively coupled plasma absorption electron spectroscopy (ICP‐AES). Further, the density of the as‐prepared powders was determined via pycnometric measurements (see Table S2, Supporting Information). The information obtained was used to calculate the pellet compactness prior to the electrochemical impedance spectroscopic measurements. Specimens for atomic‐resolution transmission electron microscopy (TEM) were prepared by an Ar‐ion milling method using a GATAN PIPS (Model 691) precision ion‐milling machine after embedding them in epoxy glue under an Ar atmosphere. High‐resolution scanning TEM (STEM) imaging and electron diffraction patterns were obtained using a JEOL JEM‐ARM200F incorporated with a CEOS CESCOR STEM Cs corrector (spherical aberration corrector). The acceleration voltage was set at 200 kV. Electron microscopy measurements were conducted along various zone axes (namely, [100], [010], and [310] zone axes). To mitigate beam damage to the samples, a low electron‐beam dosage (STEM probe current value of 23 pA) was used with short‐exposure times. The probe‐forming convergence angle was 22 mrad. High‐angle annular dark‐field (HAADF) and annular bright‐field (ABF) STEM snapshots were taken simultaneously at nominal collection angles of 90−370 mrad and 11−23 mrad, respectively. To reduce the possibility of image distortion induced by the specimen drift during the scan, a quick sequential acquisition technique was conducted to observe the atomic structures. It is important to mention here that images of the ${\mathrm{Ag}}_{2}{\mathrm{Zn}}_{2}{\mathrm{TeO}}_{6}$ could not be obtained on account of its low crystallinity (amorphous nature), as was further affirmed by XRD measurements. Attempts to improve crystallinity of the sample by annealing at temperatures below the decomposition regime proved elusive. For accurate localization of metal atoms in the obtained STEM maps, about 20 STEM images were recorded sequentially with an acquisition time of ≈0.5 s per image, after which the images were aligned and superimposed into one image. STEM‐EDX (energy‐dispersive X‐ray spectroscopy) spectrum images were obtained with two JEOL JED 2300T SDD‐type detectors with 100 ${\mathrm{mm}}^{2}$ detecting area whose total detection solid angle was 1.6 sr. Elemental maps were extracted using Thermo Fisher Scientific Noran (NSS) X‐ray analyzer. Reproducibility measurements on various crystallites were also performed using TITAN cubed G2 60‐300 TEM (FEI Company) (acceleration voltage: 300 kV) coupled with an EDX, in which the EDX measurements were conducted by using Super‐X (Bruker).

### Electrochemical Measurements

Fabrication of the composite electrode was performed by mixing the as‐prepared ${\mathrm{Ag}}_{2}{\mathrm{Ni}}_{2}{\mathrm{TeO}}_{6}$ and ${\mathrm{Ag}}_{2}{\mathrm{NiCoTeO}}_{6}$ polycrystalline powders with polyvinylidene fluoride (PVdF) binder and acetylene black (conductive carbon) at a weight ratio of 70:15:15. The mixture was suspended in *N*‐methyl‐2‐pyrrolidinone (NMP) to attain viscous slurry samples, which were then cast on aluminium foil with a typical mass loading of ≈5 mg cm^−2^, before drying under vacuum. Electrochemical measurements were assessed using CR2032‐type coin cells using ${\mathrm{Ag}}_{2}{\mathrm{Ni}}_{2}{\mathrm{TeO}}_{6}$ and ${\mathrm{Ag}}_{2}{\mathrm{NiCoTeO}}_{6}$ composite electrodes as the cathodes (working electrodes) in Ag half‐cells and Li half‐cells. Glass fibre discs were used as separators alongside electrolytes consisting of 0.1 mol dm^−3^ silver bis(trifluoromethanesulphonyl)imide (AgTFSI) in 1‐methyl‐1‐propylpyrrolidinium bis(trifluoromethanesulphonyl)imide (${\mathrm{Pyr}}_{13}\mathrm{TFSI}$) for the Ag half‐cells and 0.5 mol dm^−3^ lithium bis(trifluoromethanesulphonyl)imide (LiTFSI) in ${\mathrm{Pyr}}_{13}\mathrm{TFSI}$ ionic liquid as electrolyte for the Li half‐cells. The coin cells were assembled in an Ar‐filled glove box (MIWA, MDB‐1KP‐0 type) with oxygen and water contents maintained below 1 ppm. All electrochemical measurements were performed at room temperature. Galvanostatic cycling was done at a current rate commensurate to C/10 (10 being the necessary hours to charge to full theoretical capacity). The upper cut‐off voltage was set at 1.5 V for the Ag half‐cells, or 4.8 V as for the Li half‐cells.

### Thermal Stability Measurements

A Bruker AXS 2020SA TG‐DTA instrument was used to perform thermogravimetric and differential thermal analysis (TG‐DTA). Measurements were performed at a ramp rate of 5 °C min^−1^ under argon using a platinum crucible. Measurements were performed in the temperature ranges of 25–900 °C.

### X‐Ray Photoelectron Spectroscopy (XPS) Measurements

XPS measurements were performed on pristine ${\mathrm{Ag}}_{2}{\mathrm{Ni}}_{2}{\mathrm{TeO}}_{6}$, ${\mathrm{Ag}}_{2}{\mathrm{NiCoTeO}}_{6}$, charged ${\mathrm{Ag}}_{2-x}{\mathrm{Ni}}_{2}{\mathrm{TeO}}_{6}$ and charged ${\mathrm{Ag}}_{2-x}{\mathrm{NiCoTeO}}_{6}$ electrodes to ascertain the valency state upon silver‐ion extraction. The electrodes were intimately washed with super‐dehydrated acetonitrile and dried inside an argon‐filled glove box, prior to undertaking XPS analyses at Ag3d, Co2p, Te3d, and Ni2p binding energies. A hermetically sealed vessel was used to transfer the electrode samples into the XPS machine (JEOL(JPS‐9030) equipped with both Mg *K*α and Al *K*α sources). For clarity, XPS analyses at Te3d, Ag3d, and Co2p binding energies were conducted using the Al *K*α source, whereas the Mg *K*α source was used for analyses at Ni2p binding energies. The electrodes were etched by an Ar‐ion beam for 10 s to eliminate the passivation layer at the surface. The attained XPS spectra were fitted using Gaussian functions, and data processing protocols were performed using COMPRO software.

### X‐Ray Absorption Spectroscopy (XAS) Measurements

Charged ${\mathrm{Ag}}_{2-x}{\mathrm{Ni}}_{2}{\mathrm{TeO}}_{6}$ electrodes were hermetically sealed in packets inside an Ar‐purged glove box. The XAS spectra were measured in the Ni *K*‐edge and Co *K*‐edge energy region (at room temperature) in transmission mode at beamline BL14B2 of the SPring‐8 (Japan) synchrotron facility. Athena package was used to treat the raw X‐ray absorption data, as is customary. As for the O *K*‐edge measurements, the (dis)charged electrode samples were transferred to a measurement vacuum chamber without air exposure. The spectra were measured in fluorescence yield mode (which is sensitive to the bulk state of a sample) using the beamline facility (BL1N2) of Aichi Synchrotron Radiation Center located at Aichi (Japan).

### Computational Methods

The charge density for ${\mathrm{Ag}}_{2}{\mathrm{Ni}}_{2}{\mathrm{TeO}}_{6}$, ${\mathrm{Ag}}_{6}{\mathrm{Ni}}_{2}{\mathrm{TeO}}_{6}$, AgCl and ${\mathrm{Ag}}_{2}O$ were optimized to be self‐consistent (with a threshold of 10^−7^ eV) using the density functional theory (DFT) formalism with generalized gradient approximation (GGA), incorporating on‐site Coulomb parameters and dispersion force correction. The DFT calculations were performed by Vienna ab‐initio Simulation Package (VASP) programme.^[^ ^117^, ^118^, ^119^, ^120^ ^]^ The inner core region was assessed using the projector‐augmented‐wavefunction method.^[^ ^121^ ^]^ Thus, the Kohn–Sham equations^[^ ^26^ ^]^ were solved only for the valence electrons. The number and occupancy of (valence) electrons was set as follows: Ag ($4d^{10}5s^{1}$), Ni ($3d^{8}4s^{2}$), Te ($5s^{2}5p^{4}$), Cl ($3s^{2}3p^{5}$), and O ($2s^{2}2p^{4}$). Thus, other degenerate states of silver encountered in the present work were not considered in our preliminary simulation efforts availed in Supporting Information (Figure S26 and Note S1, Supporting Information).

### Ionic Conductivity Measurements

As‐prepared ${\mathrm{Ag}}_{2}{\mathrm{Mg}}_{2}{\mathrm{TeO}}_{6}$ and ${\mathrm{Ag}}_{2}{\mathrm{Ni}}_{2}{\mathrm{TeO}}_{6}$ powder samples were uniaxially pressed into pellets with diameters of 10 mm under a pressure of about 40 MPa. Stainless steel (SUS) was used as the current collector. The pellet densities were ≈ 74 % and 84 % of the theoretical ceramic densities, respectively. Electrochemical impedance measurements were done using a two‐probe alternating current (a.c.) impedance spectroscopy (VSP‐300 (Bio‐Logic Science Instruments Corp.)) over a frequency ranging from 100 mHz to 3 MHz at a perturbation of 10 mV. Impedance spectroscopic data was initially collected at 25 °C and thereafter between 30 and 100 °C, with impedance scans taken every 15 °C. Ionic conductivities (of the bulk) recorded at various temperatures were obtained by Nyquist plot fittings. Nyquist plots displayed typical behavior of ion‐conducting material, which includes a semicircle at high frequencies and a linear spike at low frequencies. The activation energy (*E*_a_) for silver‐ion conduction was calculated through a linear fitting of the bulk ionic conductivity values at various temperatures by incorporating the well‐established Arrhenius equation, $\sigma =\sigma_{0}\exp\left(-E_{a}/k_{B}T\right)$ plotted in its log form (straight line equation: $\log\left(\sigma T\right)=-E_{a}/k_{B}T+\log\left(\sigma_{0}T\right)$) versus inverse temperature, 1/T with a gradient $-E_{a}/k_{B}$ and *y*‐intercept $\log(\sigma_{0}T)$. Here, σ denotes the temperature‐contingent ionic conductivity, σ_0_ as the absolute ionic conductivity (at zero temperature), *E*_a_ represents the activation energy (in this case, for silver‐ion conduction), whilst *k*_B_ and *T* are the Boltzmann constant and temperature, respectively. All equivalent circuits of the Nyquist plots were fitted using the EC‐Lab software package Z‐fit.

## Conflict of Interest

The authors declare no conflict of interest.

## Author Contributions

T.M. and G.M.K. planned the project; T. M. supervised all aspects of the research with help from G.M.K. and Z.‐D.H; J.R. prepared the honeycomb‐layered oxide materials with the help from T.M.; N.T., Y.M., M.I., and T.S. acquired and analyzed TEM data with input from H.S., G.M.K., and T.M.; K.Y. and T.M. performed the electrochemical measurements with input from G.M.K., A.A., H.S., and Z.‐D.H; Y.O. acquired the high‐resolution X‐ray diffraction data; H.U., C.T., and H.K. helped in the analyses of the X‐ray diffraction data; S.T. and Y.O. acquired X‐ray absorption spectroscopy (XAS) data and conductivity data; K.Y. performed X‐ray photoelectron spectroscopic measurements with input from T.M. and G.M.K; K.K. performed and analyzed the thermal stability measurements; K.T. performed DFT calculations with input from G.M.K. and T.M. The theoretical discussion including the mathematical framework for the origin of the bilayers in Ag‐based tellurates was entirely conceived and written by G.M.K. and T.M. The manuscript was written by Y.M., J.R., T.S., K.T., G.M.K., Z.‐D.H., and T.M. All authors contributed to discussions and were given the chance to make comments and contributions pertaining the content in the manuscript and accompanied Supporting Information.

## Supporting information

Supporting Information

## Data Availability

The data that support the findings of this study are openly available in arXiv at https://doi.org/10.48550/arXiv.2112.07355, reference number 211207355.
