# Supplementary material for: Honeycomb‐Layered Oxides With Silver Atom Bilayers and Emergence of Non‐Abelian SU(2) Interactions
Source: Adv Sci (Weinh). 2022 Dec 27;10(6):2204672. doi: 10.1002/advs.202204672 (PMC9951339; doi:10.1002/advs.202204672)
Supplement: Supplementary file 1 — Supporting Information [file ADVS-10-2204672-s001.pdf]

Supplementary Information

Honeycomb Layered Oxides With Silver Atom Bilayers and Emergence of Non-Abelian SU(2) Interactions

Titus Masese[1,2*], Godwill Mbiti Kanyolo[1,3*], Yoshinobu Miyazaki[4], Miyu Ito[4], Noboru Taguchi[5], Josef Rizell[1,5], Shintaro Tachibana[6], Kohei Tada[1], Zhen-Dong Huang[7], Abbas Alshehabi[8], Hiroki Ubukata[9], Keigo Kubota[1], Kazuki Yoshii[1], Hiroshi Senoh[1], Cédric Tassel[9], Yuki Orikasa[6], Hiroshi Kageyama[9] & Tomohiro Saito[4]

[1] Research Institute of Electrochemical Energy, National Institute of Advanced Industrial Science and Technology (AIST), 1–8–31 Midorigaoka, Ikeda, Osaka 563–8577, JAPAN

[2] AIST-Kyoto University Chemical Energy Materials Open Innovation Laboratory (ChEM–OIL), Sakyo–ku, Kyoto 606–8501, JAPAN

[3] Department of Engineering Science, The University of Electro–Communications, 1–5–1 Chofugaoka, Chofu, Tokyo 182–8585, JAPAN

[4] Tsukuba Laboratory, Technical Solution Headquarters, Sumika Chemical Analysis Service (SCAS), Ltd.,Tsukuba, Ibaraki 300–3266, JAPAN

[5] Department of Physics, Chalmers University of Technology, SE–412 96 Göteborg, SWEDEN.

[6] Graduate School of Life Sciences, Ritsumeikan University, 1–1–1 Noji-higashi, Kusatsu, Shiga 525–8577, JAPAN

[7] Key Laboratory for Organic Electronics and Information Displays and Institute of Advanced Materials (IAM), Nanjing University of Posts and Telecommunications (NUPT), Nanjing, 210023, CHINA

[8] Department of Industrial Engineering, National Institute of Technology (KOSEN), Ibaraki College, 866 Nakane, Hitachinaka, Ibaraki 312–8508, JAPAN

[9] Department of Energy and Hydrocarbon Chemistry, Graduate School of Engineering, Kyoto University, Nishikyo-ku, Kyoto 615–8510, JAPAN

[*]Correspondence and material requests should be addressed to: Titus Masese (Lead contact) (titus.masese@aist.go.jp)

Phone: +81–72–751–9224; Fax: +81–72–751–9609

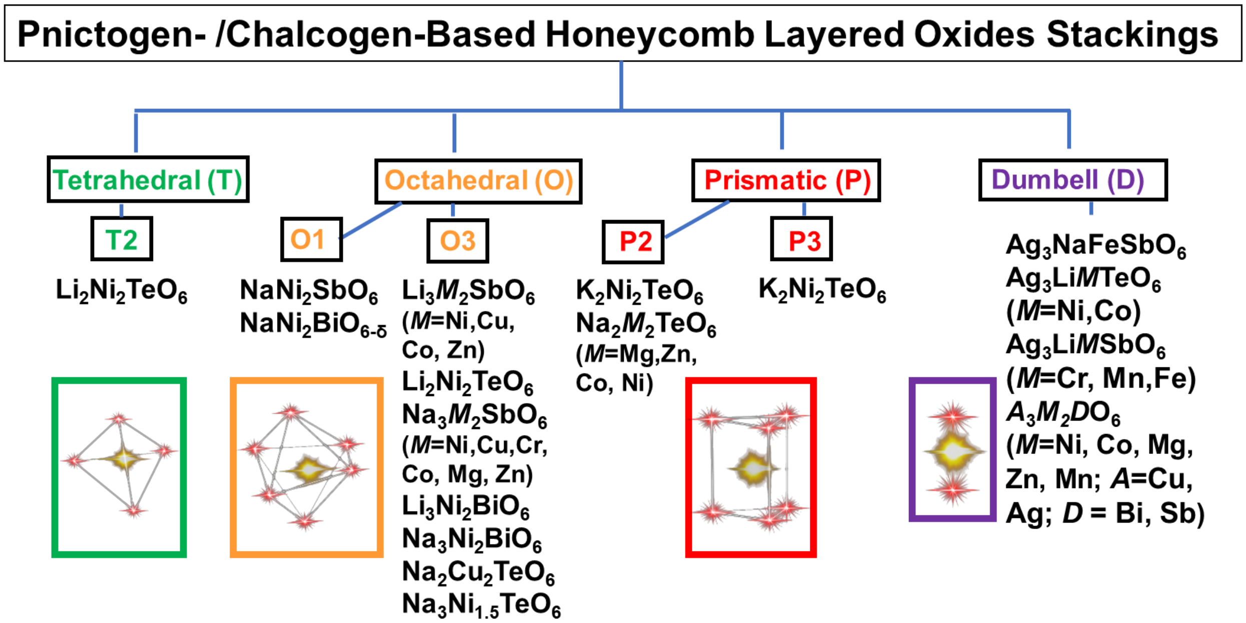

Supplementary Figure 1. Stacking arrangement of alkali or coinage metal atoms in representative pnictogen- and chalcogen-based honeycomb layered oxides. The stacking arrangements have been denoted in the Hagenmuller-Delmas notation. For instance, Li atoms in $Li_2Ni_2TeO_6$ tend to form stronger tetrahedral coordinations between Li atoms and oxygen atoms, with 2 repetitive honeycomb layers (comprising NiO_6 and TeO_6 octahedra) for each unit cell. This type of structure is typically referred to as T2-type in the Hagenmuller-Delmas notation (where 'T' is the tetrahedral coordination and '2' is the number of honeycomb slabs per unit cell). Further, atoms with larger ionic radii such as Na in for instance $Na_3Ni_2SbO_6$ adopt an O3-type layered framework (where 'O' is an octahedral coordination with a periodicity of three honeycomb slabs per unit cell) whilst Na in $Na_2Ni_2TeO_6$ adopt a P2-type framework (where 'P' is a prismatic coordination with a periodicity of two). Ag atoms in $Ag_3Ni_2SbO_6$ are coordinated linearly with two oxygen atoms forming dumbbell-like coordinations and are denoted as 'D' in this notation.

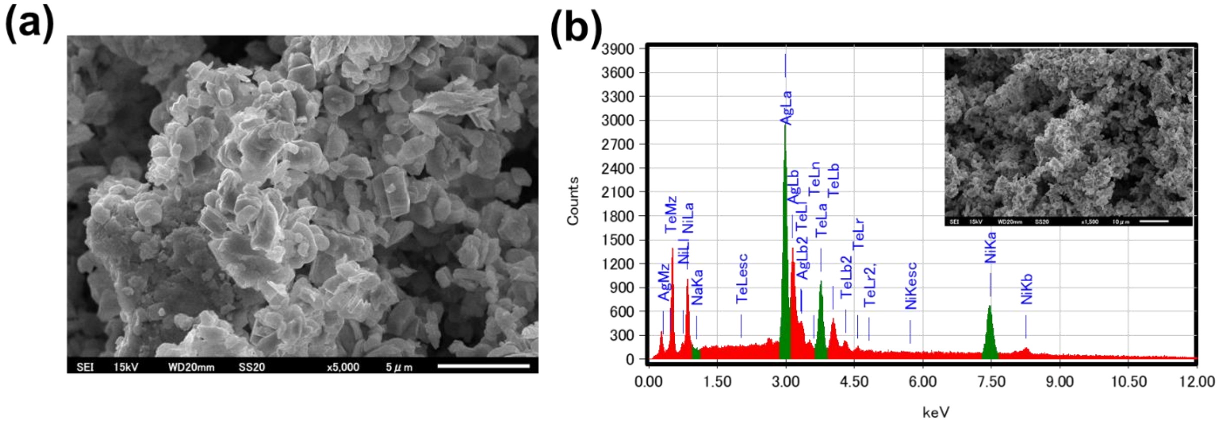

Supplementary Figure 2. Particle morphology and elemental composition of as-prepared $Ag_2Ni_2TeO_6$ synthesised via a low-temperature synthetic route. **(a)** Scanning electron microscopy (SEM) images of the pristine $Ag_2Ni_2TeO_6$ powders **(b)** Energy dispersive X-ray (EDX) spectra for a selected section (shown in inset) confirming the elemental composition of the as-prepared $Ag_2Ni_2TeO_6$.

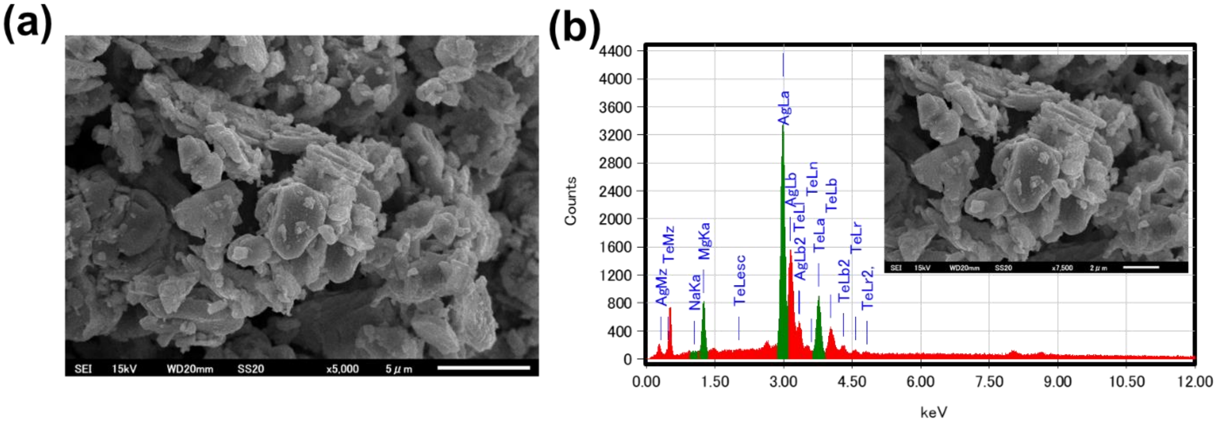

Supplementary Figure 3. Particle morphology and elemental composition of as-prepared $Ag_2Mg_2TeO_6$ synthesised via a low-temperature synthetic route. **(a)** Scanning electron microscopy (SEM) images of the pristine $Ag_2Mg_2TeO_6$ powders **(b)** Energy dispersive X-ray (EDX) spectra for a selected section (shown in inset) confirming the elemental composition of the as-prepared $Ag_2Mg_2TeO_6$.

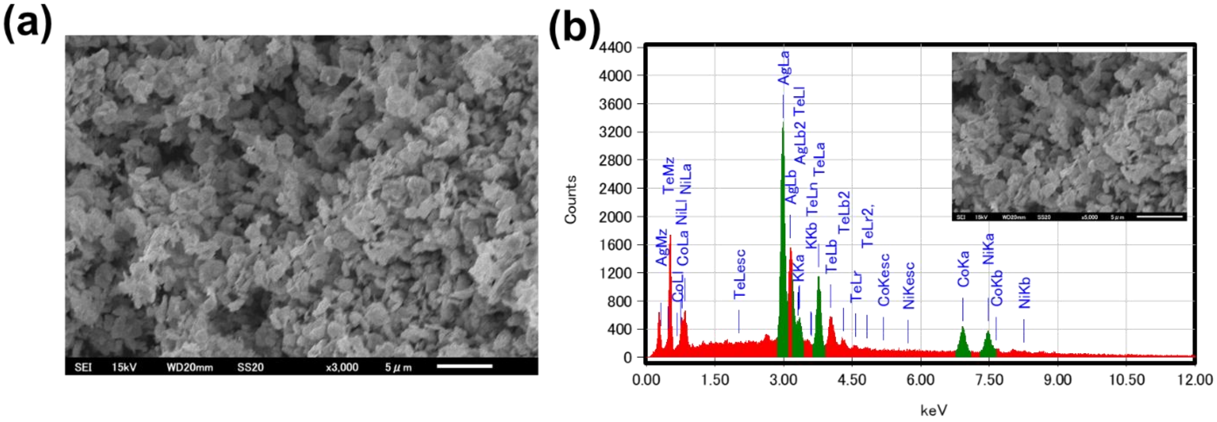

Supplementary Figure 4. Particle morphology and elemental composition of as-prepared $Ag_2NiCoTeO_6$ synthesised via a low-temperature synthetic route. **(a)** Scanning electron microscopy (SEM) images of the pristine $Ag_2NiCoTeO_6$ powders **(b)** Energy dispersive X-ray (EDX) spectra for a selected section (shown in inset) confirming the elemental composition of the as-prepared $Ag_2NiCoTeO_6$.

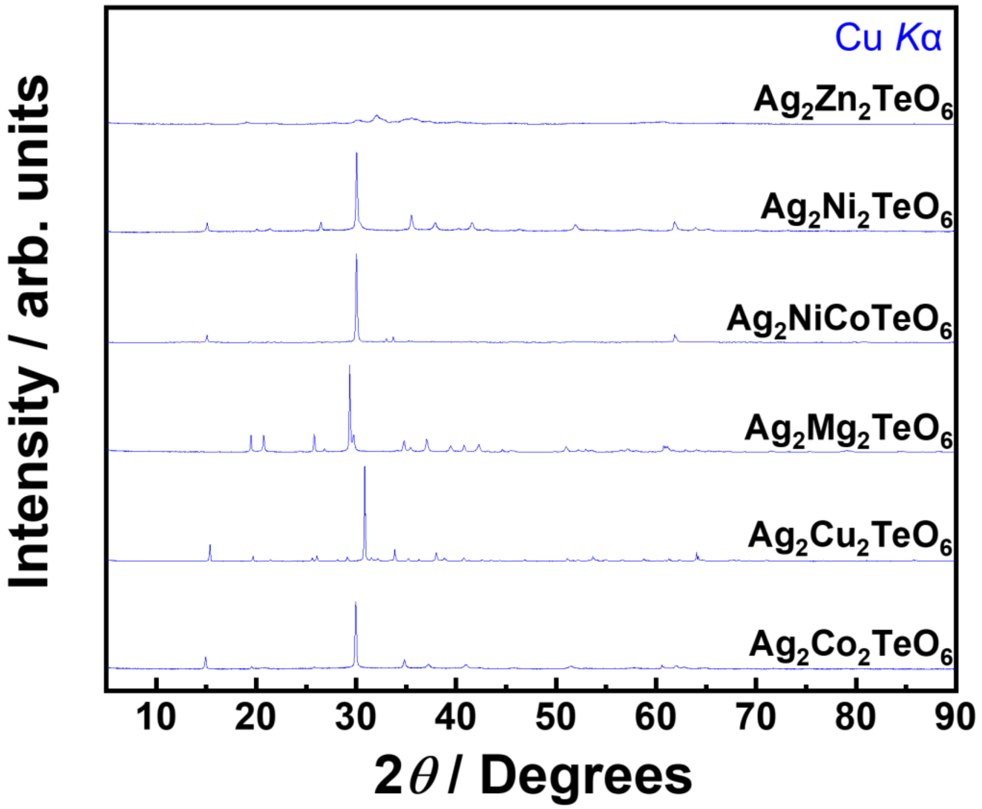

Supplementary Figure 5. Conventional X-ray diffraction (XRD) patterns of as-prepared honeycomb layered tellurates with a global composition of $Ag_2M_2TeO_6$ (M =Ni, Co, Mg, Zn, Cu) prepared via ion-exchange route. Measurements were taken at the Cu $K\alpha$ wavelength. XRD patterns of as-prepared $Ag_2M_2TeO_6$ (M = Ni, Co, Mg, Zn, Cu) showing the possible design of related honeycomb tellurates as $Ag_2Ni_2TeO_6$.

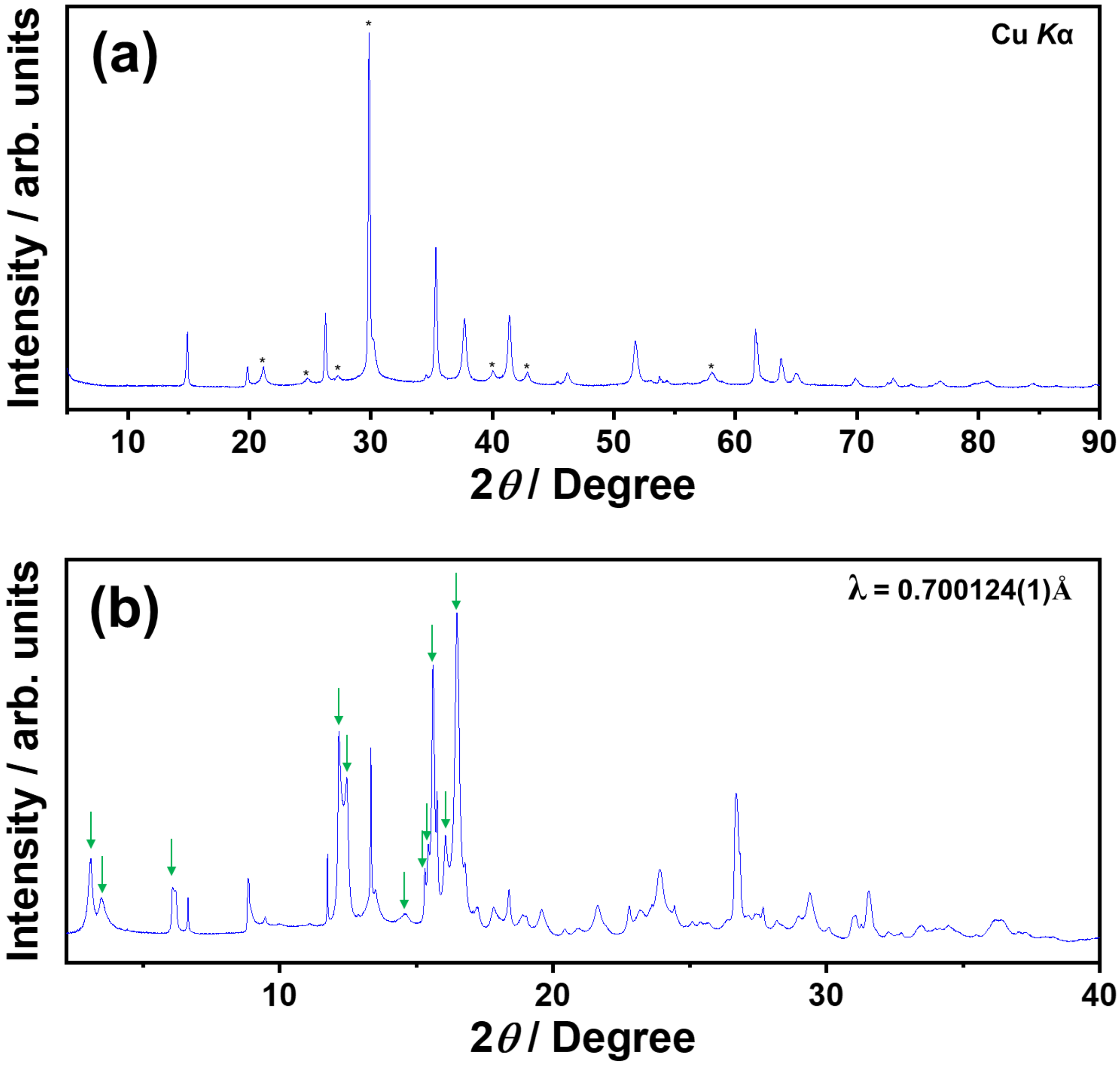

Supplementary Figure 6. Comparison of the conventional XRD data and the synchrotron XRD data derived from global composition $Ag_2Ni_2TeO_6$. **(a)** Conventional XRD data taken at a wavelength of Cu *K*α. Some of the peaks that show significant broadening and asymmetry are highlighted in asterisks. **(b)** Synchrotron XRD data taken at a wavelength of 0.700124(1)Å, showing the emergence of other peaks (some of which are shown in green arrows). The emergence of additional Bragg diffraction peaks shows the X-ray induced damage on the samples.

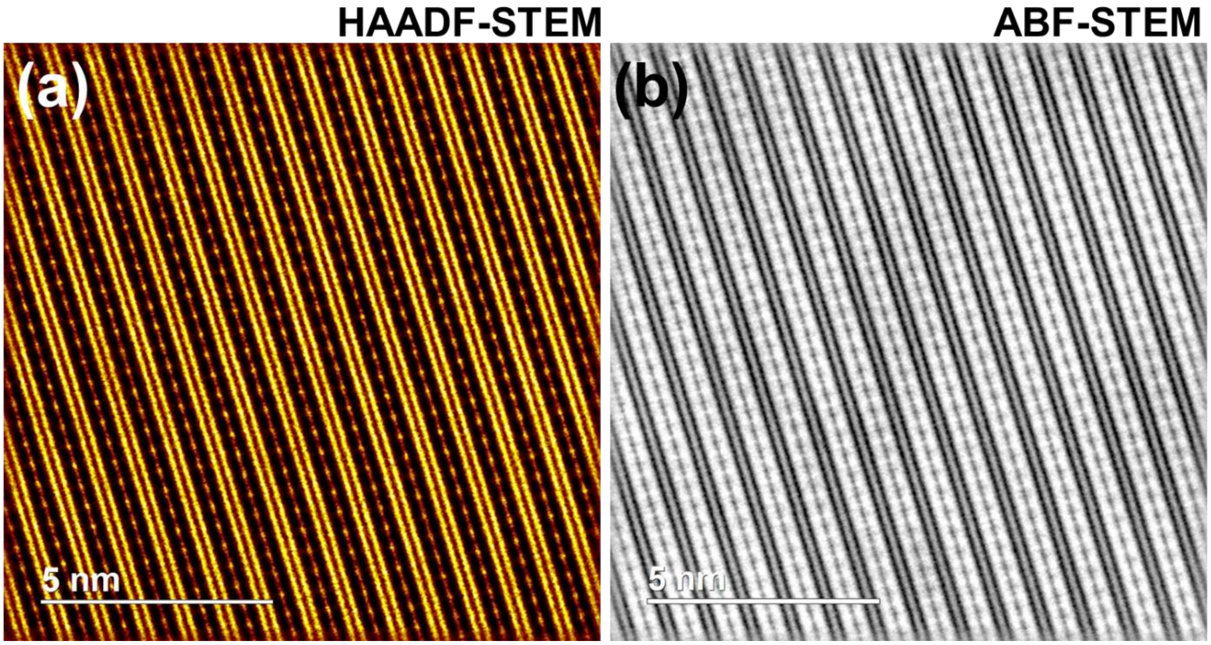

Supplementary Figure 7. High-resolution scanning transmission electron microscopy (STEM) imaging of bilayered domains of the Ag-rich $Ag_6Ni_2TeO_6$ within the global composition, $Ag_2Ni_2TeO_6$ taken along [110] zone axis. **(a)** High-angle annular dark-field (HAADF)-STEM images taken along the [110] zone axis also showing the bilayer arrangement of Ag atoms and **(b)** Corresponding annular bright-field (ABF)-STEM image.

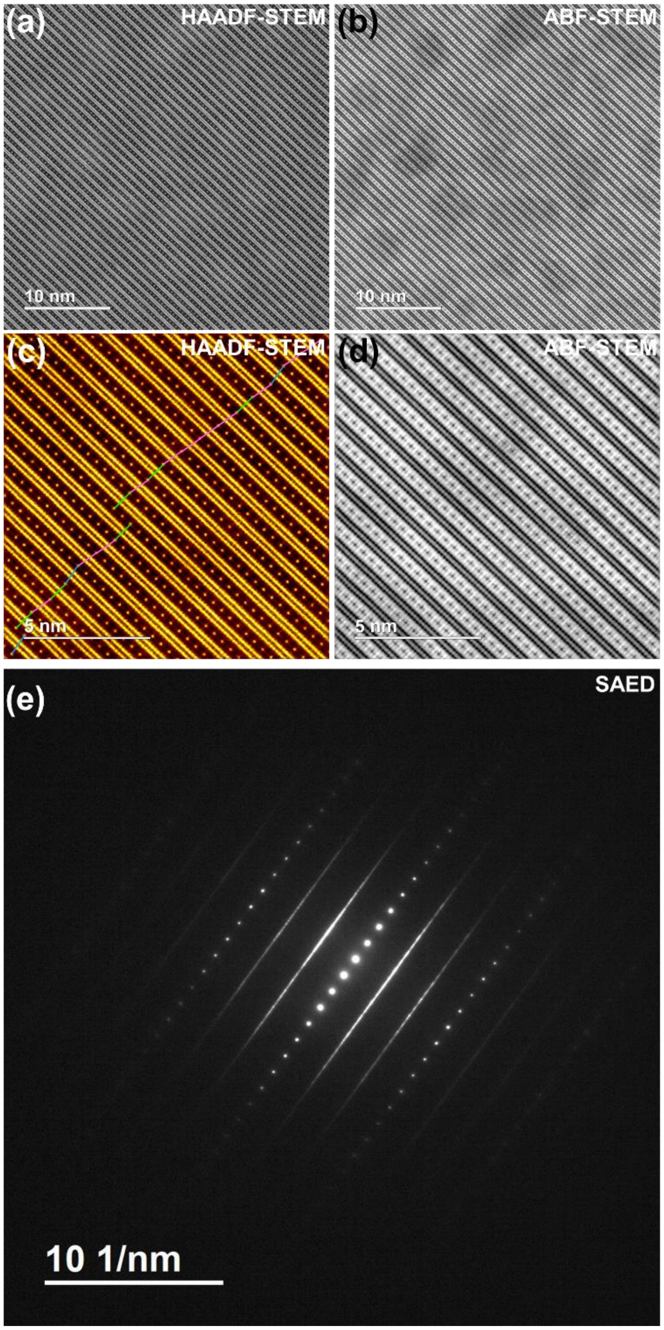

Supplementary Figure 8. High-resolution STEM imaging of $Ag_6Mg_2TeO_6$ along the [100] zone axis, showing a bilayer arrangement of silver atoms. **(a)** Low-magnification HAADF-STEM image of $Ag_6Mg_2TeO_6$ taken along the [100] zone axis and **(b)** Corresponding ABF-STEM image. **(c)** High-resolution HAADF-STEM image showing shifts in the alignment of the transition metal slabs along the *c*-axis as highlighted in coloured lines and **(d)** Corresponding ABF-STEM image. **(e)** Selected area electron diffraction pattern (SAED) pattern highlighting streaks in the diffraction spots.

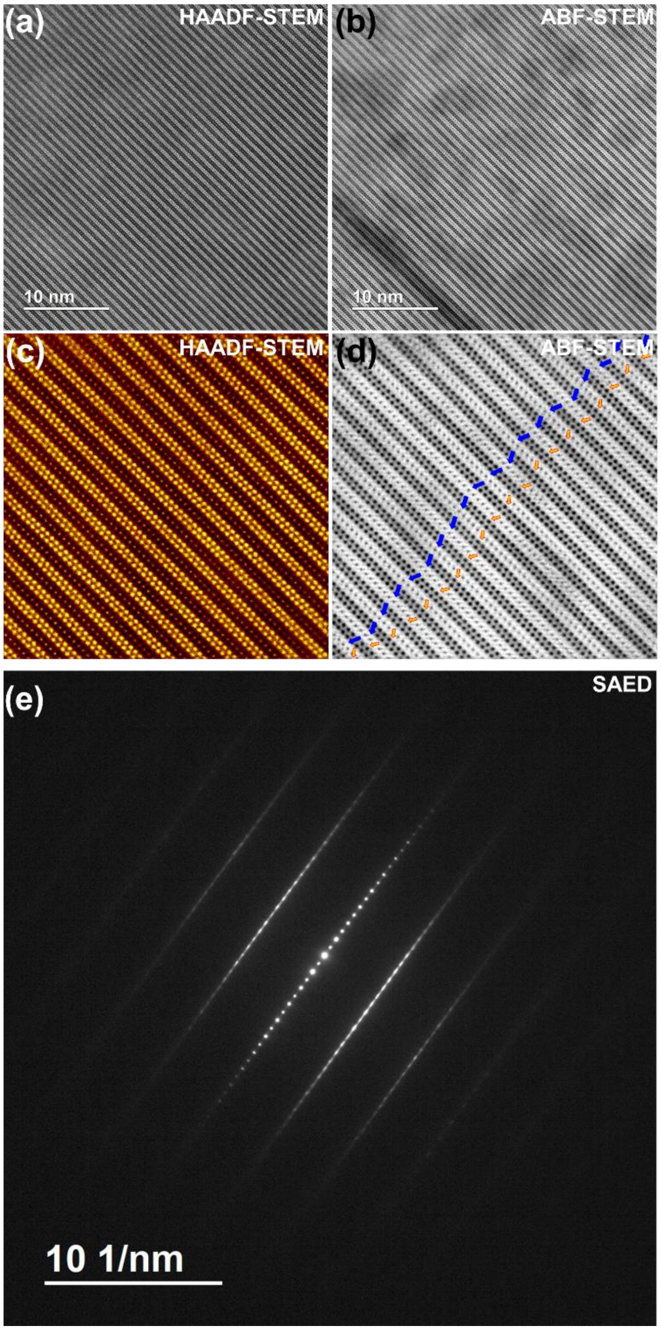

Supplementary Figure 9. High-resolution STEM imaging of $Ag_6Mg_2TeO_6$ along the [1 $\bar{1}$ 0] zone axis, showing a bilayer arrangement of silver atoms. **(a)** Low-magnification HAADF-STEM image of $Ag_6Mg_2TeO_6$ taken along the [1 $\bar{1}$ 0] zone axis and **(b)** Corresponding ABF-STEM image. **(c)** High-resolution HAADF-STEM image and (d) Corresponding ABF-STEM image showing alternating shifts in the orientation of oxygen atoms (orange arrows) and the alignment of Ag bilayers (blue arrows) along the *c*-axis, highlighted by the coloured lines. **(e)** SAED pattern highlighting streaks in the diffraction spots.

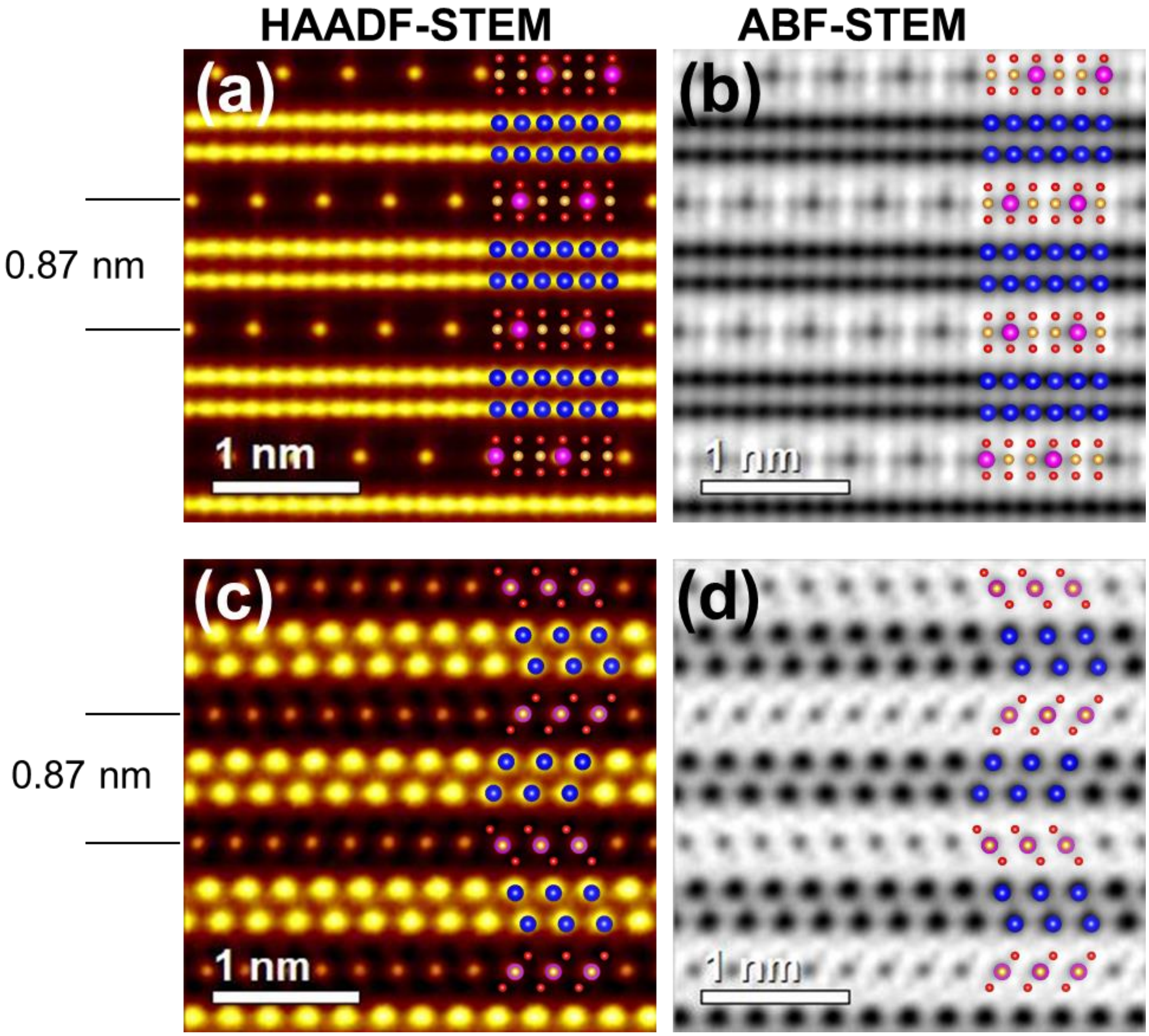

Supplementary Figure 10. Arrangement of the atoms along the [100] and the [1 $\bar{1}$ 0] zone axes in $Ag_6Mg_2TeO_6$. **(a)** HAADF-STEM image of $Ag_6Mg_2TeO_6$ taken along the [100] zone axis showing the ordering sequence of Ni and Te atoms across the slabs. Inset shows a projected model of the crystal structure, for clarity. Ag atoms are shown in blue whilst Te and Mg atoms are shown in pink and brown, respectively. **(b)** ABF-STEM image taken along the [100] zone axis displaying the arrangement of silver and oxygen atoms. **(c)** Visualisation (along the [1 $\bar{1}$ 0] zone axis) using HAADF-STEM, and **(d)** Corresponding ABF-STEM image.

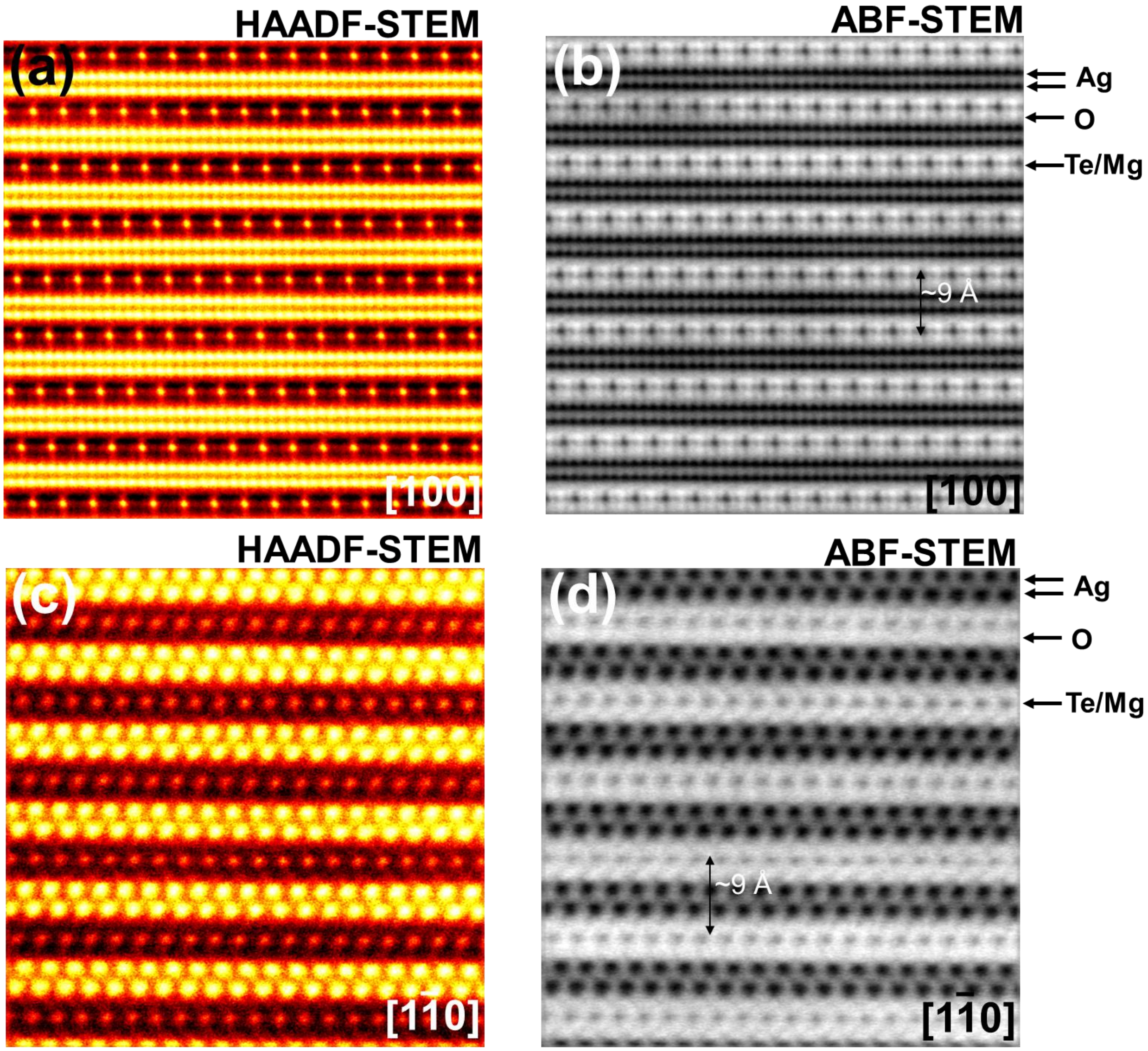

Supplementary Figure 11. High-resolution STEM imaging of $Ag_6Mg_2TeO_6$ along multiple zone axes, showing a bilayer arrangement of silver atoms. **(a)** HAADF-STEM image of $Ag_6Mg_2TeO_6$ taken along the [100] zone axis and **(b)** Corresponding ABF-STEM image. **(c)** Visualisation (along the [1 $\bar{1}$ 0] zone axis) using HAADF-STEM, and **(d)** Corresponding ABF-STEM image.

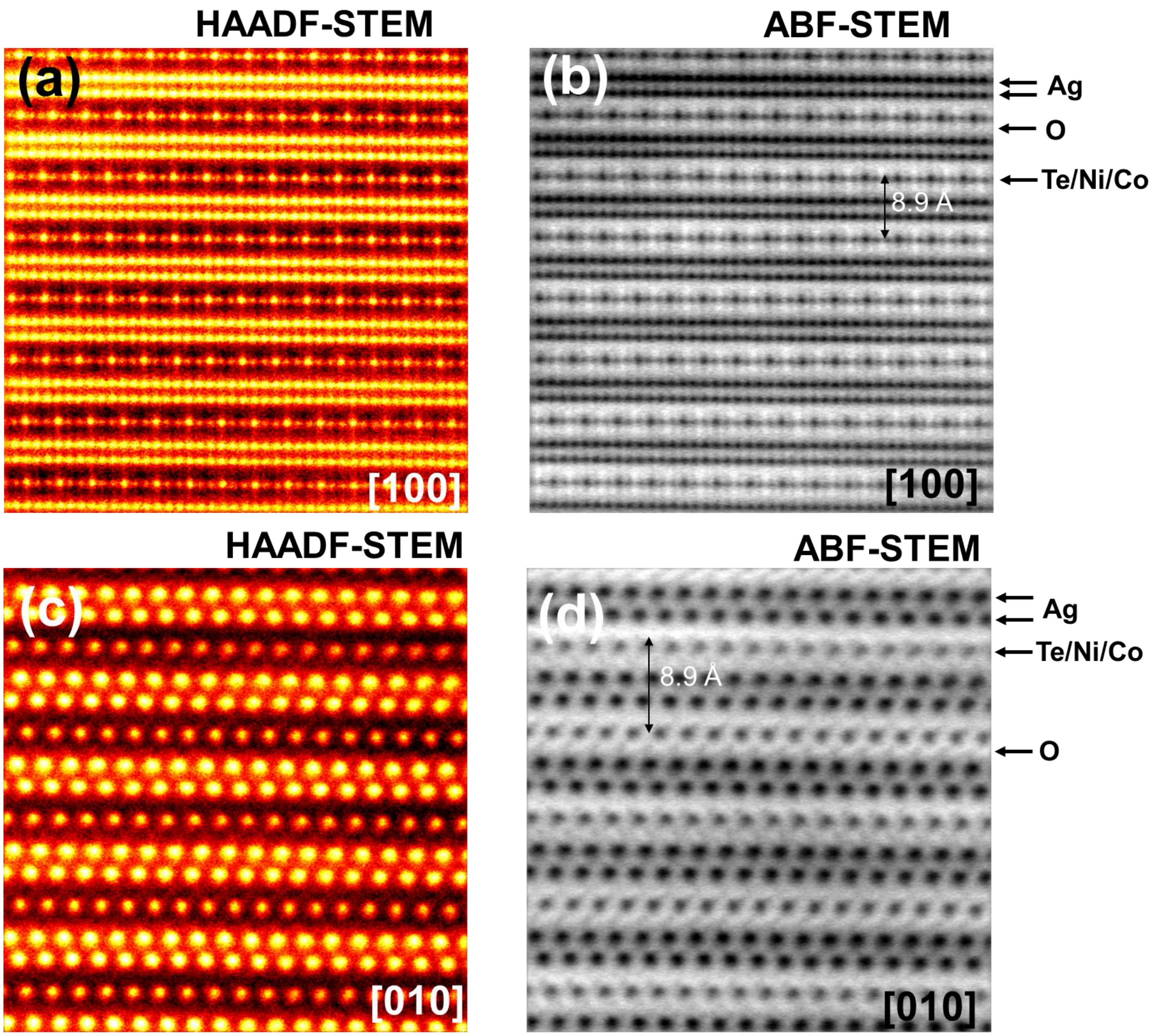

Supplementary Figure 12. High-resolution STEM imaging of $Ag_6NiCoTeO_6$ along multiple zone axes, showing a bilayer arrangement of silver atoms. **(a)** HAADF-STEM image of $Ag_6NiCoTeO_6$ taken along the [100] zone axis and **(b)** Corresponding ABF-STEM image. **(c)** Visualisation (along the [1 $\bar{1}$ 0] zone axis) using HAADF-STEM, and **(d)** Corresponding ABF-STEM image.

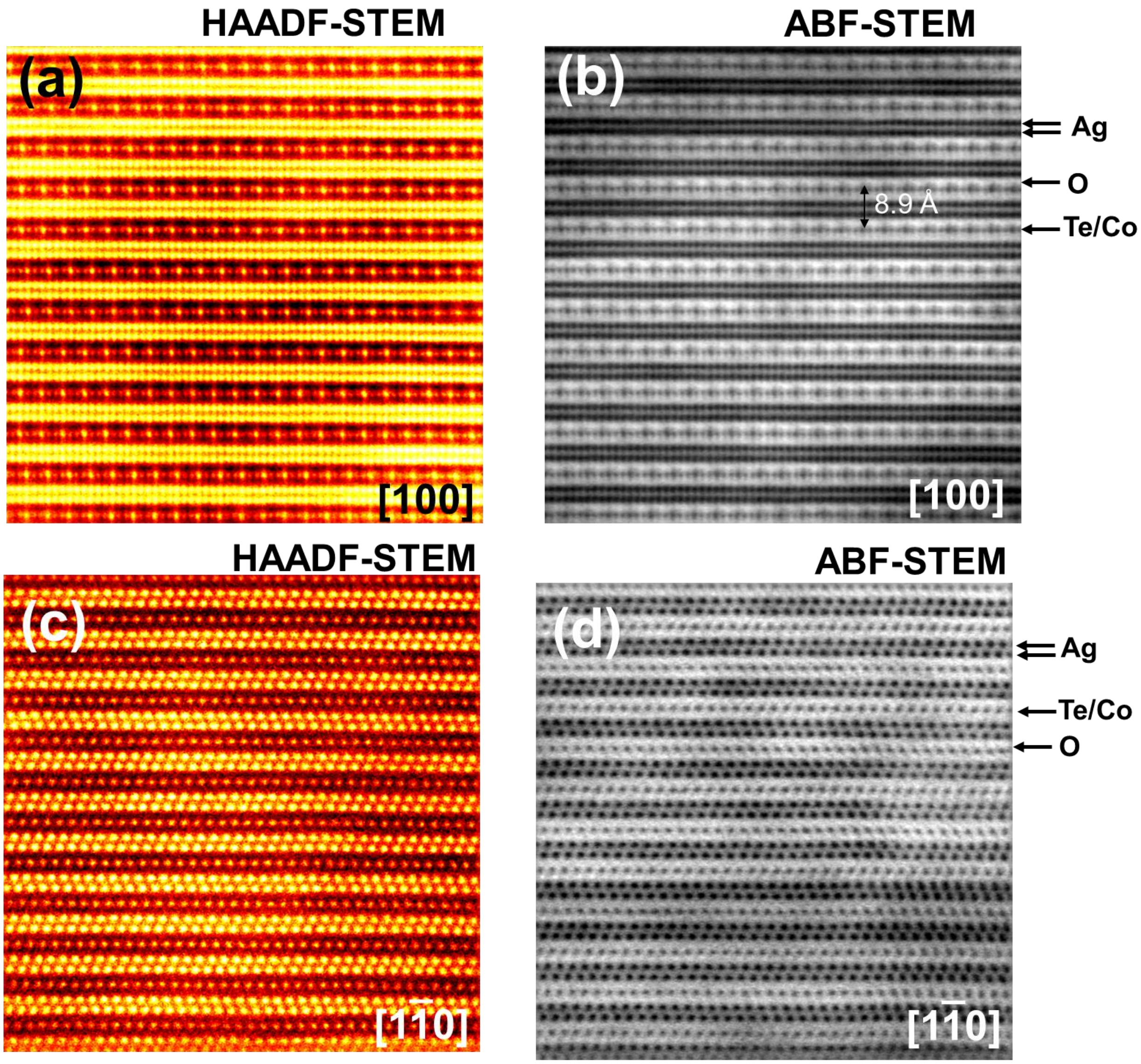

Supplementary Figure 13. High-resolution STEM imaging of $Ag_6Co_2TeO_6$ along multiple zone axes, showing a bilayer arrangement of silver atoms. **(a)** HAADF-STEM image of $Ag_6Co_2TeO_6$ taken along the [100] zone axis and **(b)** Corresponding ABF-STEM image. **(c)** Visualisation (along the [1 $\bar{1}$ 0] zone axis) using HAADF-STEM, and **(d)** Corresponding ABF-STEM image.

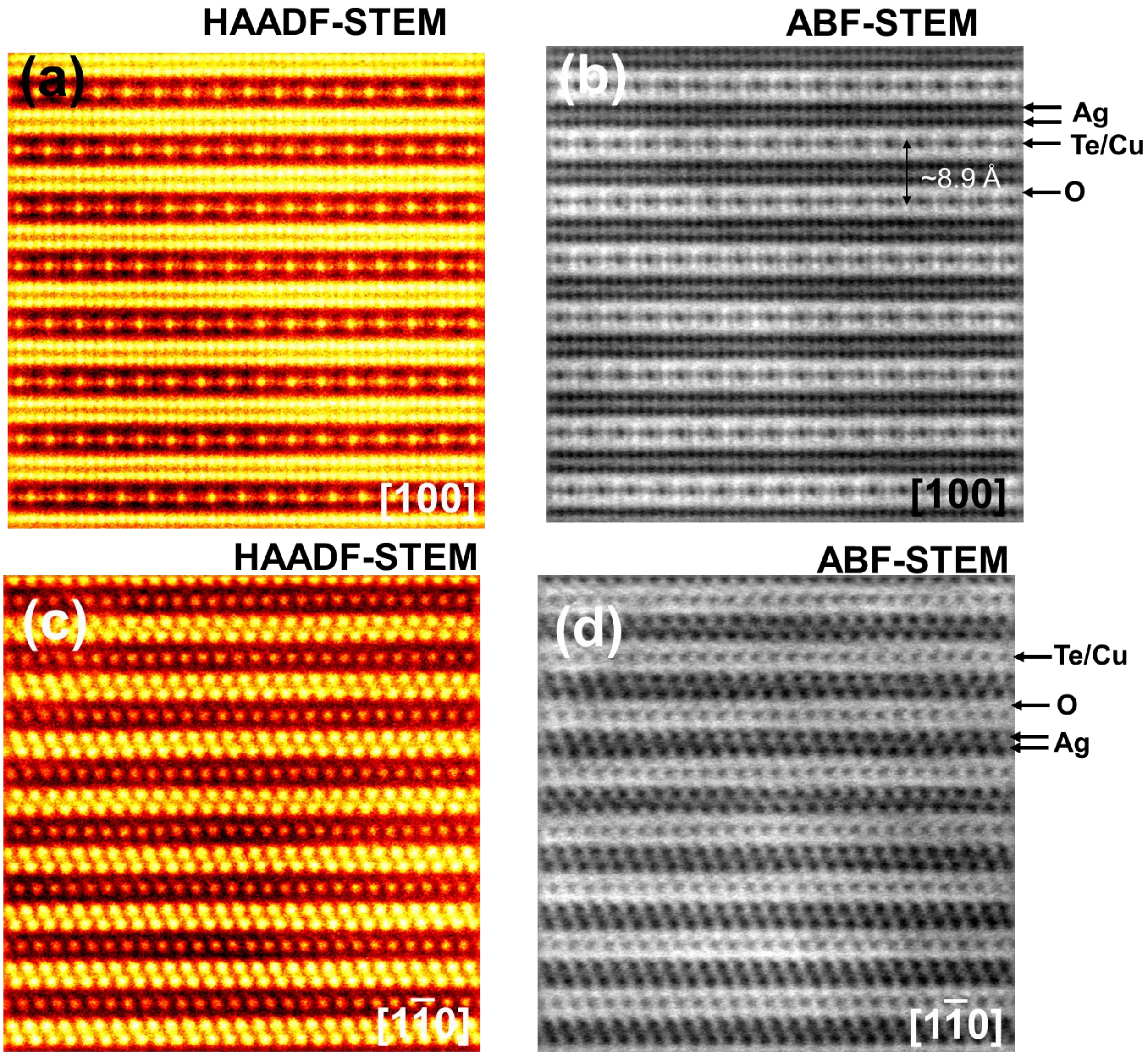

Supplementary Figure 14. High-resolution STEM imaging of $Ag_6Cu_2TeO_6$ along multiple zone axes, showing a bilayer arrangement of silver atoms. **(a)** HAADF-STEM image of $Ag_6Cu_2TeO_6$ taken along the [100] zone axis and **(b)** Corresponding ABF-STEM image. **(c)** Visualisation (along the [1 $\bar{1}$ 0] zone axis) using HAADF-STEM, and **(d)** Corresponding ABF-STEM image.

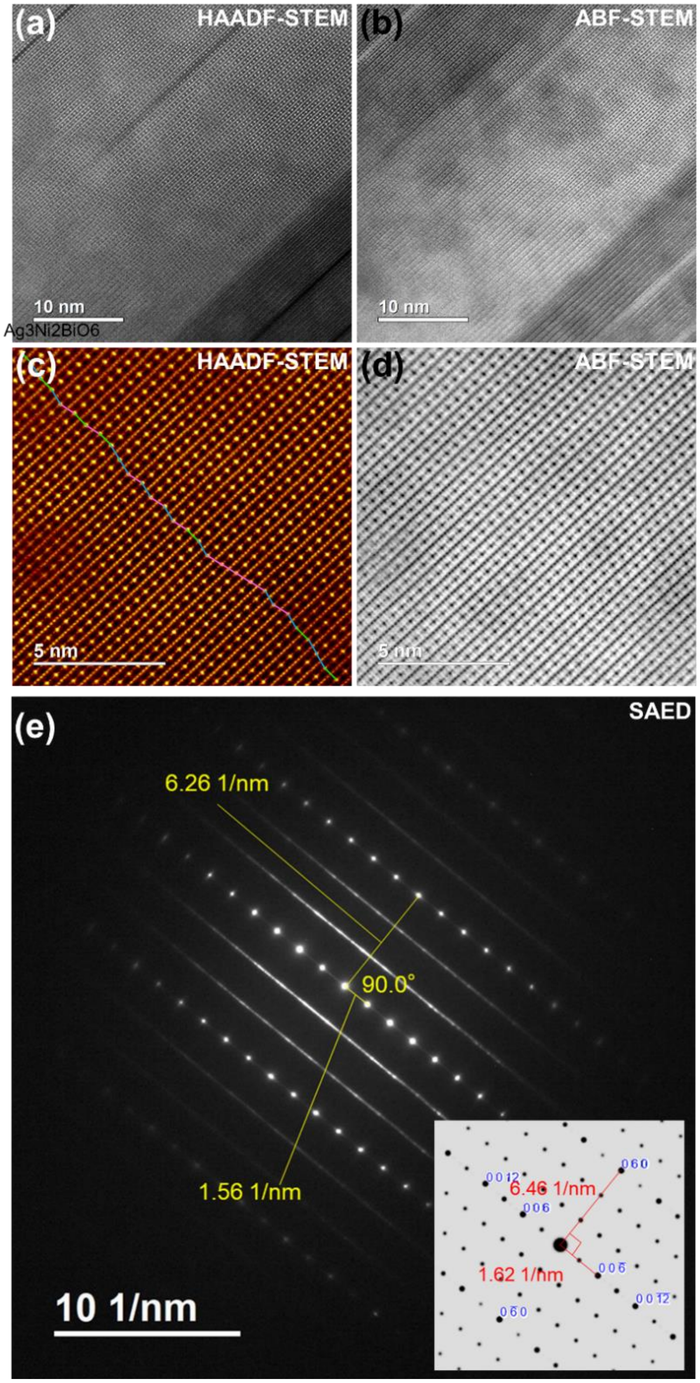

Supplementary Figure 15. High-resolution STEM imaging of $Ag_3Ni_2BiO_6$ along the [100] zone axis, showing a monolayer arrangement of silver atoms. **(a)** Low-magnification HAADF-STEM image of $Ag_3Ni_2BiO_6$ taken along the [100] zone axis and **(b)** Corresponding ABF-STEM image. **(c)** High-resolution HAADF-STEM image showing shifts in the alignment of the transition metal slabs along the *c*-axis as highlighted in coloured lines and (d) Corresponding ABF-STEM image. The right and left shifts of the Ni/Te atom slabs are denoted by green and pink lines, respectively, in (c). **(e)** Selected area electron diffraction pattern (SAED) pattern highlighting streaks in the diffraction spots.

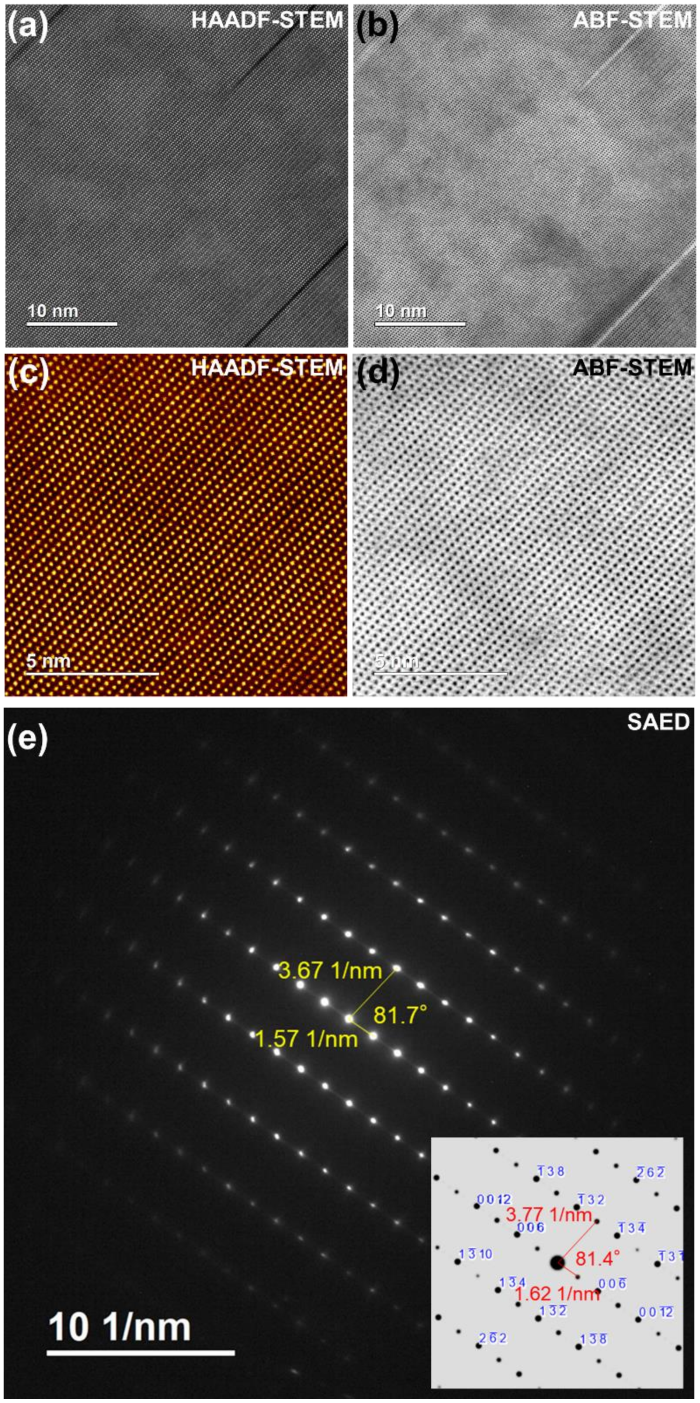

Supplementary Figure 16. High-resolution STEM imaging of $Ag_3Ni_2BiO_6$ along the [310] zone axis, showing a monolayer arrangement of silver atoms. **(a)** Low-magnification HAADF-STEM image of $Ag_3Ni_2BiO_6$ taken along the [310] zone axis and **(b)** Corresponding ABF-STEM image. **(c)** High-resolution HAADF-STEM image and (d) Corresponding ABF-STEM image. **(e)** SAED pattern. No stacking disorders in the arrangement of transition metal atoms is discernible along this zone axis.

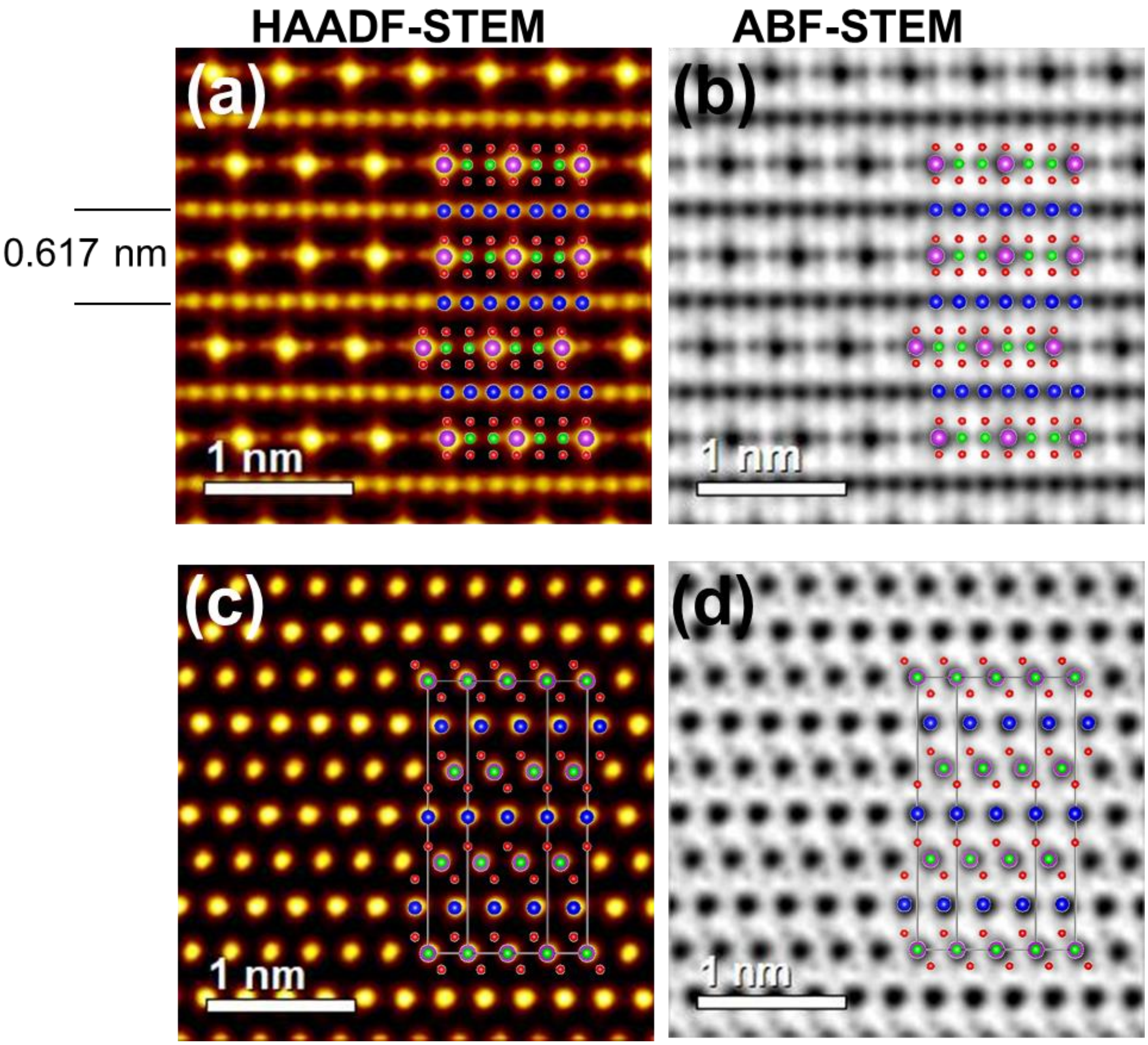

Supplementary Figure 17. Arrangement of the atoms along the [100] and the [310] zone axes in D3-type $Ag_3Ni_2BiO_6$. **(a)** HAADF-STEM image of $Ag_3Ni_2BiO_6$ taken along the [100] zone axis showing the ordering sequence of Ni and Te atoms corresponding to the D3-type stacking. Inset shows a projected model of the crystal structure, for clarity. Ag atoms are shown in blue whilst Bi and Ni atoms are shown in pink and green, respectively. **(b)** ABF-STEM image taken along the [100] zone axis displaying the arrangement of silver atoms. **(c)** Visualisation (along the [310] zone axis) using HAADF-STEM, and **(d)** Corresponding ABF-STEM image.

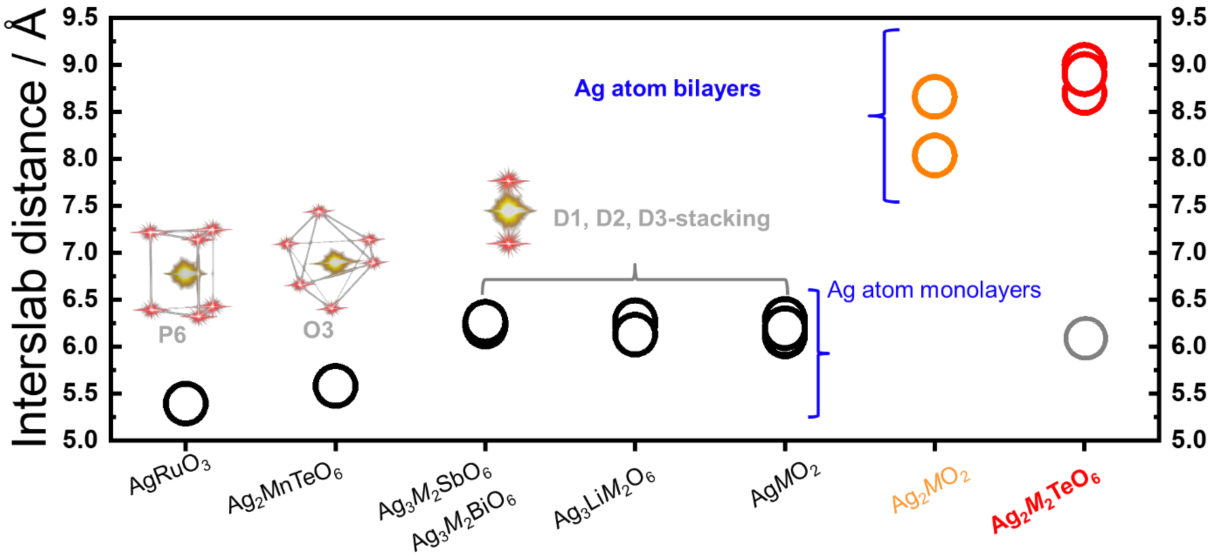

Supplementary Figure 18. Comparative plot of interslab distances observed in silver-based layered oxide materials and their corresponding stacking sequences. The interslab distances of both monolayer and bilayer phase domains in the global $Ag_2M_2TeO_6$ composition have been provided for reference.

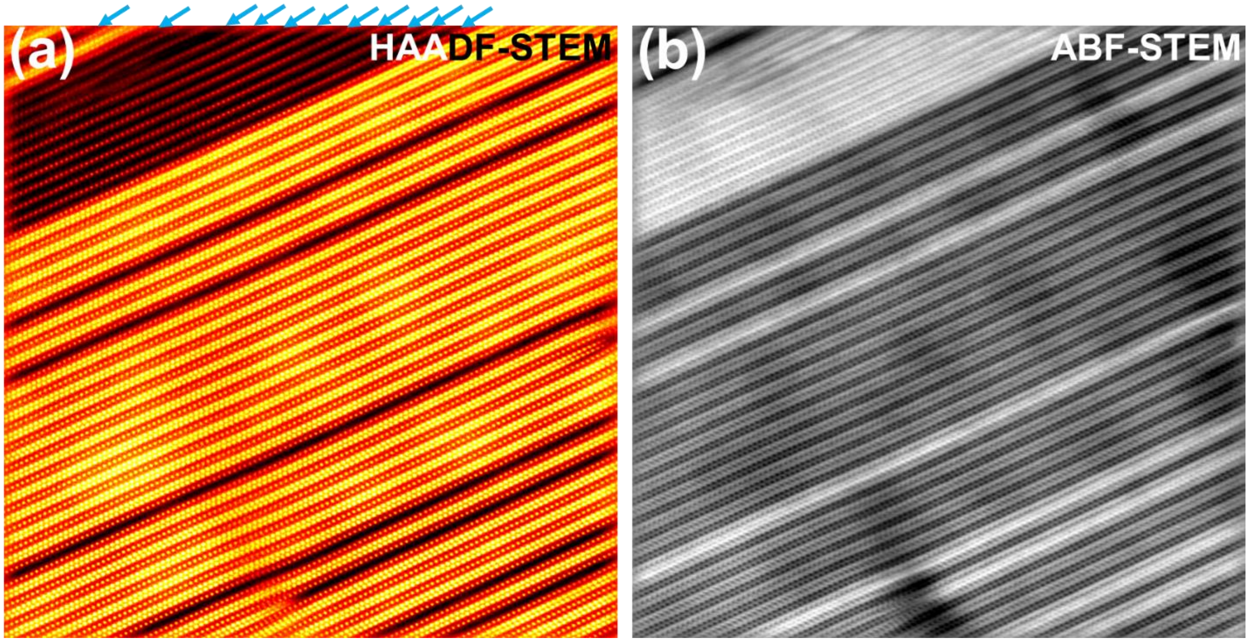

Supplementary Figure 19. High-resolution STEM imaging of $Ag_6Ni_2TeO_6$ crystallite prepared via an equimolar ratio of $AgNO_3$ molten-salt to precursor. **(a)** HAADF-STEM image taken along the [010] zone axis showing Ag bilayers and layers with remanent Na atoms (shown in blue arrows), as validated by STEM-EDX. **(b)** Corresponding ABF-STEM image.

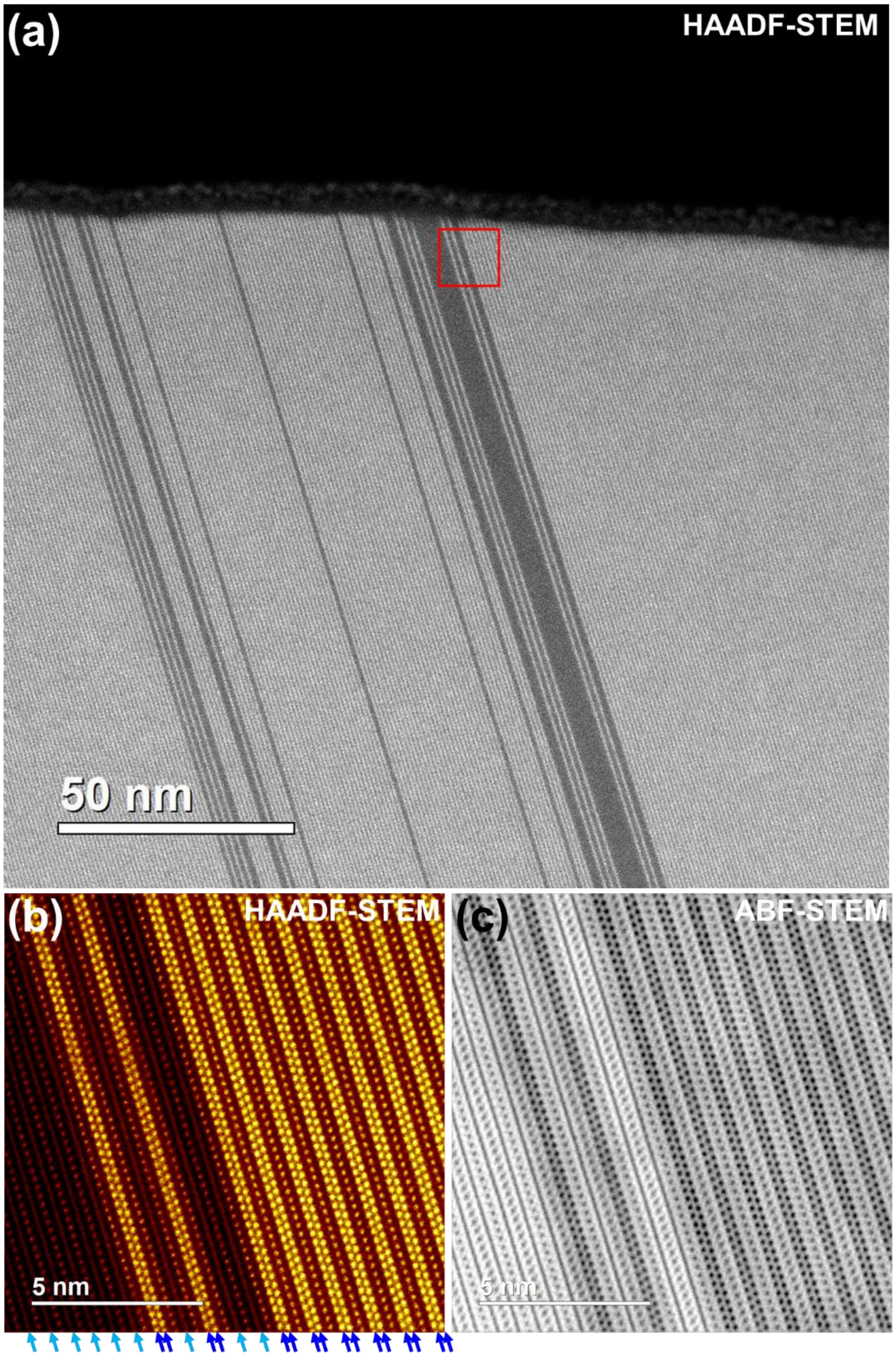

Supplementary Figure 20. (a) HAADF-STEM image of $Ag_6Ni_2TeO_6$ crystallite taken along the [010] zone axis where single Ag atoms layers (in black) were also observed. **(b)** High-resolution HAADF-STEM image of regime highlighted in (a), showing Ag-rich (Ag bilayers) and Ag-poor (Ag monolayer) domains and **(c)** Corresponding ABF-STEM image.

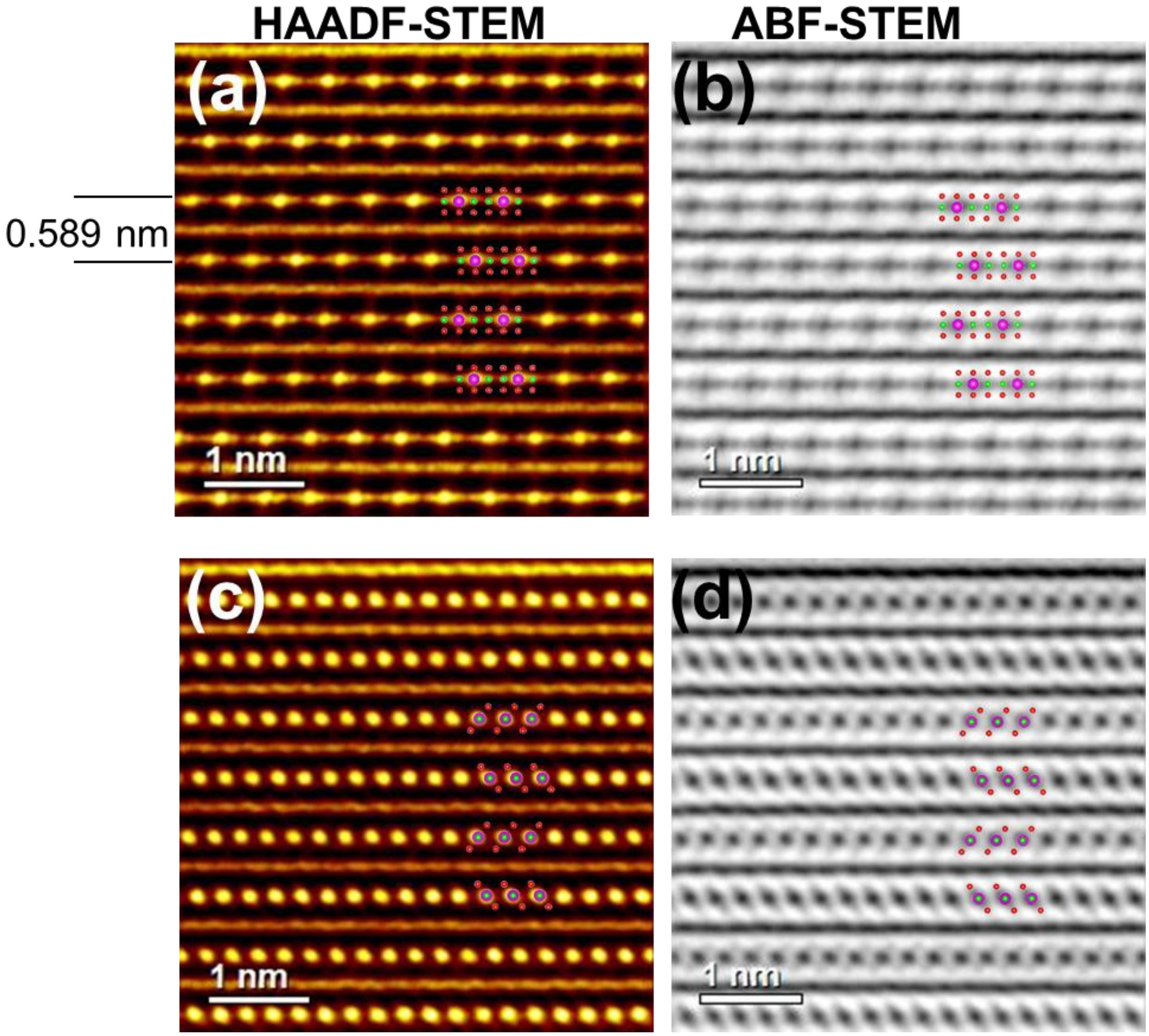

Supplementary Figure 21. High-resolution TEM imaging of monolayer domains in $Ag_2Ni_2TeO_6$ crystallite taken along multiple zone axes. Intergrowths (defects) entailing Ag-poor domains can be observed here. **(a)** HAADF-STEM image of $Ag_2Ni_2TeO_6$ monolayer domains taken along the [100] zone axis and **(b)** Corresponding ABF-STEM image. **(c)** Visualisation (along the [010] zone axis) using HAADF-STEM, and **(d)** Corresponding ABF-STEM image. The STEM images highlight domains where single layers of silver arranged randomly (or in an amorphous state) were observed.

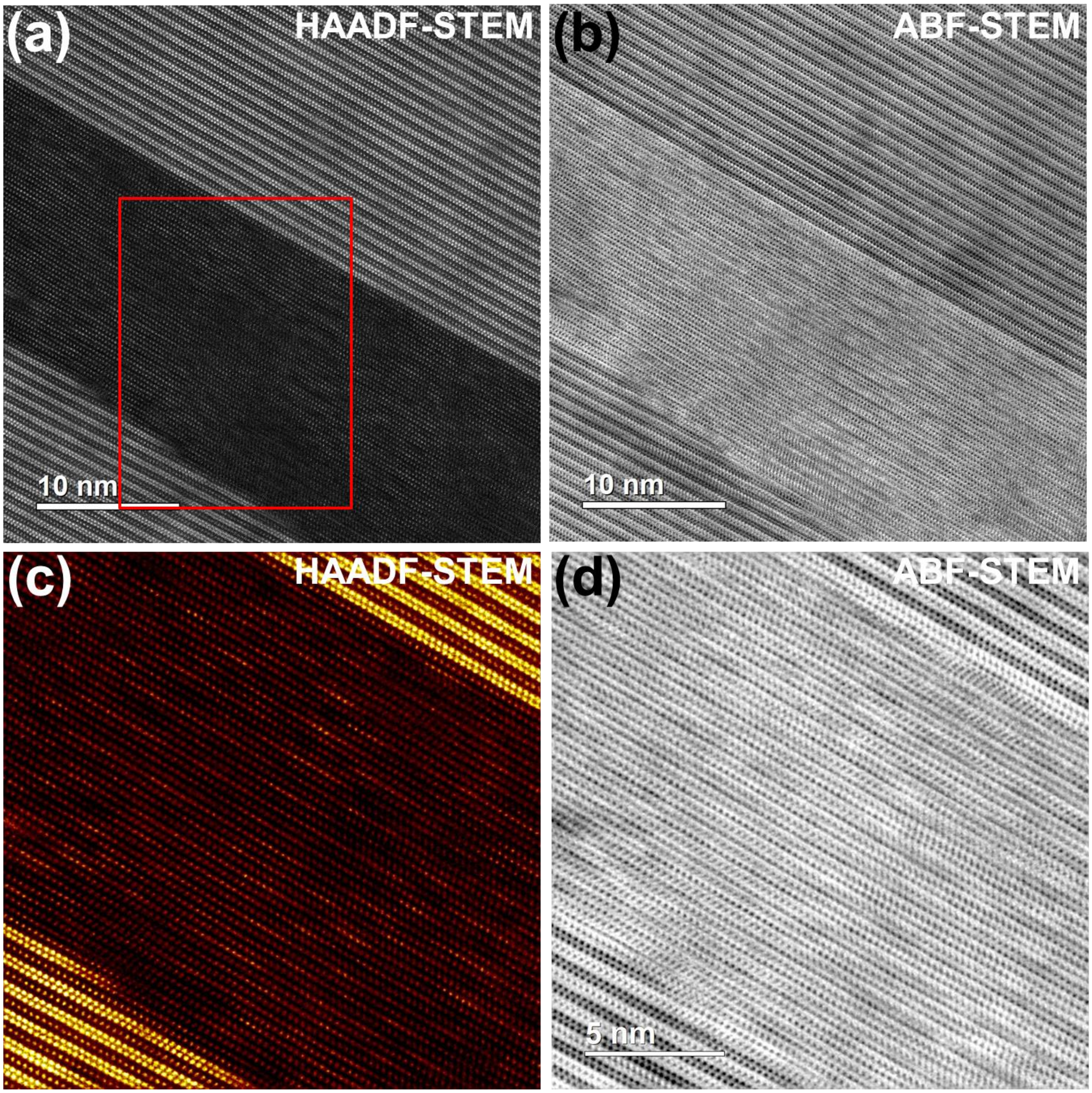

Supplementary Figure 22. High-resolution STEM imaging of monolayer and bilayer domains in global $Ag_2Ni_2TeO_6$ crystallite taken along [010] zone axis. Intergrowths (defects) entailing Ag-poor domains can be observed here. **(a)** Low-magnification HAADF-STEM image and **(b)** Corresponding ABF-STEM image. **(c)** High-magnification HAADF-STEM image for the region highlighted in (a) wherein Ag-poor domains were observed. **(d)** Corresponding ABF-STEM image.

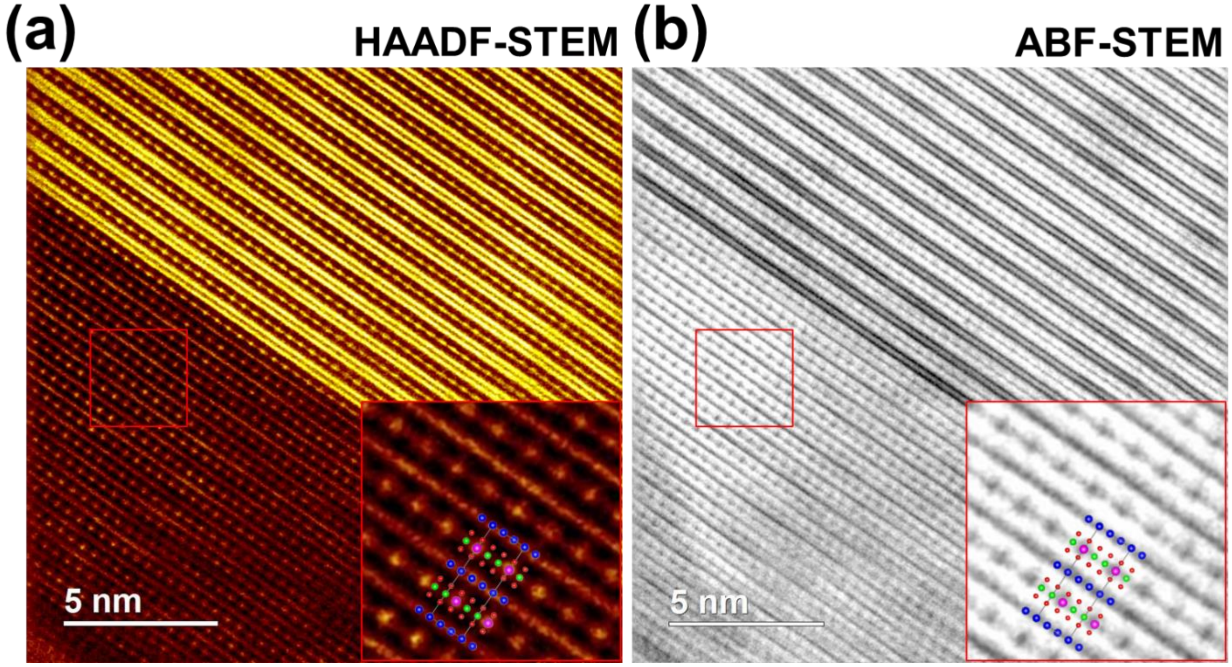

Supplementary Figure 23. High-resolution TEM imaging of monolayer and bilayer domains in global $Ag_2Ni_2TeO_6$ crystallite taken along the [100] zone axis. Intergrowths (defects) entailing Ag-poor domains can be observed here. **(a)** HAADF-STEM image and **(b)** Corresponding ABF-STEM image. The magnified image highlighted in red has been shown as inset. Ag atoms are shown in blue whilst Te and Ni atoms are shown in pink and green, respectively.

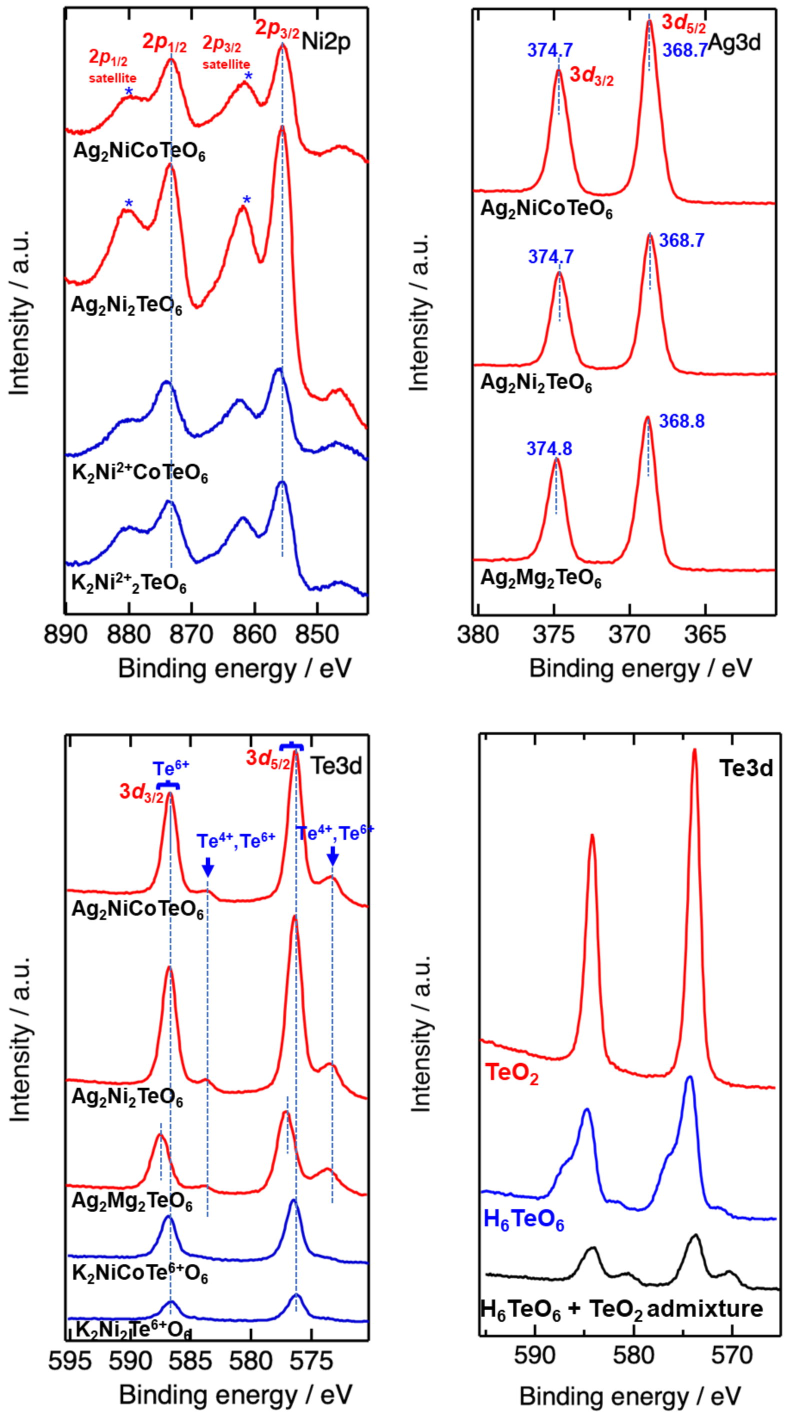

Supplementary Figure 24. X-ray photoelectron spectra of as-prepared $Ag_2Ni_2TeO_6$ and related derivatives ($Ag_2NiCoTeO_6$ and $Ag_2Mg_2TeO_6$) collected at the binding energies of Ag 3*d*, Te 3*d* and Ni 2*p* spectra along with reference compounds.

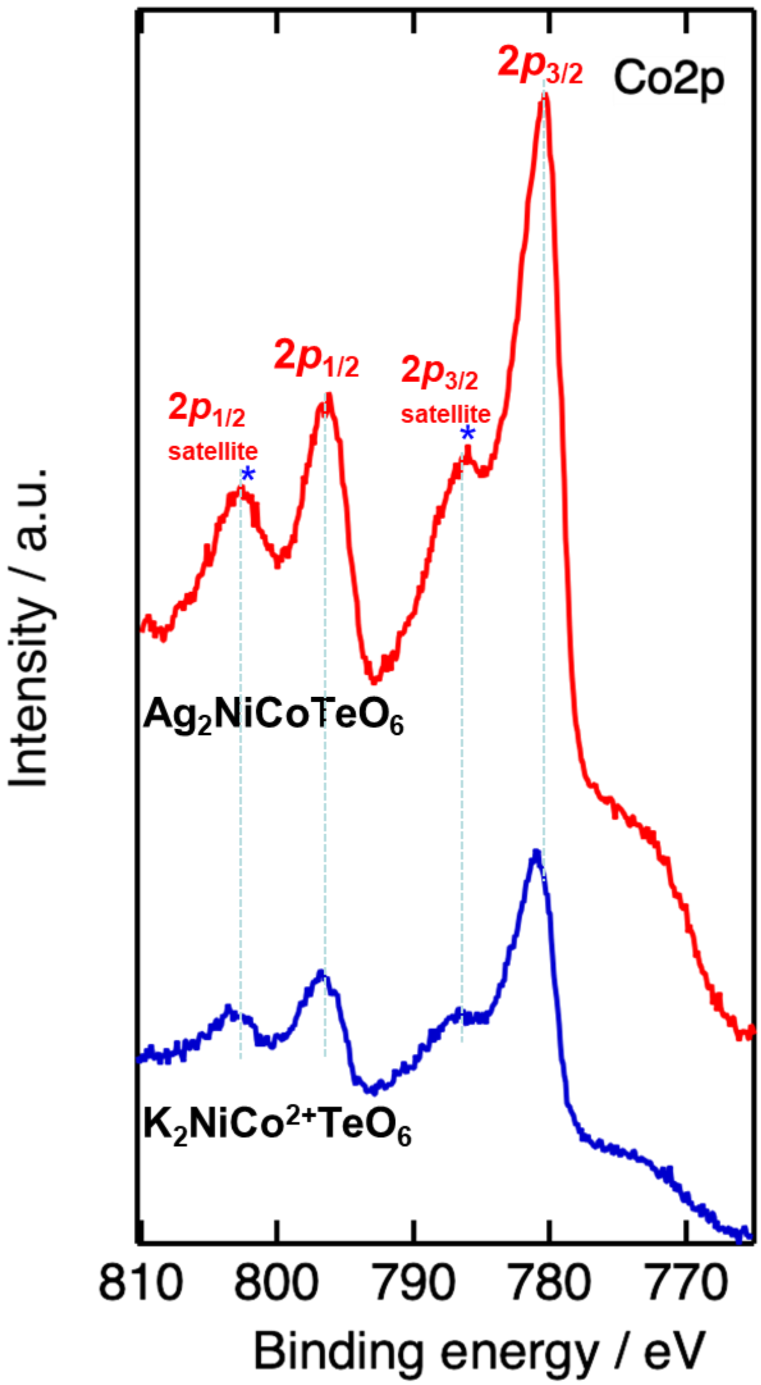

Supplementary Figure 25. X-ray photoelectron spectra of as-prepared $Ag_2NiCoTeO_6$ along with its reference compounds collected at the binding energies of Co 2*p* spectra.

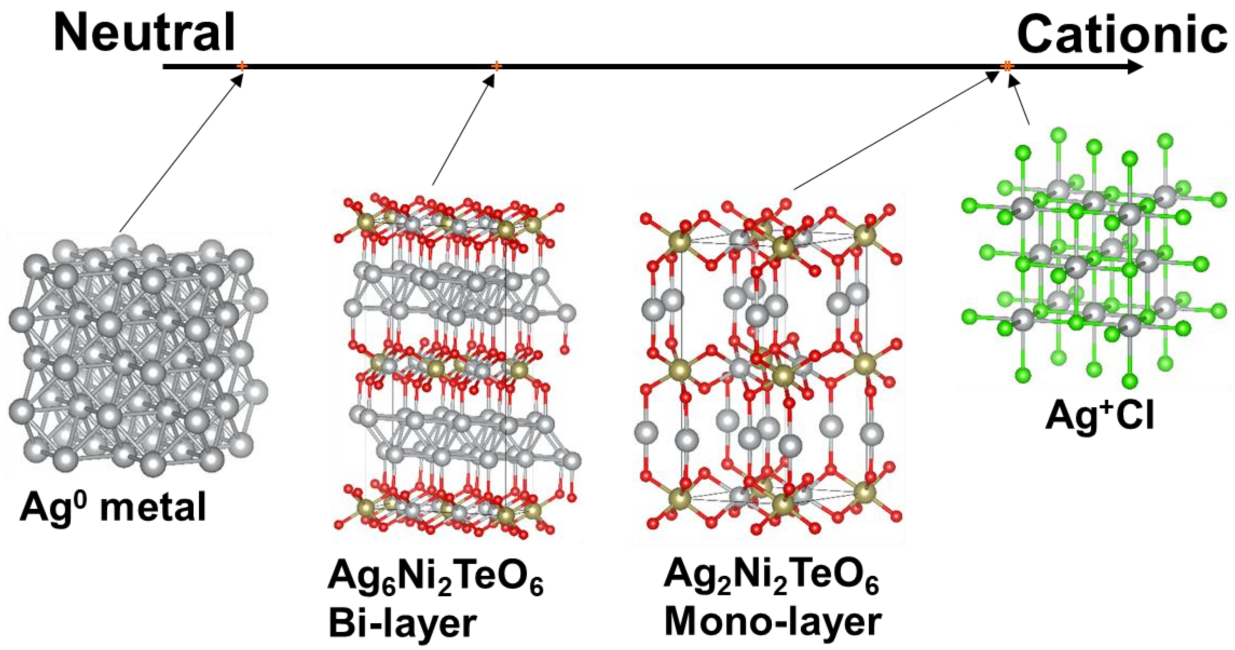

Supplementary Figure 26. Atomic Bader charges of Ag based on density functional theory calculations. Computational methodology is furnished in Supplementary Note 1. The horizontal axis corresponds to the number of valence electrons of Ag in atomic unit [e^-]. Specifically, the values of Ag metal, $Ag_6Ni_2TeO_6$ bi-layer model, $Ag_2Ni_2TeO_6$ mono-layer model, and AgCl are 11.000, 10.774, 10.402, and 10.400, respectively.

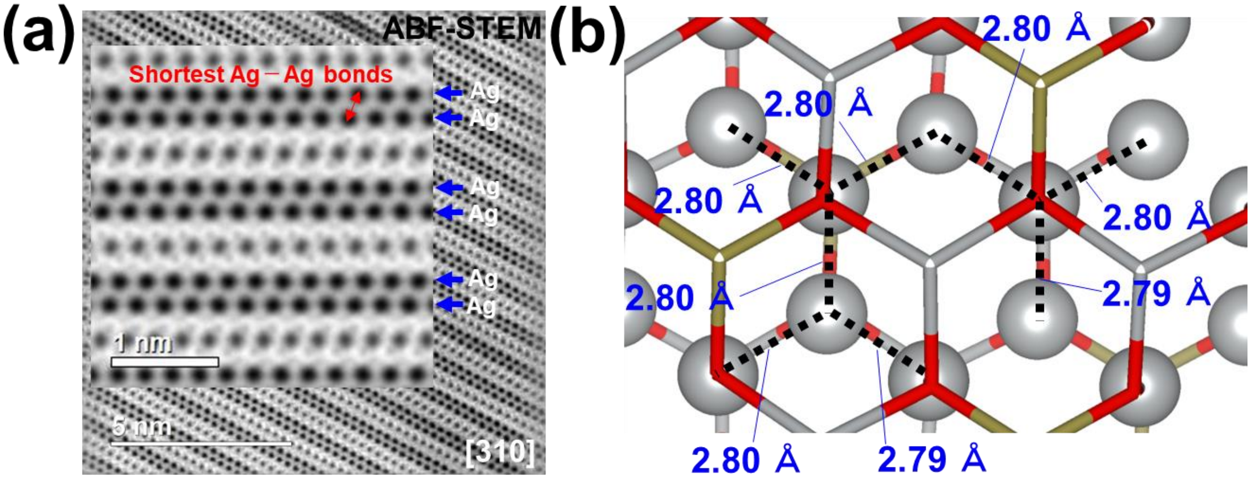

Supplementary Figure 27. Shortest Ag-Ag bond distances observed in bilayer $Ag_6Ni_2TeO_6$ domains. (a) High-resolution ABF-STEM imaging of Ag atoms in $Ag_6Ni_2TeO_6$. (b) Selected Ag-Ag bond distances attained from direct observation of the STEM images of $Ag_6Ni_2TeO_6$.

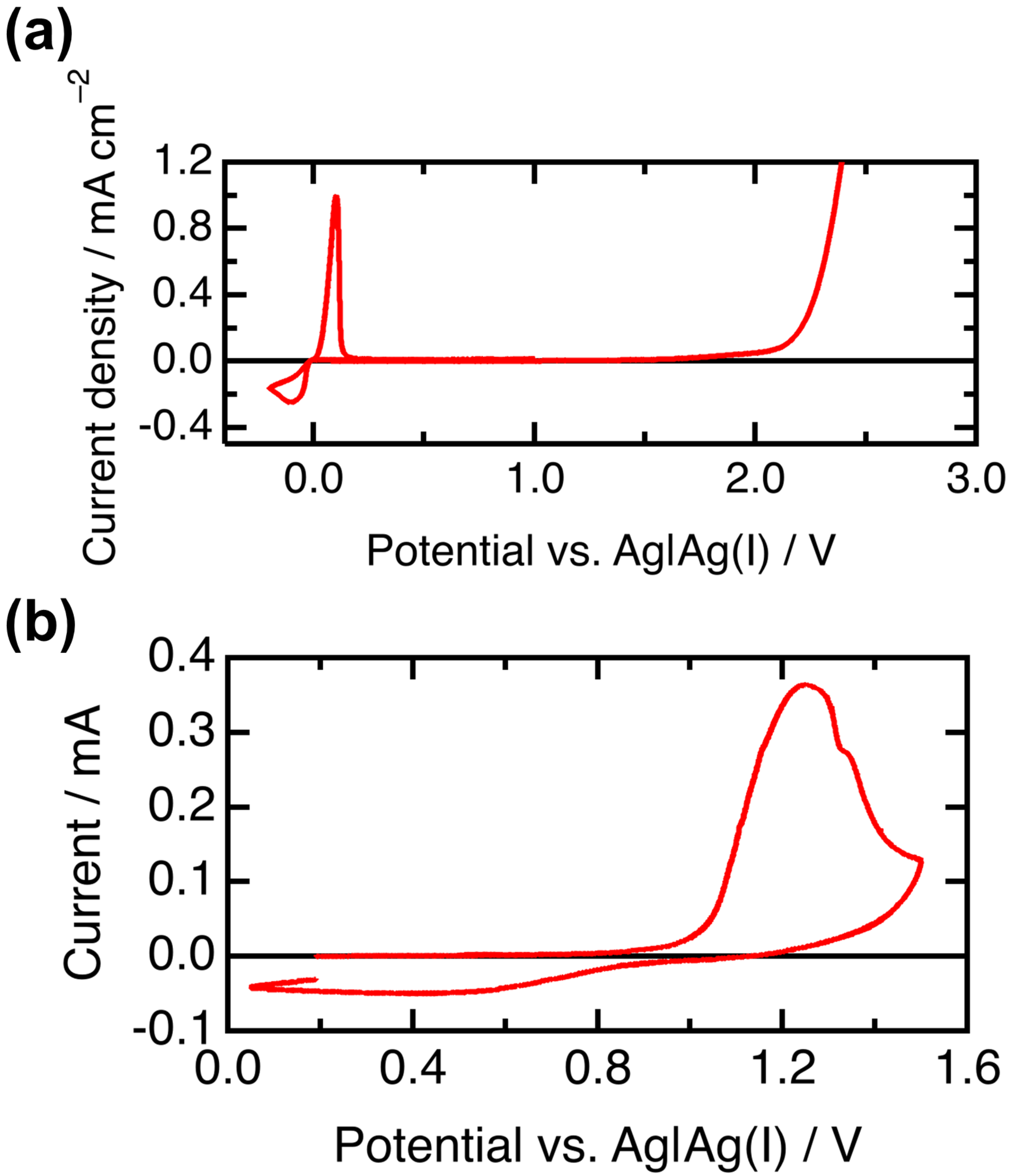

Supplementary Figure 28. Electrochemistry of global $Ag_2Ni_2TeO_6$ in Ag-half cells. **(a)** Linear sweep voltammetry of 0.1 M AgTFSA/Pyr_{13}TFSA ionic liquid at a scanning rate of 0.1 mV s^{-1}. Glassy carbon and platinum were used as substrates for the cathodic and anodic scan, respectively. Ag metals were used as reference and counter electrodes. **(b)** Cyclic voltammetry of $Ag_2Ni_2TeO_6$ using Ag half-cell configuration, under a scan rate of 0.1 mV s^{-1}.

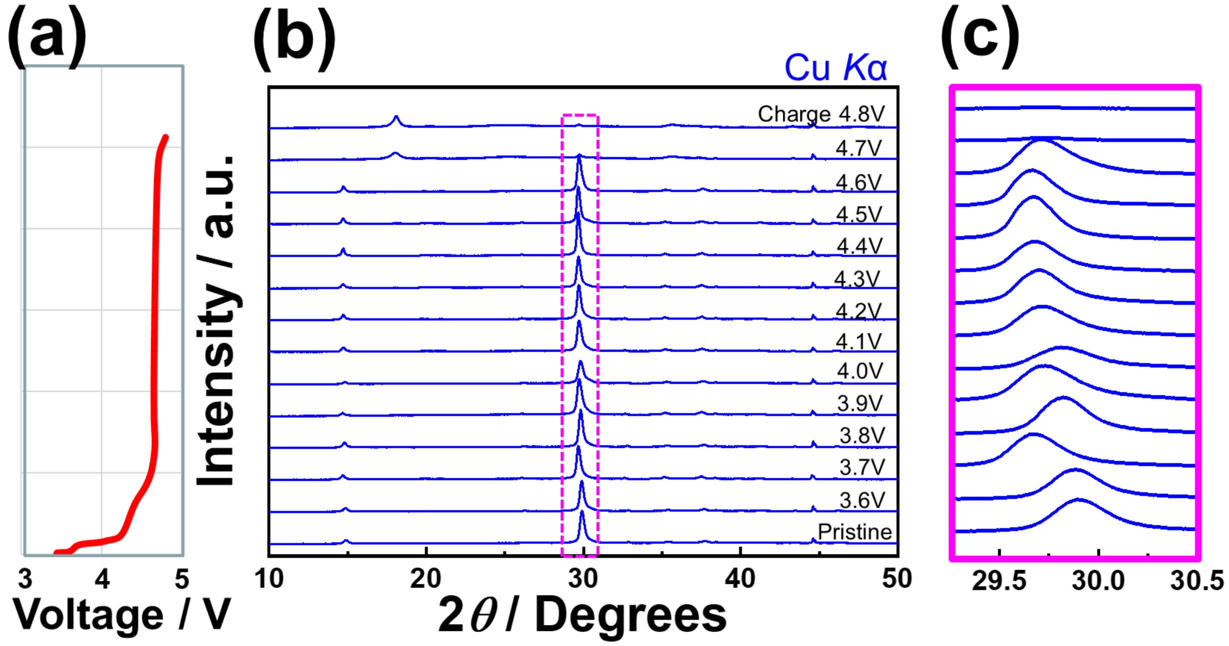

Supplementary Figure 29. Structural evolution of global $Ag_2Ni_2TeO_6$ upon Ag^+ extraction. **(a)** Voltage profile of $Ag_2Ni_2TeO_6$ upon charging (Ag^+ extraction) in Li half-cells. **(b)** XRD *ex situ* patterns of $Ag_2Ni_2TeO_6$ showing the structural evolution upon Ag^+ extraction/ deintercalation. **(c)** Enlarged image showing shifts of the Bragg diffraction peaks upon Ag^+ extraction.

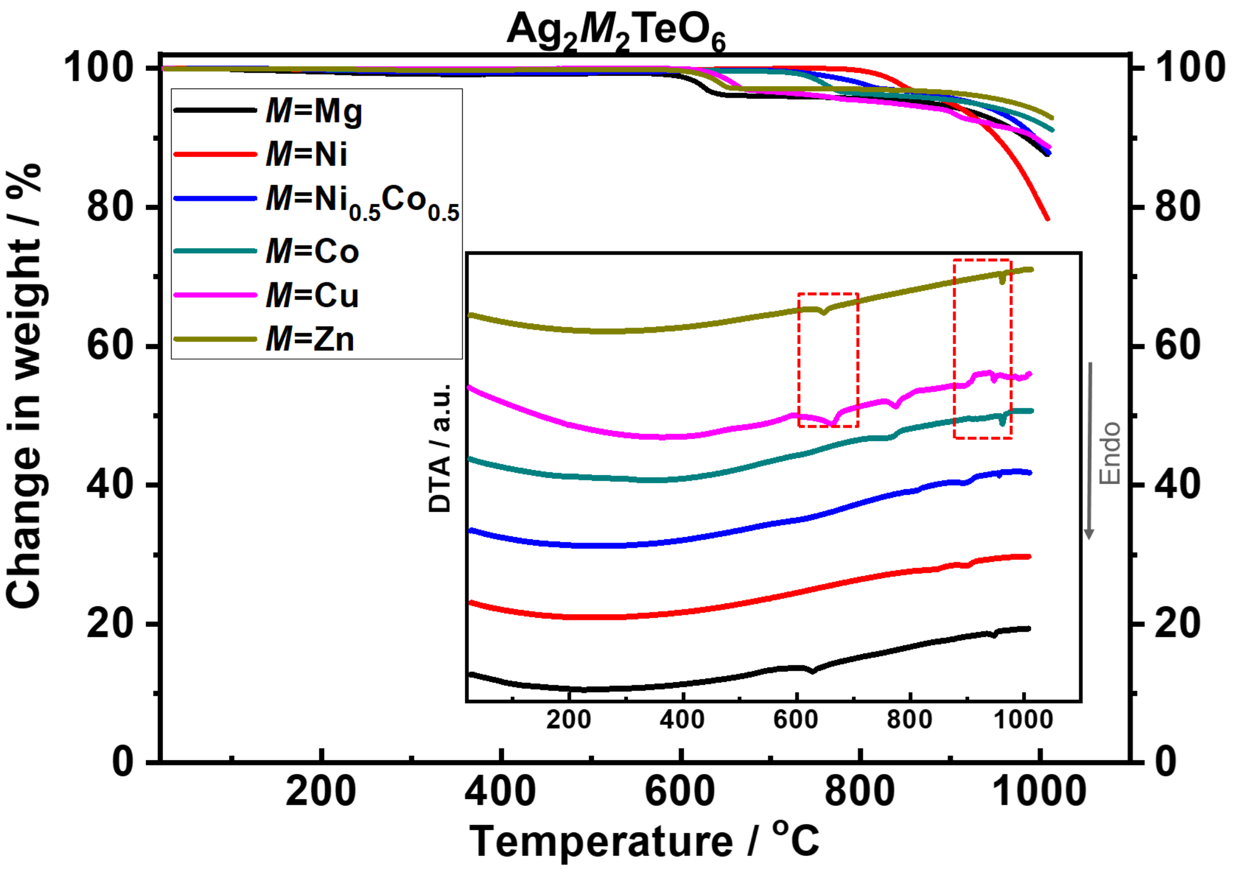

Supplementary Figure 30. Thermal stability of honeycomb layered oxides with a global composition of $Ag_2M_2TeO_6$ (M = Mg, Ni, $Ni_{0.5}Co_{0.5}$, Co, Cu, Zn). Thermogravimetry and differential thermal analysis (DTA) curve (inset) of $Ag_2M_2TeO_6$ (M = Mg, Ni, $Ni_{0.5}Co_{0.5}$, Co, Cu, Zn) ranging from 25 to 1000 ^{o}C. The onset of an endothermic peak at the DTA curve (denoted by asterisk) indicates the decomposition point or melting point of $Ag_2M_2TeO_6$.

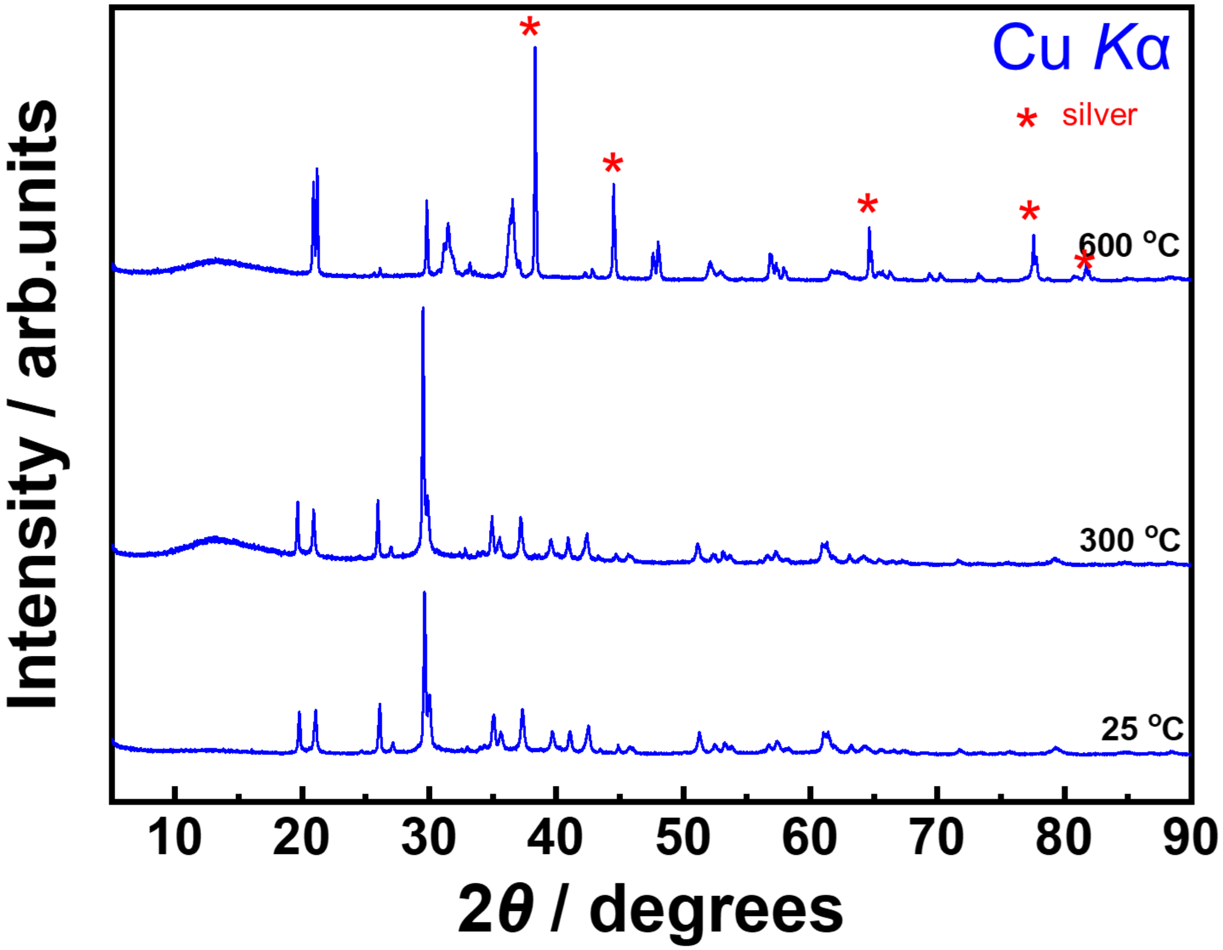

Supplementary Figure 31. Thermal Stability of global $Ag_2Mg_2TeO_6$. XRD patterns of $Ag_2Mg_2TeO_6$ were taken after annealing at various temperatures. Annealing at 600°C leads to the decomposition of $Ag_2Mg_2TeO_6$ to yield Ag metal (shown in asterisks) and other unknown phases.

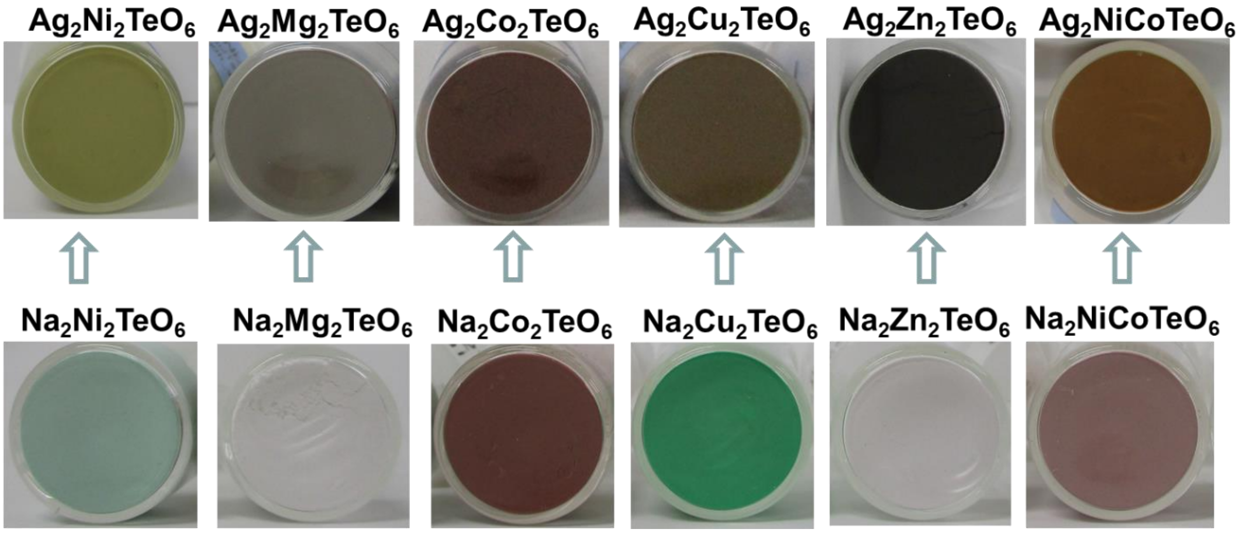

Supplementary Figure 32. Comparative illustration of precursor $Na_2M_2TeO_6$ (M =Ni, Mg, Co, Cu, Zn, $Ni_{0.5}Co_{0.5}$) material colours and their corresponding ion-exchanged Ag-based products with a global composition of $Ag_2M_2TeO_6$.

Supplementary Table 1. Composition stoichiometry of constituent elements in as-prepared $Ag_2M_2TeO_6$ (M = Ni, Mg, $Ni_{0.5}Co_{0.5}$, Co, Cu and Zn). Table showing the experimental stoichiometry values obtained from inductively coupled plasma atomic emission spectroscopy (ICP-AES) measurements of pristine $Ag_2M_2TeO_6$ (M = Ni, Mg, $Ni_{0.5}Co_{0.5}$, Co, Cu and Zn).

compound	Ag (mole ratio)	M (mole ratio)	Te (mole ratio)
$Ag_2Ni_2TeO_6$	2.06 ~ 2.12	2.00 ~ 2.01	1.00
$Ag_2Mg_2TeO_6$	2.15 ~ 2.18	1.95	1.00
$Ag_2NiCoTeO_6$	1.94	0.93 / 0.92	1.00
$Ag_2Co_2TeO_6$	1.95	1.82	1.00
$Ag_2Cu_2TeO_6$	1.98	1.94	1.00
$Ag_2Zn_2TeO_6$	1.88	1.79	1.00

Supplementary Table 2. Pycnometric density measurements of as-prepared $Ag_2M_2TeO_6$ (M = Ni, Mg, $Ni_{0.5}Co_{0.5}$, Co, Cu and Zn). Table showing the true density values of $Ag_2M_2TeO_6$ (M = Ni, Mg, $Ni_{0.5}Co_{0.5}$, Co, Cu and Zn) powders, obtained from pycnometric density measurements.

compound	batch 1 ($g\ cm^{-3}$)	batch 2 ($g\ cm^{-3}$)	batch 3 ($g\ cm^{-3}$)	Average ($g\ cm^{-3}$)
$Ag_2Ni_2TeO_6$	6.232	6.199	6.203	6.212
$Ag_2Mg_2TeO_6$	6.135	6.154	6.104	6.131
$Ag_2Co_2TeO_6$	6.110	6.119	6.116	6.115
$Ag_2NiCoTeO_6$	6.171	6.136	6.152	6.153
$Ag_2Cu_2TeO_6$	6.338	6.341	6.340	6.340
$Ag_2Zn_2TeO_6$	6.611	6.624	6.623	6.619

Supplementary Note 1. Computational methodology

The charge density for $Ag_2Ni_2TeO_6$, $Ag_6Ni_2TeO_6$, AgCl and Ag_2O were optimised to be self-consistent (with a threshold of 10^{-7} eV) using the Kohn-Sham formalism. [1] The Perdew-Burke-Ernzerhof expression (GGA-PBE), [2] which is one of the generalised gradient approximations, was adopted as the exchange-correlation functional. Further, a dispersion force correction of the DFT-D3 with Becke-Jonson damping method was adopted to accurately calculate the honeycomb oxide interlayer distance. [3,4] The inner core region was assessed using the projector-augmented-wavefunction method. [5] The number of valency electrons was set as follows: Ag ($4d^{10}5s^1$), Ni ($3d^8 4s^2$), Te($5s^2 5p^4$), Cl ($3s^2 3p^5$) and O ($2s^2 2p^4$). An on-site Coulomb correction (DFT+*U* method) was adopted, [6] with U = 4 eV added to the 3*d* orbital of Ni. The electronic spins on the Ni sites coupled in an anti-parallel manner can cause spin contamination error. Although the effect of the error in total energy was investigated by the approximated spin projection scheme for DFT, [7-9] the attained results do not affect the discussion presented herein. The DFT scheme can estimate the effects of orbital overlap, electrostatic interaction, charge-dipole interaction, and dipole-dipole interaction (dispersion force) when multi-configuration effects are ignored. The DFT calculations were performed by Vienna *Ab initio* Simulation Package (VASP) programme. [10-13]

Structural optimisation was performed for all atoms, lattice constants, and cell dimensions. The crystal structures were relaxed until the threshold was reduced below 10^{-5} eV or 10^{-2} eV/Å. Moreover, no symmetry restrictions were imposed during the structural optimisation. The energy cut-off was set at 500 eV (wavefunction) and 2400 eV (charge). *k*-sampling was performed with a 5×5×3 of Γ-centred mesh. The atomic charges for Ag in $Ag_2Ni_2TeO_6$, $Ag_6Ni_2TeO_6$, AgCl and Ag_2O were estimated by the Bader algorithm. [14] Renditions of the crystal structures were performed using VESTA crystallographic software. [15]

As shown in **Supplementary Figure 26**, Ag is sub-valent in $Ag_6Ni_2TeO_6$. Ag atoms are located in three non-equivalent sites, based on the position of Te. However, there is no significant difference in the number of valence electrons of the Ag located in the three sites (*viz*., 10.83 e^-, 10.77 e^-, 10.80 e^-), which is consistent with the discussion of the average values (10.80 e^-) shown in **Supplementary Figure 26**. One free electron is shared per two silver atoms in $Ag_6Ni_2TeO_6$, whereas two free electrons are shared per two silver atoms in Ag metal. Thus, the bond order of Ag-Ag in $Ag_6Ni_2TeO_6$ is formally half that of Ag metal. It should however be noted that this is a formal value that does not consider argentophilic interactions.

Supplementary Note 2. Conformal Field Theory (CFT) nature of the monolayer-bilayer phase transition

The existence of cationic Ag-atom bilayers observed in this study is reflective of an underlying *non-commutative (non-Abelian) algebra* emerging from a pseudo-spin structure. This suggests that the cationic diffusion within the bilayers necessitates a Hamiltonian acting on a quantum mechanical two-level system. As such, we should consider a Hamiltonian whose basis is a linear combination of SU(2) generators (Pauli matrices), wherein the matrices model the non-Abelian nature of interactions between each pair of silver atoms within a unit cell **(Manuscript Figure 4c).** In the present context, the relevant Hamiltonian responsible for such interactions within the Ag-atom bilayers in each unit cell is a 2×2 matrix given by,

$$H_{\text{int}} = \vec{B} \cdot \vec{\sigma}/2m = B_z \sigma_z/2m. \tag{1}$$

Here, $\vec{\sigma} = (\sigma_x, \sigma_y, \sigma_z)$ is the Pauli vector, m represents the mass of the cation, $\vec{B} = \vec{\nabla} \times \vec{A} = (0, 0, B_z)$ is a geometric curvature (playing the role of a pseudo-magnetic field) which can be considered to be a Berry curvature with the Berry connection, $\vec{A} = \ell(\vec{n} \times \vec{E})$ wherein, ℓ is the cut-off length scale along the z direction, $\vec{n} = (0, 0, 1)$ is the unit normal vector and $\vec{E} = \ell^{-1}\vec{\nabla}\Phi$ takes the role of the electric field.[16-18] Considering the symmetries observed in honeycomb lattices,[16-18] we define an emergent two-dimensional (2D) manifold with Gaussian curvature, K and a geometric potential, Φ that satisfies Liouville's equation,

$$\ell\vec{\nabla} \cdot \vec{E} = \nabla^2 \Phi = -K\exp(2\Phi) = B_z. \tag{2}$$

This not only means that the underlying metric is conformal,

$$ds^2 = \exp(2\Phi)(dx^2 + dy^2), \tag{3}$$

but also shows that the number of cations, k (equal to the number of cationic vacancies) is proportional to the Euler characteristic of an emergent manifold, $\chi(k)$ (Gauss-Bonnet theorem),[1]

$$\chi(k) = \iint B_z\, dxdy = \iint K \exp 2\Phi\, dxdy = \ell \iint \vec{\nabla} \cdot \vec{E}\, dxdy \propto k, \tag{4}$$

where the vacancy number density corresponds to B_z.

For further insight, we consider the honeycomb unit cell of the bilayer lattice of cations given in **Manuscript Figure 4c**. As illustrated, each unit cell is comprised of two cationic sites. The translation invariance of the unit cell [17] implies that the sides of the unit cell ought to be identified with each other, as shown in **Manuscript Figure 4d**. This corresponds to the existence of two states of the honeycomb lattice spanned by the unit cells: (i) a flat torus with Gaussian curvature vanishing everywhere on the 2D surface and (ii) a nil Euler characteristic ($K = 0, \chi = 0$) which can be mapped onto a 2D surface of a torus embedded in three-dimensional (3D) space with a vanishing Euler characteristic, but a finite Gaussian curvature ($K \neq 0,\ \chi = 0$).

In both cases, the Euler characteristic vanishes, indicating that this approach is consistent when there are no prior cationic vacancies in the layers ($\chi = k = 0$), in accordance with **equation 4**. Further, the two-level Hamiltonian given in **equation 1** suggests that each silver atom in the unit cell should be assigned an opposite pseudo-spin orientation, with their quantum states represented by a two-component *Pauli spinor.* This can be understood to stem from the discrete rotational symmetry of the unit cell of the honeycomb lattice.[17] In fact, both the rotational and

translation symmetries of the unit cell are *modular* in nature, captured by the so-called generators of the special linear group with integer entries and determinant 1 ($SL_2(Z)$), [17]

$$S = \begin{pmatrix} 0 & -1 \\ 1 & 0 \end{pmatrix}, \qquad T = \begin{pmatrix} 1 & 1 \\ 0 & 1 \end{pmatrix}. \tag{5a}$$

These 2×2 matrices operate on the basis vectors of the unit cell, whilst the Euler characteristic associated to the unit cell ought to transform as a *modular form* (of level 2) [17],

$$\chi(-1/k) = -k^2\chi(k), \qquad \chi(k+1) = \chi(k) \tag{5b}$$

which is satisfied by the torus with $\chi \propto 2k = 0$. Moreover, the eigenvalues for each cationic position in the unit cell correspond to $S^{2n} = \pm 1$, where $n \in N$ is a positive integer with $2n$ the number of discrete rotations. This means that the ground state of the honeycomb unit cell is two-fold degenerate, wherein each energy state is occupied by a single pseudo-fermionic (pseudo spin up/down) state occupied by a Ag cation (**Manuscript Figure 4c**). The pseudo-spins are arranged in alternating fashion along the honeycomb structure, thus preventing the occurrence of geometric (spin) frustration(s).[19] However, since the symmetries of the system ought to be shared by the ground state, assumed dominated by location dependent potential energies, the translation invariance of the unit cell compels all the connected cations to be at the same ground state energy, thereby introducing an energetically frustrated system in along the *z* direction (interpreted as an apparent violation of the Pauli-exclusion principle of the pseudo-spins). Nonetheless, we recognise that bosonic cationic states face no such geometric frustration since they can occupy the same ground state.

TEM images (**Manuscript Figure 1 and Figure 2**) of the Ag atoms show that their honeycomb lattice bifurcates, along the *z* coordinate, into two triangular lattices forming a bilayer which creates a vacancy at each site of the unit cell as shown in **Manuscript Figure 4c**. According to **equation 2**, this introduces a finite vacancy number density and hence a non-vanishing pseudo-magnetic field, $B_z \neq 0,$ where the corresponding torus is embedded in 3D. Thus, solving **equation 2**, we find that the potential energy separation of the bilayers according to the Pauli Hamiltonian in **equation 1** is given by,

$$\delta E \propto \frac{B_z}{m} = \frac{\Delta B_0}{m}\exp\left(\frac{2\Phi}{\Delta}\right) = -\frac{\Delta}{R^2}\ , \tag{6}$$

where the bifurcation requires the theory to no longer be in 2D (Theory must include the z coordinate). Here, $R = |(\vec{x}, \vec{y}, \vec{z})|$ denotes the displacement vector separating each cation in a honeycomb unit cell, B_0 is a constant with dimensions of the pseudo-magnetic field, $\Phi = -(\Delta/2)\ln(B_0R^2)$ is the geometric potential and $\Delta = (d-2)/2$ is taken to be a dimensionless constant playing the role of the conformal dimension with $d = 2$ or 3. Succinctly, Liouville's equation transforms into the well-known Emden–Chandrasekhar equation, which is 3D. This approach links the finite pseudo-magnetic field to the transition from 2D to 3D, and therefore justifies the description of the 2D flat torus with no Gaussian curvature transforming into the two-Torus embedded in 3D with a finite Gaussian curvature. Thus, the ground state is indeed degenerate with each pair of Ag atoms treated as a boson, which in turns opens an energy gap (**Figure 4b**) whose inverse serves as a correlation length scale.[20] This breaks modular symmetry (and hence conformal invariance) of each Ag layer ($\chi \propto k_\pm = \pm 1 \neq 0$ *violates* **equation 5b**) by introducing a single cationic vacancy at each unit cell, while preserving the overall modular symmetry of the bilayer ($\chi \propto k = k_+ + k_- = 0$ *does not violate* **equation 5b**).

The triumph of this formalism is tied to the fact that conformal symmetry is broken in 3D due to a finite correlation length scale brought forth by the Ag–Ag argentophilic interactions. In

particular, the conformal dimension, $\Delta = 0, 1$ acts as the order parameter responsible for the phase transition that determines whether the system can be considered 2D or 3D. Such interactions offer a pairing mechanism of the two pseudo-spin states of Ag atoms within a unit cell of the honeycomb lattice, forming pseudo-spin zero bound states and a resultant finite energy gap.

Supplementary References

1. Kohn, W. & Sham, L. J. Self-Consistent Equations Including Exchange and Correlation Effects. *Phys. Rev*. **140**, A1133 (1965).
2. Perdew, J. P., Burke, K. & Ernzerhof, M. Generalized Gradient Approximation Made Simple. *Phys. Rev. Lett*. **77**, 3865 (1996).
3. Grimme, S., Antony, J., Ehrlich, S. & Krieg, H. A consistent and accurate ab initio parametrization of density functional dispersion correction (DFT-D) for the 94 elements H-Pu. *J. Chem. Phys*. **132**, 154104 (2010).
4. Grimme, S., Ehrlich, S. & Goerigk, L. Effect of the damping function in dispersion corrected density functional theory. *J. Comput. Chem*. **32**, 1456 (2011).
5. Kresse, G. & Joubert, D, From ultrasoft pseudopotentials to the projector augmented-wave method. *Phys. Rev. B* **59**, 1758 (1999).
6. Dudarev, S. L. et al. Electron-energy-loss spectra and the structural stability of nickel oxide: An LSDA+U study. *Phys. Rev. B* **57**, 1505 (1998).
7. Tada, K., Koga, H., Okumura, M. & Tanaka, S. Estimation of spin contamination error in dissociative adsorption of Au_2 onto MgO (0 0 1) surface: First application of approximate spin projection (AP) method to plane wave basis. *Chem. Phys. Lett*. **701**, 103–108 (2018).
8. Tada, K. et al. Spin contamination errors on spin-polarized density functional theory/plane-wave calculations for crystals of one-dimensional materials. *Appl. Phys. Express* **12**, 115506 (2019).
9. Tada, K. et al. Estimation of spin contamination errors in DFT/plane-wave calculations of solid materials using approximate spin projection scheme. *Chem. Phys. Lett.* **765**, 138291 (2021).
10. Kresse, G. & Hafner, J. Ab initio molecular dynamics for liquid metals. *Phys. Rev. B* **47**, 558 (1993).
11. Kresse, G. & Hafner, J. Ab initio molecular-dynamics simulation of the liquid-metal–amorphous-semiconductor transition in germanium. *Phys. Rev. B* **49**, 14251 (1994).
12. Kresse, G. & Furthmüller, J. Efficient iterative schemes for ab initio total-energy calculations using a plane-wave basis set. *Phys. Rev. B* **54**, 11169 (1996).
13. Kresse, G. & Furthmüller, J. Efficiency of ab-initio total energy calculations for metals and semiconductors using a plane-wave basis set. *Comput. Mater. Sci*. **6**, 15–50 (1996).
14. G. Henkelman, A. Arnaldsson, and H. Jónsson, A fast and robust algorithm for Bader decomposition of charge density, *Comput. Mater. Sci*. **36**, 354-360 (2006).
15. Momma, K. & Izumi, F. VESTA 3 for three-dimensional visualization of crystal, volumetric and morphology data. *J. Appl. Crystallogr*. **44**, 1272–1276 (2011).
16. Kanyolo, G. M. & Masese, T. An idealised approach of geometry and topology to the diffusion of cations in honeycomb layered oxide frameworks. *Sci. Rep.* **10**, 13284 (2020).

17. Kanyolo, G. M. & Masese, T. Cationic vacancies as defects in honeycomb lattices with modular symmetries. *Sci. Rep.* **12** (1), 1-14 (2020)
18. Kanyolo, G. M. & Masese, T. Partition function for quantum gravity in 4 dimensions as a 1/N expansion. hal-03335930 (2021).
19. Toulouse, G. The frustration model. In: Pękalski A., Przystawa J.A. (eds) Modern Trends in the Theory of Condensed Matter. Lecture Notes in Physics, 1980, vol 115. Springer, Berlin, Heidelberg. https://doi.org/10.1007/BFb0120136
20. Domb, C. & Lebowitz, J. Phase Transitions and Critical Phenomena, Elsevier, Ltd, 2000. ISBN: 9780080538754
